# Inference of phenotype-defining functional modules of protein families for microbial plant biomass degraders

**DOI:** 10.1186/s13068-014-0124-8

**Published:** 2014-09-09

**Authors:** Sebastian GA Konietzny, Phillip B Pope, Aaron Weimann, Alice C McHardy

**Affiliations:** Max-Planck Research Group for Computational Genomics and Epidemiology, Max-Planck Institute for Informatics, University Campus E1 4, Saarbrücken, 66123 Germany; Department of Chemistry, Biotechnology and Food Science, Norwegian University of Life Sciences, Post Office Box 5003, 1432 Ås, Norway; Department of Algorithmic Bioinformatics, Heinrich Heine University Düsseldorf, Düsseldorf, 40225 Germany

**Keywords:** Latent Dirichlet allocation, LDA, Probabilistic topic models, (Ligno)cellulose degradation, Plant biomass degradation, Phenotype-based identification of functional modules, Pectin degradation, Feature ranking, Polysaccharide utilization loci, Gene clusters

## Abstract

**Background:**

Efficient industrial processes for converting plant lignocellulosic materials into biofuels are a key to global efforts to come up with alternative energy sources to fossil fuels. Novel cellulolytic enzymes have been discovered in microbial genomes and metagenomes of microbial communities. However, the identification of relevant genes without known homologs, and the elucidation of the lignocellulolytic pathways and protein complexes for different microorganisms remain challenging.

**Results:**

We describe a new computational method for the targeted discovery of functional modules of plant biomass-degrading protein families, based on their co-occurrence patterns across genomes and metagenome datasets, and the strength of association of these modules with the genomes of known degraders. From approximately 6.4 million family annotations for 2,884 microbial genomes, and 332 taxonomic bins from 18 metagenomes, we identified 5 functional modules that are distinctive for plant biomass degraders, which we term “plant biomass degradation modules” (PDMs). These modules incorporate protein families involved in the degradation of cellulose, hemicelluloses, and pectins, structural components of the cellulosome, and additional families with potential functions in plant biomass degradation. The PDMs were linked to 81 gene clusters in genomes of known lignocellulose degraders, including previously described clusters of lignocellulolytic genes. On average, 70% of the families of each PDM were found to map to gene clusters in known degraders, which served as an additional confirmation of their functional relationships. The presence of a PDM in a genome or taxonomic metagenome bin furthermore allowed us to accurately predict the ability of any particular organism to degrade plant biomass. For 15 draft genomes of a cow rumen metagenome, we used cross-referencing to confirmed cellulolytic enzymes to validate that the PDMs identified plant biomass degraders within a complex microbial community.

**Conclusions:**

Functional modules of protein families that are involved in different aspects of plant cell wall degradation can be inferred from co-occurrence patterns across (meta-)genomes with a probabilistic topic model. PDMs represent a new resource of protein families and candidate genes implicated in microbial plant biomass degradation. They can also be used to predict the plant biomass degradation ability for a genome or taxonomic bin. The method is also suitable for characterizing other microbial phenotypes.

**Electronic supplementary material:**

The online version of this article (doi:10.1186/s13068-014-0124-8) contains supplementary material, which is available to authorized users.

## Background

Lignocellulose is an integral part of plant cell walls. Its high energy content and renewability make it a promising alternative energy resource, particularly for the production of biofuels [1,2]. However, the current industrial methods of degrading recalcitrant plant cell wall material remain inefficient [3], which has created great interest in lignocellulolytic microbial organisms [4], because these represent a promising source of potential enzymes for improving industrial degradation processes [4,5]. Plant cell walls consist of cellulose and hemicelluloses (for example, xylan, xyloglucan, β-glucan), which are cross-linked by lignin, and pectins [6,7]. Cellulose is a macromolecule of β-(1,4)-linked D-glucose molecules. Xylans and β-glucans are homopolysaccharides composed of either xylose or β-1,3, β-1,4-linked D-glucose, respectively, and are commonly found in plant cell walls of grasses. Xyloglucan is a hemicellulose occurring in the plant cell wall of flowering plants, and consists of a glucose homopolysaccharide backbone with xylose side chains, which are occasionally linked to galactose and fucose residues. Pectin is a heteropolysaccharide that represents a major component of the middle lamella of plant cell walls, while lignin is a strongly cross-linked polymer of various aromatic compounds. Degradation of plant material requires the concerted action of different carbohydrate-binding modules (CBMs) and catalytic enzymes, such as cellulases, xylanases, pectin lyases and peroxidases [8-10]. The CAZy database [11] distinguishes four important subclasses of carbohydrate-active enzymes (CAZymes): glycoside hydrolases (GHs), glycosyltransferases (GTs), polysaccharide lyases (PLs), and carbohydrate esterases (CEs). However, cellulolytic enzymes can also be multifunctional, and combine several CAZy families in a modular architecture [12].

Microorganisms use different strategies to degrade recalcitrant plant material. The free enzyme and the cellulosome strategies are the strategies most widely used by known microbial plant biomass degraders [12,13]. The free enzyme paradigm is frequently used by aerobic bacteria, and involves the secretion of cellulolytic enzymes to degrade lignocellulose in the external medium. The cellulosome strategy has so far been described only for anaerobic bacteria [13], and involves large protein complexes (the cellulosomes) that incorporate cellulolytic enzymes, as well as CBMs for localized lignocellulose degradation [14]. The cellulosome includes a scaffoldin backbone to which cellulases and hemicellulases attach via cohesin–dockerin interactions. The corresponding (hemi)cellulases contain the dockerin domains, one or more catalytic domains (for example, GHs), and non-catalytic CBMs [14]. More recently, two additional strategies for (hemi)cellulose degradation have been outlined. The first strategy is the Sus-like protein system, which relies on mechanisms that are similar to the starch utilization (Sus) system in *Bacteroides thetaiotaomicron* [15,16]. These mechanisms are mediated by enzymes located in the outer membrane [17]. The second strategy involves the oxidative cleavage of cellulose by copper mono-oxygenases, a mechanism that increases the efficiency of the hydrolytic enzymes [18].

Certain cellulolytic organisms, such as *Fibrobacter succinogenes* and *Cytophaga hutchinsonii*, do not seem to use any of the currently known mechanisms [13]. Additional insights into microbial degradation processes have been generated by studies of microbial communities using metagenomics. This has led to the identification of thousands of putative carbohydrate-active genes [19,20] and of several novel genes encoding proteins with cellulolytic activities from uncultured organisms [21-23]. Overall, more than 1,000 cellulase genes have been discovered by genomic and functional screens [24]; however, important details about their degradation mechanisms remain unresolved [13,25]. Therefore, the discovery of novel protein families that are involved in plant biomass degradation is an ongoing effort.

The CAZymes Analysis Toolkit (CAT) can be used to recognize carbohydrate-active enzymes [26]. CAT deduces its prediction rules from the frequencies of modular proteins with Pfam and CAZy assignments in the CAZy database, thus its application to newly emerging sequences from metagenomes is likely to be limited because it is restricted to protein families that already have correspondences in the database. An alternative approach for determining the protein families that participate in a particular process but have no homologs with known activities is to use computational methods that assign families to a functional context. Depending on the granularity of the context, this approach allows narrowing down of the set of possible functions for an uncharacterized protein family. Applied to thousands of families on a large scale, this allows the *de novo* discovery of phenotype-defining protein families, genes, or entire functional modules [27].

Several methods for ranking genes or pathways by their assumed relevance for a certain phenotype have been described [28-37]. These methods measure the association of individual protein families [28], known pathways [29] or single nucleotide polymorphisms [30] with the presence or absence of phenotypes across a set of genomes. In some instances, the search space is limited to proteins in predicted operon structures [31] or to pairs of functionally coupled proteins [32].

We have previously described a family-centric method for the identification of protein families involved in lignocellulose degradation [28]. This method uses an ensemble of linear L1-regularized support vector machine (SVM) classifiers trained with the genome annotations of known lignocellulose-degrading and non-lignocellulose-degrading species. Similar methods use ranking approaches that are followed by a clustering step, whereby phenotype-associated families are grouped into modules based on their co-occurrence patterns across organisms, which are likely to indicate functional dependencies [33,34]. However, we suggest that the order of steps should be reversed, that is, functional dependencies between families should be detected first. This is because family-centric ranking methods may fail to detect moonlighting proteins [35] that are active in multiple processes, which could reduce the global correlation of their absence/presence profiles with the ability of the organisms to perform the target process.

By contrast, pathway-centric methods search for sets of functionally coupled protein families related to a specific phenotype. These methods use prior information about pathways from, for example, the Kyoto Encyclopedia of Genes and Genomes (KEGG) [36] or BioPath [29] databases in the form of organism-specific enzyme reaction networks based on enzyme classification (EC) numbers. The Network Instance-Based Biased Subgraph Search (NIBBS) searches for phenotype-associated edges in order to identify phenotype-related enzyme reactions in a KEGG-based network [37]. Similarly, MetaPath identifies subgraphs of a KEGG-derived network by assessing the statistical support of phenotype associations for every edge [36]. To date, there has been no application of pathway-centric methods to the study of lignocellulose degradation. Moreover, because of their focus on well-defined reaction networks, these methods have limitations for the analysis of metagenome samples, which often allow only partial metabolic reconstructions. Furthermore, species from newly sequenced microbial communities are likely to have a metabolism that is distinct from the metabolisms of well-studied model species, and the latter have been the basis for most of the currently described reaction networks. We are not aware of a pathway-centric method for inferring phenotype-associated functional modules that is applicable to metagenomes, and does not require prior knowledge about the underlying enzyme reaction networks or the target pathways. However, such a method would represent an important addition to computational metagenome analysis methods [38]. A possible solution could be the use of the aforementioned family-centric methods that cluster families into modules after determining their associations with the phenotype of interest.

An indication of the functional context for a protein family can be obtained by clustering families by their co-occurrences across genomes [39,40]. We have previously used latent Dirichlet allocation (LDA) [41], a Bayesian method, to infer 200 potential functional modules from 575 prokaryotic genomes [42]. The modules represent sets of co-occurring protein families that are likely to be involved in a common biological process, and cover a broad range of biochemical activities, including several known protein complexes, metabolic pathways, and parts of signal transduction processes. Overall, the modules show significant functional coherence, as indicated by a comparison with high-confidence protein–protein interactions from the Search Tool for the Retrieval of Interacting Genes/Proteins (STRING) database [43]. Here, we describe a new method based on LDA for determining the functional modules associated with microbial plant biomass degradation. This method detects relevant functional modules by the strength of their associations with the plant biomass degradation phenotype.

In the current study, we processed a large dataset of nearly 3,000 sequenced bacterial and archaeal genomes and taxonomic bins of 18 metagenomes. Based on the abundance estimates reported by Medie *et al*. [44] and Berlemont *et al*. [45], the relative abundance of species possessing plant biomass degradation capabilities within the sequenced genomes could exceed 20 to 25%; however, to date, only a small set of species have been confirmed to possess such capabilities [4]. With our method, genomes of both known and unknown degraders could be included in the inference process, and be used to identify distinct sets of protein families that are specific for microbial plant biomass degraders. The use of metagenome data allows us to incorporate information from environmental communities into the inference process. We identified five functional modules for plant biomass degradation, which we call “plant biomass degradation modules” (PDMs). The PDMs found included many protein families that are known to be involved in plant biomass degradation, and a substantial number of families that have not previously been linked to microbial plant biomass degradation. To verify the relevance of these newly identified PDMs and candidate families, we searched for gene clusters including the families of the PDMs. Several of the identified clusters are known to be active in the degradation of lignocellulose. Furthermore, the PDMs had a predictive value for identifying plant biomass degraders from the genomes of sequenced isolates or of plant biomass-degrading microbial communities.

## Results and discussion

**Figure 1:**
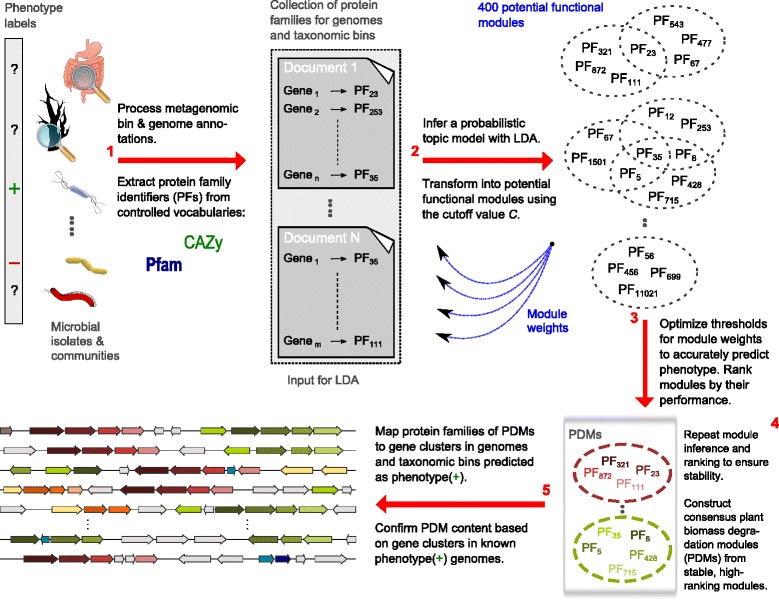
**Identifying phenotype-related functional modules.** We used protein sequences from 2,884 prokaryotic isolate species and 18 microbial communities, some of which are known to be involved in lignocellulose degradation. Known lignocellulose degradation abilities are indicated by phenotype labels (positive/negative: +/−). For the metagenomes, we considered only protein-coding sequences with predicted taxonomic origins assigned by a taxonomic binning method (*PhyloPythia* or *PhyloPythiaS)*. We used HMMER to assign protein family annotations from Pfam and CAZy to all input sequences, and summarized the set of (meta-)genome annotations as a document collection for LDA **(1)**. Each document represented a single genome or a metagenome bin, and was composed of protein family identifiers from a controlled vocabulary (Pfam, CAZy). We then inferred a probabilistic topic model **(2)**. The topic variables of the model can be interpreted as potential functional modules, that is, sets of functionally coupled protein families [42]. We obtained 400 modules with diverse biochemical functions. Next, we defined genome-specific weights of the modules, and used these weights in conjunction with the phenotype labels to rank the modules according to their estimated relevance for the phenotype of lignocellulose degradation **(3)**. As weights, we used the fraction of protein families in a module that were present in a certain genome or metagenome bin (completeness scores). We identified stable, high-ranking modules from independent repetitions of the analysis, and constructed consensus modules, which we named “plant biomass degradation modules” (PDMs) **(4)**. These PDMs were found to cover different aspects of plant biomass degradation, such as degradation of cellulose, hemicellulose, and pectin. Moreover, the weights of the PDMs could be used to predict the biomass degradation abilities of organisms, and we were able to identify specific gene clusters in the input set of (meta-)genomes that reflected the protein family content of individual modules **(5)**. The clusters thus provided evidence for the functional coherence of the modules by gene neighborhood.

We generated approximately 6.4 million protein annotations with Pfam and CAZy families for 2,884 bacterial and archaeal genomes from the Integrated Microbial Genomes database (IMG) and 332 taxonomic bins from 18 metagenomes (see Methods). We then used a two-step approach to identify functional modules that are distinctive for microbial lignocellulose degraders. First, the set of protein family annotations was processed with LDA, and 400 potential functional modules were inferred, with each corresponding to a set of Pfam and/or CAZy families (Figure 1, steps 1 and 2). The modules were learned in an unsupervised fashion without consideration of the phenotypes of the organisms, as described previously [42]. In the second step, we ranked the 400 functional modules according to their strength of association with the genomes of plant biomass degraders across a subset of the genomes consisting of 38 known lignocellulose degraders and 82 non-degraders (Figure 1, step 3). For this, we defined genome-specific module weights, which corresponded to the fraction of the protein families of a module that were annotated for a certain genome or taxonomic bin (completeness scores). Functional modules were considered to be present in a genome or bin if their completeness score reached a certain threshold. For each module, we determined the best setting for this threshold, corresponding to the one that optimally separated the genomes of degraders and non-degraders according to the F-measure (the weighted harmonic mean of precision and recall (see Methods)). The modules with the largest F-scores were strongly associated with the genomes of lignocellulose degraders, as indicated by an average F-score of 87.45% for the top 10 modules.

### Identification of stable PDMs

The implementation of the LDA model that we used was based on Gibbs sampling, a Markov chain Monte Carlo (MCMC) method that efficiently estimates parameters for complex models such as LDA. In agreement with the recommended procedures for MCMC sampling [46], we repeated the analysis multiple times (18 LDA runs) to ensure the stability of the results. We thus repeated the two central steps of our method, that is, the inference of modules and their subsequent ranking by phenotype association (Figure 1, steps 2 and 3), 18 times to identify stable, high-ranking modules. We summarized the information from stable, high-ranking modules found in different runs by constructing consensus modules that contained all the protein families found in similar modules in at least nine LDA runs (Figure 1, step 4; see Methods).

We identified five consensus modules (M1 to M5), which we referred to as PDMs (Table 1; see Additional file 1: Tables S1A-5A). We mapped the CAZy families of these PDMs to essential activities in the degradation of plant cell wall material, based on their EC numbers (Table 2). All PDMs included protein families with cellulase or hemicellulase activities, which supports the relevance of these modules for plant biomass degradation. M1 to M5 were functionally distinct, with only a moderate overlap (12.6%) of their protein family content, including the broadly defined families GH5 and GH43 [47]. Isofunctional Pfam and CAZy terms, such as PF00150 and GH5, were grouped together into the same PDMs in most cases. The modules also included 20 Pfam families without a commonly known link to plant biomass degradation, such as domains of unknown function (DUF), ricin-type β-trefoil lectin-like domains, and GDSL-like lipase/acylhydrolase (Table 3; see Additional file 2: Section 1). Some of these domains could encode novel functions that are important for plant biomass degradation.

**Table 1:** **Functional characterization of the consensus plant biomass degradation modules M1 to M5**

**Module** ^**a,b**^	**Description**
M1	Lignocellulose degradation (cellulose and hemicellulose degradation)
M2	Xylan binding and xyloglucan degradation (hemicellulose degradation)
M3	Pectin degradation
M4	Degradation of glycan compounds
M5	Structural components of the cellulosome-based degradation paradigm (dockerin and cohesin)

^a^We characterized each module based on the set of protein families contained within it.

^b^Additional file 1 shows each consensus module as a list of Pfam/CAZy terms (Tables S1A-5A).

**Table 2:** **CAZymes with key functions for plant cell wall degradation in the plant biomass degradation modules**

**Type**	**Subtype**	**Module**				
		**M1**	**M2**	**M3**	**M4**	**M5**
Cellulases [8]	Endoglucanase (EC 3.2.1.4)	GH5, GH9	–	GH5	GH5	GH124
	Cellobiohydrolase (EC 3.2.1.91)	GH5, GH9	–	GH5	GH5	–
	β-glucosidase (EC 3.2.1.21)	GH9	GH30	–	GH3	–
Hemicellulases [8,9]	Endo-1,4-β-xylanase (1,4-β-d-xylan xylanohydrolases, EC 3.2.1.8)	GH5, GH10, GH43	–	GH5, GH43	GH5, GH43	–
	β-xylosidase (1,4-β-d-xylan xylohydrolase, EC 3.2.1.37)	GH43	GH30	GH43	GH3, GH43	–
	α-arabinofuranosidase (EC 3.2.1.55)	GH43	–	GH43	GH3, GH43	–
	α-glucuronidase (EC 3.2.1.139)	–	–	–	–	–
	Acetyl xylan esterase (EC 3.1.1.72)	–	–	CE6, CE7, CE12	–	–
	Ferulic acid esterase (EC 3.1.1.73)	–	–	–	–	–
	Xyloglucanase (EC 3.2.1.151); xyloglucosyltransferase (EC 2.4.1.207)	–	GH16	–	–	–
Carbohydrate-binding modules [48,49]	Targeting cellulose	CBM4	–	–	–	CBM3
	Targeting xylan	CBM4, CBM6, CBM35	CBM6, CBM13, CBM35			CBM36
Cellulosomes [14]	Structural components	–	–	–	–	Cohesin, dockerin
Pectinolytic enzymes [10]	Pectin methyl esterase (EC 3.1.1.11)	–	–	CE8	–	–
	Endopolygalacturonase (EC 3.2.1.15); exopolygalacturonase (EC 3.2.1.67)	–	–	GH28	–	–
	Endopolygalacturonase lyase (EC 4.2.2.2); exopolygalacturonase lyase (EC 4.2.2.9)	–	–	PL1, PL9	–	–
	Pectin lyase (EC 4.2.2.10)	–	–	PL1	–	–

**Table 3:** **Protein families of the modules M1 to M5 with potential functions in plant biomass degradation**

**Module**	**Family ID**	**Description**
M1	PF13472^a^	GDSL-like lipase/acylhydrolase family
	PF00756^a^	Putative esterase
M2	PF14200^a,b^	Ricin-type β-trefoil lectin domain-like
	PF00652^a,b^	Ricin-type β-trefoil lectin domain
	PF00754^a^	F5/8 type C domain
	PF00041^a^	Fibronectin type III domain
	PF02311	AraC-like ligand-binding domain
	PF13483	Beta-lactamase superfamily domain
M3	PF03629^a,b^	DUF303
	PF00657^a^	GDSL-like lipase/acylhydrolase
	PF13472^a^	GDSL-like lipase/acylhydrolase family
	PF13229^a^	Right-handed beta helix region
M4	PF14310^a^	Fibronectin type III-like domain
	PF07859	Alpha/beta hydrolase fold
	PF00135	Carboxylesterase family
	PF13802	Galactose mutarotase-like
M5	PF13186^a,b^	DUF4008
	PF05593	RHS repeat
	PF07591	Pretoxin Hint domain
	PF07238	PilZ domain
	PF13403	Hint domain

DUF, domain of unknown function; GDSL, a motif in the amino acid sequences of the members of this protein family; PDM, plant biomass degradation module.

^a^Protein families appearing in the gene clusters identified by mapping the PDMs to the phenotype-positive genomes.

^b^Some potential functions of the families PF14200, PF00652, PF03629, and PF13186 are discussed in the context of the respective PDMs (see Results section; see Additional file 2: Section 1).

The table lists protein families of the plant biomass degradation modules (PDMs) that had no commonly known functions in plant biomass degradation. Every second family (55%) occurred in the gene clusters that were identified based on the PDMs. Note that family PF13472 was part of M1 and M3.

### Gene clusters with PDM protein families

To confirm a functional context for the protein families assigned to the same PDM, we searched for gene clusters annotated with multiple families of a PDM in the 38 genomes of known degraders, as the proximity of genes within a genome indicates a shared functional context [50,51] (Figure 1, step 5). For each PDM, we identified gene clusters of four or more neighboring genes, with intergenic distances of 2 kb or less between consecutive genes. Overall, 81 gene clusters were found for the 5 PDMs, which represented 51 distinct, non-overlapping clusters. The average distance between the genes of these clusters was 340 bp. On average, 70.7% of the family content of each PDM could be mapped to gene clusters in known degraders. Some of the gene clusters discovered have been described previously as being active in lignocellulose degradation (see following sections), whereas the novel gene clusters are candidates for further experimental investigation. Notably, more than half (55%) of the possibly relevant protein families in Table 3 appeared in at least one gene cluster identified in known degrading species, which supports their potential role in the degradation process.

### Assessment of the potential of the PDMs to predict unknown lignocellulose degraders

The completeness of a PDM in a genome was predictive for the ability of an organism to degrade lignocellulosic plant biomass. We determined the predictive value for each PDM by standard evaluation protocols in leave-one-out (LOO) and 10-fold cross-validation experiments (see Methods). In these experiments, genomes from the learning set of 120 known lignocellulose degraders and non-degraders were successively left out of the process of determining the completeness threshold. Subsequently, PDMs were predicted to be present in the omitted genomes if their completeness score for the genome was greater than or equal to the inferred threshold. This procedure was used to assess the generalization error of a PDM-based classifier to avoid overly optimistic performance estimates [52,53]. We observed high F-scores for the PDMs in the LOO setting (82.1 to 96.2%) and lower bounds for the cross-validation estimates of prediction accuracy between 76.57% and 91.69% (Table 4).

**Table 4:** **Association with lignocellulose degradation based on different performance measures for the consensus PDMs M1 to M5**

	**Module**				
	**M1**	**M2**	**M3**	**M4**	**M5**
Set of recurring modules (18 repetitions of analyses)					
Number of modules in set	18	18	18	18	16
Average F_0.5_-score in rankings, %	95.2 ± 1.7	92.5 ± 1.1	88.9 ± 2.1	85.8 ± 1.3	84.9 ± 5.3
Average rank	1.3 ± 0.57	2.4 ± 0.61	4.2 ± 1.5	6 ± 1.6	7.5 ± 3.4
Consensus PDM					
Size	18	22	23	25	13
Weight threshold used for classification, %	66.67	50.00	73.91	72.00	38.46
Performance evaluation					
LOO F_0.5_-score, %	96.2	94.1	89.6	82.5	82.1
LOO recall, %	92.1	84.2	63.2	84.2	57.9
LOO precision, %	97.2	97.0	100.0	82.1	91.7
CV accuracy, %	96.7	93.8	87.7	89.6	84.3
Estimated 95% confidence interval for CV accuracy	[91.69, 99.08]	[87.82, 97.35]	[80.42, 92.96]	[82.68, 94.42]	[76.57, 90.32]
CV-MAC, %	95.4	91.3	81.2	88.2	77.2

CV, cross-validation; LOO, leave-one-out; MAC, macro-accuracy; PDM, plant biomass degradation module.

Each consensus PDM represents a set of recurring modules from 18 independent repetitions of our analysis (Figure 1), and contains all families that occurred in at least nine of these modules. The recurring modules used to build the PDMs were identified by finding modules having minimal pairwise distances from each other (see Methods). We reported the average rank and average F-score of these module sets (F_0.5_ puts stronger emphasis on precision; that is, it weights recall as half as strongly as precision [54]; see Additional file 3: Section 3). “Size” gives the number of Pfam and/or CAZy families that are contained in a PDM. We computed recall, precision, and the F-measure scores for the individual PDMs in LOO validation. In addition, accuracies and estimated confidence intervals for 10-fold cross-validation (CV) were used to assess the generalization error more accurately. Following our previous study [28], we also computed the cross-validation macro-accuracy (CV-MAC) as the average of the true-positive (TP) and true-negative (TN) rates.

**Figure 2:**
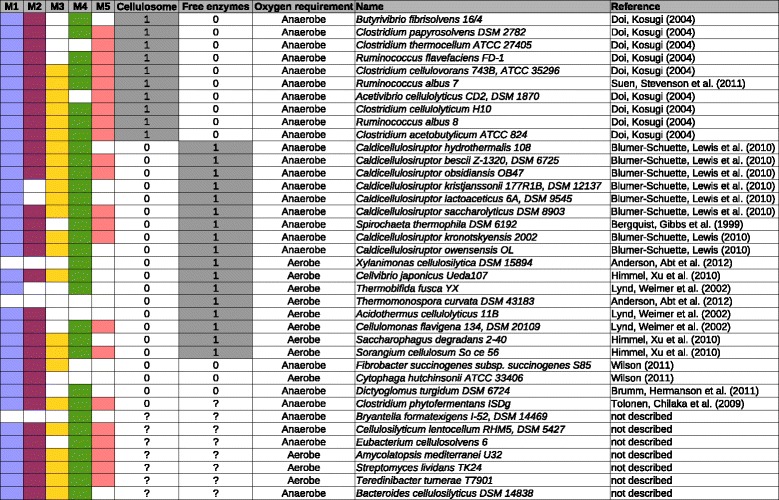
**Occurrences of plant biomass degradation modules (PDMs) in organisms using different degradation paradigms.** The predicted occurrences of the PDMs M1 to M5 in the genomes of 38 known lignocellulose degraders are indicated by different colors. Each PDM was predicted to be present or absent from a genome, depending on its genome-specific weight, that is, the degree of completeness for its protein families. Two major cellulose degradation paradigms – the free enzyme and cellulosome-based strategies – were assigned to the organisms according to the literature. Assignments can be ambiguous; for example, *Clostridium thermocellum* seems to be able to use mixed strategies [47]. No PDM was exclusively associated with these two paradigms, including M5, which, in addition to the cohesin and dockerin domains of cellulosomes, also included non-cellulosomal protein families (Table 3).

The top-ranking PDMs, M1 and M2, predicted the ability to degrade lignocellulose with cross-validation accuracies of more than 93%. Four genomes were misclassified by both M1 and M2 (see Additional file 4: Figure S1; see Additional file 5: Tables S1 and S2): *Bryantella formatexigens* (false negative (FN)), *Xylanimonas cellulosilytica* (FN), *Thermonospora curvata* 43183 (FN), and *Actinosynnema mirum* (false-positive (FP)). Interestingly, *A. mirum* and *T. curvata* might have been mischaracterized previously [55], supporting the predictions by the two PDMs (see Additional file 2: Section 2). All PDMs showed a precision of more than 82% for lignocellulose degraders, with few occurrences predicted for non-degraders. M3 and M5 were found only in a subset of the known degraders, with the lowest recall being 57.9% (Table 4), suggesting that these modules might represent specific aspects of degradation strategies. However, looking at the presence/absence profiles of the PDMs across the degrading species, none of the PDMs showed an exclusive association with a known degradation paradigm (Figure 2).

### Protein families of the PDMs

The highest-scoring PDM M1 (F-measure 96.2%) incorporated various key families for the degradation of cellulose and hemicelluloses (Table 2): GH5, GH9, GH10, GH26, GH43, and CBM6 [47]. The GH5 and GH9 families together cover three classes of important cellulases [8]: endoglucanases, cellobiohydrolases, and β-glucosidases. Both are large families of cellulases that have been studied in many lignocellulolytic organisms (see Additional file 2: Section 3). In addition to their cellulase activities, some members of these families are also hemicellulases with characterized activity on β-glucans, xyloglucans, and heteroxylans [11]. The GH10 and GH43 families include xylanases and arabinases. M1 was present almost exclusively in lignocellulose-degrading bacteria (97.2% precision) and in almost all of them (92.1% recall). Similarly, also the individual modules used for creating the M1 consensus PDM showed strong associations with plant biomass degradation: M1 was always among the three best-ranking modules, and was the top-ranked module in 14 of 18 LDA runs.

**Figure 3:**
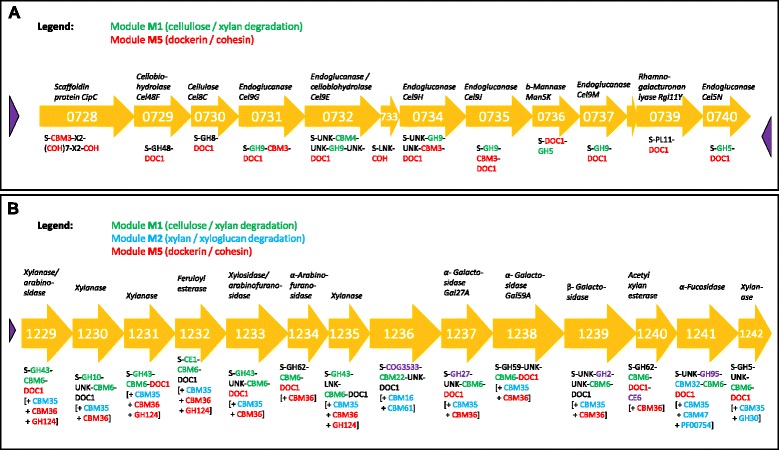
**Plant biomass degradation modules (PDMs) mapping to the** ***cel-cip*** **and** ***xyl-doc*** **gene clusters in** ***Clostridium cellulolyticum*** **H10.** Blouzard *et al.* described two clusters of genes that are involved in cellulose and hemicellulose degradation [57], and we adopted their domain architecture in this study. Abbreviations used for the carbohydrate-binding module (CBM) and glycoside hydrolase (GH) architecture are S, signal sequence; DOC1, dockerin type-I module; COH, cohesin type-I module; LNK, linker sequence; UNK, unknown function. We marked additional predicted domains as part of our in-house annotation sets using [+family X]. Some dockerin annotations were filtered out by our bit score criterion. **(A)** Genes from the *cel-cip* operon (Ccel_0728 to Ccel_0740) are essential for the cellulose degradation ability of the organism *C. cellulolyticum* H10, which uses the cellulosome strategy*.* The cluster includes multiple protein families of the PDMs M1 and M5. Although the consensus modules of M1 and M5 did not directly include the two endoglucanase families GH8 and GH48, associations between M1 and GH8, and between M5 and GH48 existed (probability values ≥ 0.005 in the respective topic probability distributions). **(B)** Genes from the 32-kb *xyl-doc* gene cluster (Ccel_1229 to Ccel_1242) encode functionalities for hemicellulose degradation. The cluster includes multiple protein families of the PDMs M1, M2, and M5, which together cover most of the cluster. Some additional protein families originate from M3 and M4 (purple). We assumed the following correspondences: CE1 ~ PF00756 (esterase); CBM22 ~ PF02018, and COG3533 (an uncharacterized protein in bacteria) ~ PF07944 (a putative glycosyl hydrolase of unknown function, DUF1680). The *xyl-doc* cluster contains a xylosidase/arabinofuranosidase gene (Ccel_1233), which is characterized as a putative β-xylosidase in the Integrated Microbial Genomes database (IMG). The gene corresponds to β*-*xylosidase genes in *Caldicellulosiruptor saccharolyticus* (Csac_2411), *Bacteroides cellulosilyticus* (BACCELL_02584 and BACCELL_00858), and *Fibrobacter succinogenes* (FSU_2269/ Fisuc_1769). Clusters containing M1 protein families were also detected around these genes.

M2 (F-measure 94.1%) contained families that bind and degrade xylan, xyloglucan, and β-glucan (Table 2), such as GH30 (β-xylosidases), GH16 (β-glucanases, xyloglucanases) [9], CBM61 (which is often found with GH16), and the fucose-binding module CBM47. In addition, M2 included the xylan-binding domains CBM6, CBM35, and PF02018, which were also present in hemicellulolytic gene clusters with M2 families of *Clostridium cellulolyticum* (Figure 3B) and *F. succinogenes* (see Additional file 6: Figure S1). In *Streptomyces lividans,* several small gene clusters of two or three genes with M2 member families might be linked to a xylan-binding mechanism involving CBM13 (also known as the ricin superfamily or R-type lectins) [56]. CBM13 and two ricin-type β-trefoil lectin domains (PF14200 and PF00652 in Table 3) belonged to M2 and occurred in the clusters. Interestingly, the two different functional aspects of M2 (xyloglucan degradation and xylan binding) were reflected by a split of the M2 module into two modules in some LDA runs.

M3 (F-measure 89.6%) included cellulose-degrading, hemicellulose-degrading, and multiple pectinolytic enzymes, such as pectin methyl esterase (CE8), pectin lyases PL1, PL9, and PF12708 (PL3), and endopolygalacturonase (GH28) (Table 2). M3 also included GH106 (α-L-rhamnosidase), which catalyzes the release of L-rhamnose from pectin (rhamnogalacturonan) molecules, and GH105, an unsaturated rhamnogalacturonyl hydrolase. Moreover, three acetyl xylan esterases (CE6, CE7, and CE12) were assigned to M3, along with the uncharacterized domain PF03629 (DUF303), which may be an acetyl xylan esterase-related enzyme (InterPro accession: IPR005181). As CE12 has both acetyl xylan esterase (EC 3.1.1.72) and pectin acetylesterase (EC 3.1.1.-) activities assigned in CAZy, the other esterase families are possibly also relevant for pectin degradation. Overall, the presence of multiple families involved in cellulose, hemicellulose, and pectin degradation confirmed the relevance of M3 for plant biomass degradation.

Module M4 (F-measure 82.5%) contained the GH5, GH43, GH2, and GH3 families, as well as some associated Pfam domains, such as a GH2 sugar-binding domain (PF02837) and the *C*- and *N*-terminal domains of GH3 (PF01915, PF00933). M4 also included GH35 and GH42, which are both β-galactosidases, and three members of a superfamily of α-galactosidases. D-galactose is an abundant component of the side chains of pectin, heteromannan, and xyloglucan [7]. Activities in the degradation of pectins have been described for several β-galactosidases from plants [58]. Furthermore, M4 seemed to be linked to xyloglucan degradation in *Bacteroides cellulosilyticus* and *Cellvibrio japonicus* (see Additional file 2: Section 4). In conclusion, M4 comprised functionally diverse glycan degradation families, in line with the heterogeneous nature of hemicellulose polysaccharides and their widely varying constituent sugars [7].

**Figure 4:**
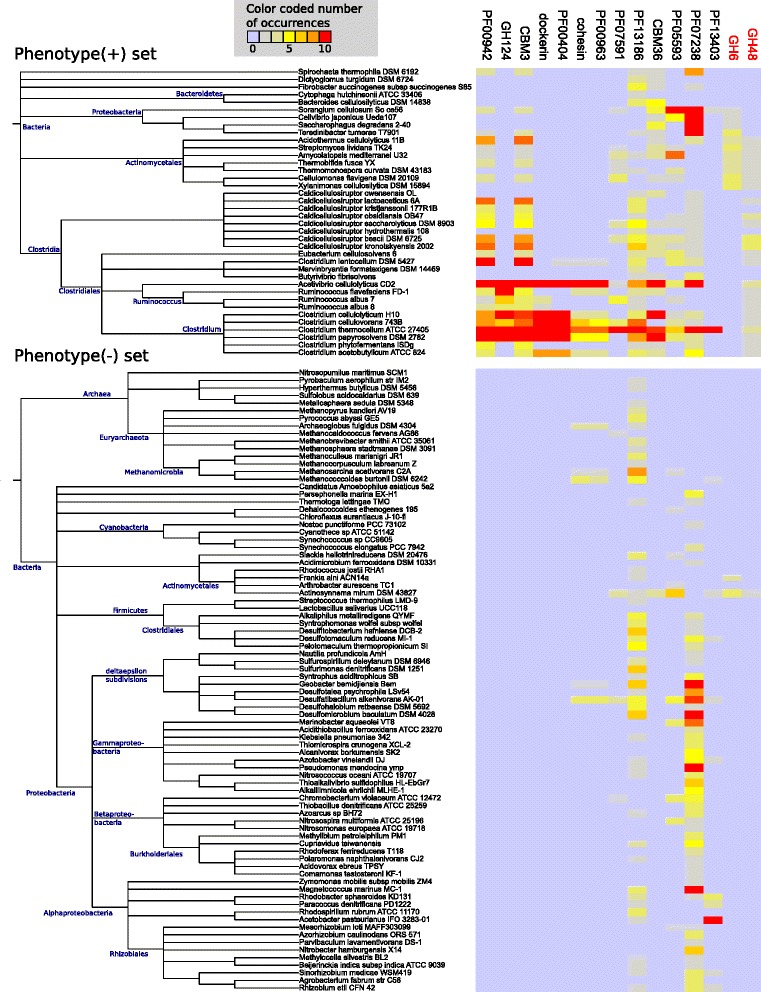
**Co-occurrences of the M5 protein families with GH6 and GH48 across known degraders and non-degraders.** Twoheat maps display the combined co-occurrence profiles for the M5 protein families and two additional cellulases, GH6 and GH48, across the known sets of the phenotype-positive and phenotype-negative genomes, respectively. GH6 and GH48 were not assigned into the plant biomass degradation module M5; in the case of GH48, this was only because of our strict cutoff criteria. However, GH48 was weakly associated with M5, and belonged to the top 50 families of the majority of M5 modules that were used to construct the consensus module. The colors of the heat map cells represent the number of instances of each family in the respective genomes of the organisms (see legends and note that the counted number of instances was limited to a maximum of 10 per genome, as described in Methods). The phylogenetic relationships of the genomes are indicated by dendrograms alongside the rows of the heat maps.

M5 (F-measure 82.1%) included structural components of the cellulosome complex (cohesin and dockerin domains), the endoglucanase family GH124, and CBMs targeting cellulose (CBM3) and hemicellulose (CBM36). CBM3 is frequently found as a domain of cellulosome scaffoldin proteins [14]. The S-layer homology domain (PF00395), which anchors cellulosomes to the bacterial cell surface [14], was not associated with M5. It was consistently grouped into modules without significant scores in our rankings, indicating that the S-layer homology domain could perform other functions in non-degraders. M5 included five more Pfam domains of unknown relevance which are interesting candidates for novel functional activities (Table 3). PF13186, a domain of unknown function in our dataset, was annotated for the gene Cthe_3076 in *Clostridium thermocellum,* which lies directly upstream of a gene cluster (Cthe_3077*–*3080) that is responsible for the structural organization of the cellulosome [59]. However, PF13186 was also annotated for non-degrading genomes (Figure 4), and has been characterized as an iron-sulfur cluster-binding domain in a recently updated version of the Pfam database.

For the complete protein family sets of the consensus PDMs, see Additional file 1 (Tables S1A-5A), which also lists PDM families that were found in fewer than 9 similar modules of the 18 LDA runs, and were thus not included in the consensus PDMs (Tables S1B-5B).

### Absence of the cellulase families GH6 and GH48

Interestingly, none of the PDMs contained the cellulase families GH6 or GH48. Both of these play an important role in cellulose degradation in some bacteria, but are not universally found in known lignocellulose degraders. They were not identified in *F. succinogenes*, *C. hutchinsonii*, or several gut and rumen metagenomes with lignocellulose-degrading capabilities [17,20,24,60]. In line with these findings, we found GH6 and GH48 to be annotated in less than 5% of the samples of our input collection, and there was only a single GH6 annotation (no GH48) in the metagenome bins. This rarity in our dataset caused weak co-occurrence signals, and is probably the cause why neither of these families were assigned to the stable, high-ranking modules (see Additional file 2: Section 5; see Additional file 7: Figures S1 and S2 (heat maps visualizing the co-occurrences of GH6 and GH48 with the protein families of M1)).

Despite this, GH48 was among the top 50 protein families of 10 functional modules used to derive the M5 consensus module. This association with M5 is in line with the fact that many bacterial cellulosomes include proteins from the GH48 family [61]. However, the probabilities for GH48 were less than the threshold value *C* = 0.01 that we required for inclusion into modules. This is also evident from a weaker co-occurrence of GH48 with the M5 protein families in lignocellulose degraders (Figure 4). Similar to GH48, most of the other members of the top 50 protein families of the M5 topics co-occurred in the phenotype-positive genomes with cohesin and dockerin annotations (see Additional file 8: Figures S1 and S2 (heat maps)). This could be indicative of functional links with cellulosomes, as for GH48. However, the weak associations of these families with M5 suggest that they are not exclusively related to the cellulosome-based paradigm. Nevertheless, their potential relevance for plant biomass degradation was indicated when we applied a less stringent cutoff to the topic distributions. With *C* = 0.005, the size of the M5 consensus module increased from 13 to 34 protein families, including GH48. Despite the substantial increase in the number of families, the F-score (80%) for the M5 module decreased by only 2% (see Additional file 9: Table S1), indicating that the additional families were relevant for the distinction between degrading and non-degrading species. Thus, even the weakly associated families seem to be predictive for plant biomass degradation. We decided to use the stringent cutoff value *C* = 0.01*,* as in our previous study, to infer smaller functional modules that could be more easily interpreted in terms of the functional contexts that they represented.

Another family with rare occurrences in the input set was GH44 (endoglucanases and xyloglucanases [62]), which appeared in less than 2% of our data samples, and was not grouped into any module. This family does not seem to be essential for all lignocellulose degraders, as its catalytic activities are also covered by the CAZy families GH5, GH9, and GH16 (Table 2) [11]. Overall, the observed rarity of GH6, GH44, and GH48 might indicate that they are non-universal across lignocellulose-degrading species. However, it might also be possible that more remote homologs exist that were not identified with the current Pfam and CAZy models.

### PDMs mapping to known gene clusters of essential lignocellulose degradation genes

The gene clusters in known degrader genomes that were identified based on the protein families of the individual PDMs included well-characterized clusters of lignocellulolytic genes. For example, the modules M1 and M5 mapped to the *cip-cel* operon and the *xyl-doc* gene cluster in *C. cellulolyticum* H10 (Figure 3). *Cip-cel* encodes genes that are essential for cellulose degradation; *xyl-doc* encodes hemicellulose degradation genes [57]. The genes from both clusters have a multi-domain architecture with catalytic and carbohydrate-binding domains [57]. Within M1, GH5, GH9, and CBM4 occurred in *cip-cel,* while CBM6, CBM35, GH10, GH43, PF00756, and PF02018 have been annotated for *xyl-doc*. Genes from both clusters also included the cohesin and dockerin domains, which reflects the cellulosome-based degradation paradigm used by *C. cellulolyticum* H10.

Interestingly, LDA assigned the cohesin and dockerin domains to the M5 module, despite their co-occurrence with the M1 families in *cip-cel* and *xyl-doc*. This is probably due to the existence of M1 families in the genomes of organisms that do not have cellulosomes, such as *Thermobifida fusca,* which is a model organism for the free enzyme paradigm (see Additional file 2: Section 6). M1 also mapped to a hemicellulolytic gene cluster in *F. succinogenes* [63,64]*,* an organism without cellulosomes that uses an unknown degradation strategy (see Additional file 6: Figure S1). Despite the evidence for a link between M5 and the cellulosome strategy, none of the PDMs proved to be exclusive for a particular degradation paradigm (Figure 2). As described above, the M5 module also contained five Pfam families whose functional descriptions have no known link to lignocellulose degradation (Table 3). These five Pfam families shared co-occurrence patterns with the cohesin and dockerin domains, but they also occurred in organisms using free cellulolytic enzymes, such as some *Caldicellulosiruptor* species (Figure 4). Thus, M5 also covered non-cellulosome-related functionalities (see Additional file 2: Section 7).

### Predicting the ability for plant biomass degradation

We predicted the presence of PDMs for the 3,096 remaining genomes and taxonomic metagenome bins if their completeness was greater than or equal to the threshold determined for each PDM (see Methods; see weight thresholds in Table 4). Overall, the presence of one or more PDMs was predicted for 8.4% (28/332) of the taxonomic bins and 24.7% (683/2,764) of the genomes (see Additional file 10: Tables S1-5). Most genomes and bins to which M1 was assigned also had M2 assigned to them (82% of 132 M1 assignments occurred jointly with M2 assignments). This agreed with the cellulose/hemicellulose-degrading (M1) and hemicellulose-targeting (M2) enzymatic activities we determined for these modules, which are both essential for lignocellulose degradation [44]. The majority (52.5%) of all predictions was exclusive to M4 (see Additional file 11: Venn diagram). As M4 included functionally diverse glycan degradation families, and had the lowest precision (82.1%) of all modules for lignocellulose degraders, these assignments probably reflect a general ability of the respective organisms to degrade carbohydrate substrates of plant origin.

In a previous study [44], Medie *et al.* analyzed the distributions of CAZy families representing cellulases, hemicellulases, and pectinases across approximately 1,500 complete bacterial genomes. The authors classified almost 20% of these organisms as saprophytic bacteria, based on the presence of at least one cellulase and three or more hemicellulases or pectinases. Saprophytes feed on dead organic matter of plant origin, and thus are likely to include lignocellulose-degrading species. Based on the same CAZy families and criteria as described by Medie *et al*. [44], we determined potential saprophytes in our dataset (see Methods). In total, about a quarter (27.2%) of all 3,216 genomes and metagenome bins fulfilled these criteria. The number of predicted saprophytes thus further supports the notion that the ability to degrade plant biomass is a common trait in Bacteria and Archaea species. The genomes and metagenome bins with predicted PDM occurrences were clearly enriched with potential saprophytes (75% of all predictions). This enrichment was particularly large for M1 (99%), M2 (91%), and M3 (100%).

**Figure 5:**
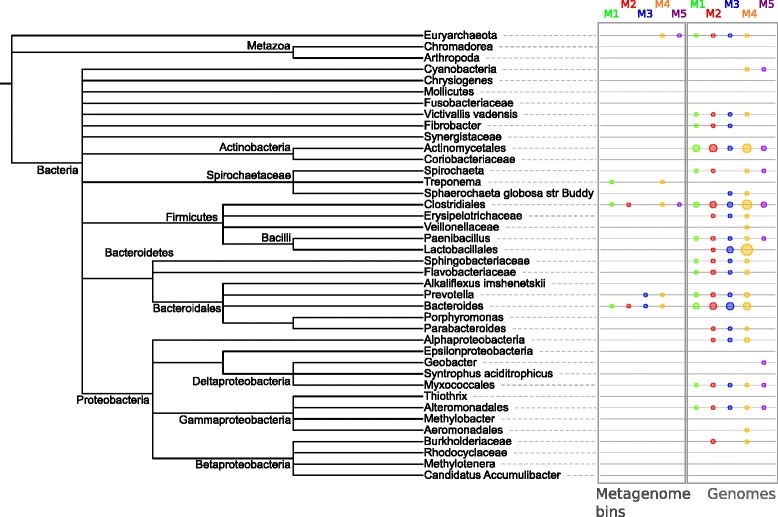
**Comparison of plant biomass degradation module (PDM) occurrences in metagenome bins and isolate genomes of corresponding taxa.** The colored circles at the leaf nodes of the tree denote the predicted occurrences of the different PDMs in the respective taxa or their subclades. The tree was constructed from the taxonomic assignments of the metagenome bins in our input set (see Methods). We then mapped the predicted occurrences of the PDMs in 28 different metagenome bins to the leaf nodes of the tree. If PDMs were identified in two or more bins, with one being a parental taxon to the other, the parent taxon is displayed. For example, predictions for *Prevotella ruminicola* were summarized with other predictions for the genus *Prevotella.* In addition, PDM occurrences in 403 isolate genomes of corresponding taxa were also mapped to the leaf nodes. The area of the colored circles for the isolate genomes was sized proportionally to the number of genomes for which the respective PDM was identified. The PDM predictions for the metagenome bins covered only five major taxonomic clades. The majority of PDMs in the metagenome bins were assigned to the orders Bacteroidales and Clostridiales. For the genomes, PDMs were identified from a broader range of taxa, including Actinobacteria, Firmicutes, Bacteroidetes, and Proteobacteria, in agreement with the estimated taxonomic range of potential cellulose-degrading species reported in [45]. The differences in the taxonomic distributions of the identified PDMs between genomes and metagenome bins may partly reflect the abundance of Bacteroidales and Clostridiales degraders in plant biomass-degrading communities, but it is also likely that some PDMs were not identified in the metagenome bins because of the partial nature of the genomic information recovered.

The metagenome bins that were assigned PDMs came from cow rumen, reindeer rumen, manatee gut, Tammar wallaby gut, and termite hindgut samples, and samples of a methylotrophic and a terephthalate-degrading community. Most of these communities, except for the methylotrophic and terephthalate-degrading ones, are known to include lignocellulose-degrading community members; however, their taxonomic affiliations are only partly known [19,65,66]. The coverage and quality of the protein-coding sequences was heterogeneous across the 332 bins that we analyzed: 63 bins could only be annotated with fewer than 10 protein families, while the remaining bins were annotated with 276 different protein families on average. It is well known that the gene content of metagenome bins is often incomplete, particularly for community members of low abundance, which is caused by insufficient sequencing depth or insufficient DNA read lengths [67]. Overall, the PDMs were predicted to be present in 28 bins covering 5 major taxonomic clades (Figure 5). PDMs occurring in metagenome bins of Bacteroides, Prevotella, and Lachnospiraceae (Clostridiales) were in line with the taxonomic affiliations of cellulose degraders found in mammalian gut and rumen microbial communities [68]. Furthermore, the PDMs accurately identified Bacteroidales and Treponema bins that have been shown to be involved in lignocellulose degradation in recent metagenome studies of cow rumen [69], and termite hindgut [60], thus indicating the benefit of our method to guide the discovery of uncultured microbial taxa with lignocellulolytic activities. Our results also indicated two archaeal extremophile species that have plant biomass degradation capabilities (see Additional file 2: Section 8).

### Identification of gene clusters and polysaccharide utilization loci in the predicted (meta-)genomes

To identify new candidate clusters of genes encoding the ability to degrade lignocellulosic plant biomass, we searched for gene clusters encoding PDM protein families in the 711 genomes and taxonomic bins with assigned PDMs, using the same criterion as above. We found 379 gene clusters of 4 or more genes for individual PDMs, which mapped to 342 distinct gene clusters in 168 genomes and 6 taxonomic bins. Genome fragmentation caused by incomplete assembly of bacterial draft genomes from IMG and taxonomic bins in our dataset may have decreased the number of detected clusters. The average distance between the genes was 369 bp, which was almost the same as the average intra-cluster gene distance observed for the detected clusters in the phenotype-positive organisms. Most of the gene clusters occurred in Bacteroidetes (54.3%); 22.4% and 12.7% occurred in Firmicutes and Actinobacteria, respectively. The first two phyla are predominant in gut and rumen environmental communities with lignocellulose-degrading abilities [68,70].

Some of the newly identified gene clusters may cover polysaccharide utilization loci (PULs) targeting various kinds of polysaccharides. We found gene clusters in 39 isolate Bacteroides species, which are generally known to possess PULs [65]. As an example, the pectin-related PDM M3 identified gene clusters in *B. thetaiotaomicron* that represent parts of two regions that have been shown to be active in rhamnogalacturonan degradation in a PUL-targeted study [71]. Moreover, LDA inferred a stable functional module related to PULs, which included a suite of outer membrane proteins as well as the two core proteins that are known to define PULs, namely SusD- and SusC(TonB)-like membrane proteins (see Additional file 1: Table S6A). This module was not one of the high-ranking modules, which can be explained by the broad substrate specificity of PULs for various polysaccharides, including starch in particular [15,65]. While analyzing gene clusters of PDM protein families, we found hybrid gene clusters linking the PUL module to the glycoside hydrolases of the PDMs M1 and M2. For example, we identified gene clusters corresponding to previously characterized Sus-like PULs from *Bacteroides ovatus* targeting xyloglucan and xylan [71] (see Additional file 2: Section 9).

### Predicting the ability for plant biomass degradation in a cow rumen microbial community

Hess *et al.* [19] reconstructed 15 draft genomes from the metagenome of a switchgrass-degrading microbial community from a cow rumen. In a previous study (Weimann *et al*. [28]), we have cross-linked the data from cellulolytic enzyme screens of the study by Hess *et al.* with protein family annotations of the 15 draft genomes to identify potential plant biomass degraders from the cow rumen metagenome. Strikingly, all 4 families (GH5, GH9, GH10, and GH26), which have been described by Weimann *et al.* to correspond to the (hemi-)cellulolytic enzymes of the cow rumen bins with degradation abilities confirmed by activity screens (see Table 3 in Weimann *et al*. [28]), were grouped together in the PDM M1 in our study.

In the current study, we investigated whether PDM assignments allowed identification of the plant biomass-degrading community members in the cow rumen metagenome (Table 5). The presence of M1 or M2 identified all degraders, in agreement with the enzyme screen results and our previous assignments with a family-centric SVM classifier [28]. M1 was also present in the draft genome APb, for which no lignocellulolytic enzymes were confirmed, but which is closely related to a known plant biomass-degrading species (*Butyrivibrio fibrisolvens*). The PDMs mapped to six gene clusters with four or more genes and several shorter clusters in the draft genomes. We investigated these, and found an interesting cluster in the Bacteroidales-associated draft genome AGa, containing genes annotated with GH5, GH94, and two unannotated gene sequences (see Additional file 2: Section 10; see Additional file 12: Figure S1; see protein sequences in Additional file 13).

**Table 5:** **Module based identification of potential biomass-degrading cow rumen draft genomes**

**Draft genome**	**Taxonomic affiliation**	**M1**	**M2**	**M3**	**M4**	**M5**
AJ^a,b^	Bacteroidales	+	+	+	+	
AGa^a,b^	Bacteroidales	+				
AC2a^a,b^	Bacteroidales		+			
AIa^a,b^	Clostridiales	+			+	
APb^b,c^	Clostridiales	+			+	
AH^b^	Bacteroidales	+			+	
AFa	Spirochaetales				+	
AN	Clostridiales				+	
AWa	Clostridiales				+	
ATa	Clostridiales					+
ADa	Myxococcales					
AMa	Spirochaetales					
AQ	Bacteroidales					
AS1a	Clostridiales					
BOa	Clostridiales					

^a^AJ, AGa, AC2a and AIa are supported by evidence for lignocellulolytic activity according to carbohydrolytic activity tests [19].

^b^The draft genomes AJ, AGa, AC2a, AIa, APb and AH were also predicted by an SVM-based method for predicting lignocellulose degraders (counting only the unambiguous predictions of the SVM classifier) [28].

^c^APb was mapped using 16S rRNA marker genes to the known lignocellulose-degrading organism *B. fibrisolvens* [19].

We used the weights of the consensus plant biomass degradation modules (PDMs) in the 15 draft genomes of the cow rumen metagenome to predict the draft genomes with lignocellulolytic activities (indicated by + signs), using the weight thresholds reported in Table 4.

## Conclusions

Degradation of lignocellulosic plant biomass is a complex biological process with a number of mechanisms across different microbial species, which are currently only partially understood. In this paper, we describe functional modules of protein families linked to plant biomass degradation, which we identified based on co-occurrence patterns and partial phenotype information. Using LDA, a state of the art Bayesian inference method, we inferred 400 potential modules from a set of 2,884 genomes and 332 taxonomic bins from 18 metagenomes. Such modules represent sets of functionally coupled protein families, and cover a broad range of biochemical processes, as shown previously [42]. We then determined the presence of modules in genomes of known lignocellulose-degrading species and non-degraders to calculate a ranking of the modules that reflected the strength of their association with the plant biomass degradation phenotype. We analyzed the stability of the top-ranking modules across several executions of the LDA method, and determined five consensus functional modules (PDMs) involved in plant biomass degradation.

For the ranking of the modules, we used the learning set from our previous study [28], in which we had linked individual protein families to plant biomass degradation, and extended it by 20 additional phenotype-positive organisms. Despite of these additions, the number of confirmed degrader species is still small, compared with the estimated abundance of potential plant biomass-degrading species reported in two other studies [44,45]. Based on these estimates, 20 to 25% of bacterial genomes could possess plant biomass degradation capabilities. The unsupervised topic model of LDA allowed us to also include genomes and taxonomic metagenome bins lacking phenotype information in the inference process. The modules could thus be inferred from the known phenotype-positive genomes as well as from currently unknown degraders and cellulolytic communities in the dataset. To our knowledge, this is the first study to globally analyze the available genome sequence and phenotype data to determine the functional modules of protein families that are linked to plant biomass degradation.

The PDMs included many protein families known to be involved in the degradation of cellulose, xylan, xyloglucan, and pectins, which are the main components of plant cell walls, with families that target the same macromolecules being grouped together. Overall, the PDMs contained 87 CAZy and Pfam families. We discussed 41 of these in detail in the functional contexts of the PDMs in which they were placed. Nineteen CAZy families were also represented by additional isofunctional Pfam families in the PDMs. These two sets account for 60 of the 87 families. Of the 27 remaining families, 7 were carbohydrate-active families, such as GH55, GH88, GH95, or alpha-amylase, which are involved in the degradation of polysaccharides. The remaining 20 families are listed in Table 3. Their functions were less clear, and they represent candidate families with potential roles in plant biomass degradation. Even more potentially interesting families were found in the high-ranking modules, but were not included in the consensus modules because they occurred in less than half of the modules used to construct the consensus. Some of these families might be interesting for further investigation.

The functional coherence of PDM member families was also supported by their localization in gene clusters in lignocellulolytic microbes. These included several known clusters of lignocellulolytic enzymes, such as *cip-cel* and *xyl-doc* from *C. cellulolyticum* H10. Based on the modules, we identified overall more than 400 gene clusters in different organisms of our dataset, which could potentially be linked to the degradation processes. These clusters may include PULs targeting different kinds of polysaccharides. We discussed some examples of identified PULs and Sus-like PULs that have been described as targeting rhamnogalacturonan, xyloglucan, and xylan in previous studies.

In addition, we investigated whether certain modules were specific to different degradation paradigms, as the module M5, for example, contained cellulosome-related families, such as cohesin, dockerin, and CBM3. We found that none of the modules was exclusive to a specific degradation strategy, and the modules instead spanned different paradigms. We believe that the granularity of the modules could be further improved in future if more and better curated phenotype information becomes available, which would allow us to enrich the set of genomes with species having different confirmed paradigms. For instance, the identification of genes from Sus-like cellulose-interacting protein complexes, as reported by Pope and Mackenzie [65], and Naas *et al*. [69], would probably require more accurate profile hidden Markov models (HMMs) for *susD*-like genes. For these, the sequences of relevant genes in more organisms that use the Sus-like paradigm would need to be known. Within our learning set, only *B. cellulosilyticus* uses a Sus-like strategy on hemicellulosic polysaccharides [72].

The PDMs allowed us to predict the ability of lignocellulose degradation with cross-validation accuracies of up to 96.7%, which we used to predict the ability to degrade plant biomass for all genomes and taxonomic bins with unknown degradation status in our dataset. The predicted degraders were clearly enriched with organisms that were likely to have a saprophytic lifestyle. For 15 draft genomes of a microbial community from a cow rumen, we confirmed the predictions by cross-linking to enzymes with demonstrated lignocellulolytic activities. In addition, the PDMs identified metagenome bins with cellulolytic capabilities for several microbial communities.

The PDMs contained many of the protein families that we had previously identified with a family-centric approach in a smaller set of 19 known lignocellulose degraders and 3 metagenomes, including CBM3, CBM4, CBM_4_9, CBM6, GH5, GH10, GH26, GH43, GH55, GH88, and GH95 [28]. Nevertheless, differences in the results existed. For example, in our previous study [28], only a few pectin-related families were identified (PL1, GH88, and GH106), but in the current study we identified an entire PDM (M3) of pectin-degrading families, which included these three families together with PL3, PL9, GH28, GH105, CE12, and additional related ones. Differences were also found for individual families. For example, the PDMs were linked to GH9, GH48, cohesin, and dockerin, as well as to elements of xylan binding, such as the CBM13 and lectin domains, which were not identified with the family-centric approach. However, GH6 and GH44 were not associated with the PDMs.

The families GH6, GH44 and GH48 occurred in less than 5% of the input genomes and metagenome bins, and their co-occurrence patterns with other families were more subtle in our large data collection than in the smaller dataset analyzed previously. These observations, which are in agreement with previous reports about the absence of GH6 and GH48 in the genomes and metagenomes of known lignocellulose-degrading species and microbial communities [17,20,24,60], suggest that GH6, GH44, and GH48 are not universally present in lignocellulose-degrading bacteria. However, we cannot exclude the possibility that remotely related family members that perform the functions of GH6, GH44 or GH48 are encoded in these genomes, which were not detected by the currently available family-specific HMM profile models. This could be further investigated by experimental screening for these enzymatic activities and identification of the respective proteins from the taxa that seem to lack these families.

In addition to differences in dataset sizes and composition, methodological differences between the family-centric and the PDM-based approach are likely to be responsible for the differences observed and the additional relevant families that were included in the PDMs. Neither approach identified any gene families related to lignin degradation. This may be because lignin-related protein domains, except for the broadly defined peroxidase family PF00141, were largely missing from the Pfam and CAZy/dbCAN databases. Furthermore, reports of lignin decomposition have been dominated by fungi [73], and thus the corresponding mechanisms might have been under-represented in our bacterial and archaeal dataset.

We found evidence for functional links of the protein families in the PDMs with each other and the plant biomass degradation phenotype, which includes the co-occurrences of these families across genomes, co-occurrences with known relevant families, clustering within the genomes of known degraders, and the predictive value of the PDMs for identifying plant biomass degraders. Given this extensive support, an experimental characterization of the protein families with unknown relevance for plant biomass degradation in the PDMs, and their respective gene clusters, is likely to reveal new biochemical functionalities for plant biomass degradation. With the method we have described, other phenotypes, such as, nitrogen fixation or antibiotic resistance, could be studied from existing genome datasets in a similar fashion.

## Methods

### Latent Dirichlet allocation

LDA is a text-mining method for extracting semantic concepts (that is, topics) from a collection of text documents [41]. The topics reflect groups of semantically related words supported by co-occurrence signals across the document collection. LDA is a generative probabilistic model assuming a well-defined process as the source of the observed documents. With Bayesian inference and MCMC methods such as Gibbs sampling, the generative process can be reversed [74,75], which corresponds to increasing the probability of the model by fitting latent variables to make the outcome of the process match the observed documents as closely as possible. Here, we are interested in inferring the latent variables, not in the outcome of the process itself.

The input for LDA is a collection of *N* documents, where each document is a collection of words stemming from a controlled vocabulary *V*. The order of words in the document is not important (termed the “bag of words” assumption). LDA assumes the existence of *T* latent topics, and each topic is represented as a discrete multinomial distribution over *V*.

One variable of the model with central meaning is the vector $\overrightarrow{z}$, which contains a random variable *z* for each word of the text collection that models the latent origin of the word with respect to the *T* topics. According to the model, the probability of observing word *w* in document *d* of the collection is given by:

$$ P\left(w\Big|d\right)={\displaystyle \sum_{t=1}^T\underset{\varphi_t(w)}{\underbrace{P\left(w\Big|z=t\right)}}\cdot \underset{\theta_d(t)}{\underbrace{P\left(t\Big|d\right)}}}, $$

where *φ*_*t*_(*w*) defines the multinomial distribution representing topic *t*, and *θ*_*d*_(*t*) corresponds to a multinomial distribution describing the document-specific prior probabilities of the topics. The parameters $\overrightarrow{z}$, *φ*_*t*_(*w*), and *θ*_*d*_(*t*) for all documents and topics are latent variables of the hidden generative process, which can be estimated efficiently with MCMC sampling methods.

### Genome and metagenome annotation

Protein sequences for bacterial and archaeal species were downloaded from IMG (version 3.4) and metagenomic protein sequences were obtained from IMG with microbiome samples (IMG/M, V3.3). In addition, we collected samples of microbial communities from Svalbard reindeer rumen [20], termite hindgut [60], manatee gut, and Tammar wallaby forestomach [66], as well as draft genomes reconstructed from a metagenome sample of a switchgrass-degrading microbial community in a cow rumen [19]. If no protein-coding sequences were available, genes were predicted by MetaGeneMark [76]. Note that, from the metagenomes, only protein sequences with a predicted taxonomic origin were included in our dataset. For this purpose, taxonomic bins from IMG/M or the original publications, or those generated in-house were used, which were inferred with either *PhyloPythia* [77] or *PhyloPythiaS* [78] using sample-specific training sequences and taxonomic models constructed with taxa that represent the more abundant community populations. Overall, we worked with protein-coding sequences from 2,884 prokaryotic genome sequences and 332 taxonomic bins derived from 18 metagenome samples. Protein sequences were annotated with profile HMMs of protein families from the Pfam database (Pfam-A, V26.0) and CAZy families from the dbCAN database [79] using HMMER 3.0 [80]. Multiple matches of different domains per protein were allowed. All matches were required to satisfy an e-value of 1e-02 and a bit score of 25 or more. For Pfam, the family-specific thresholds from the Pfam database (gathering thresholds) were used if they were stricter than our default threshold. Such family-specific thresholds were not available from dbCAN. For large families (more than 100 amino acids) of the dbCAN database, we used the threshold 1e-04 instead of 1e-02. We then converted the protein family annotations for the genomes and taxonomic metagenome bins into a suitable input collection for LDA (see Additional file 3: Section 1).

Homology-based annotation of protein families can generate some FP or FN annotations [81], which may affect the accuracy of the downstream analysis. Therefore, robust computational methods capable of handling potential annotation errors should be chosen to obtain reliable results. Bayesian probabilistic models such as LDA are well suited for the inference of robust associations from potentially noisy datasets [82,83].

### Functional module inference with LDA

We used the protein family collection of the (meta-)genomes as input for the LDA inference procedure to predict potential functional modules, as demonstrated previously [42]. Note that the identifiers of the protein families (e.g. 'GH28') were used to define the words of the vocabulary *V* in the LDA model. Because of the larger input collection compared with our previous work, we increased the number of topics from 200 to 400. Despite the increased number of documents (3,216 *versus* 575), there was a slight decrease in the vocabulary size (8,413 *versus* 10,431), owing to differences in coverage between the Pfam-A and eggNOG databases. As in [42], we used the parameter value *C* = 0.01 to convert topic probability distributions into discrete sets of protein families, which represented our potential functional modules. Given the vocabulary *V*, and the multinomial distribution over words of *V* for topic *t*, that is, the topic distribution *φ*_*t*_(*w*), we defined module *M*_*t*_ as *M*_*t*_ : = {*w* ∈ *V*|*φ*_*t*_(*w*) ≥ *C*}. The module *M*_*t*_ thus contained the protein family identifiers that were most strongly related to topic *t*. The families assigned to *M*_*t*_ share common co-occurrence patterns, and were therefore likely to be functionally coupled based on the “guilt by association” principle [39].

### Phenotype annotation

We assigned the lignocellulose-degrading phenotype to genomes by manually curating the annotations of “(ligno)cellulose degradation” or “(plant) biomass degradation” that we obtained from the databases of IMG, the Genomes Online Database (GOLD) [84], and the German Collection of Microorganisms and Cell Cultures (DSMZ) [85], based on information from the literature. Removal of ambiguous or inconsistent phenotype annotations resulted in 38 confirmed lignocellulose degraders (phenotype-positive genomes), which degraded some or all components of lignocellulose (see Additional file 14: Table S1). The set of phenotype-positive genomes is a superset of the 19 lignocellulose-degrading microbes (except *Postia placenta*) from our previous work [28]. We adopted the set of 82 phenotype-negative genomes from the same study, which were also manually curated using information from the literature. There was less certainty in phenotype-negative annotations, as it may be that a particular phenotype has not been discussed in the literature; however, the statistical methods we used to determine PDMs from these datasets can tolerate a certain amount of error.

### Definition of module weights

The inference of a topic model with LDA from a collection of *N* input documents results in *T* potential functional modules. We extracted 400 modules from 3,216 genomes and metagenome bins. We then applied an attribute-ranking approach to sort the modules according to their relevance for lignocellulose degradation. As attributes to be used in the ranking procedure, we defined module weights. A weight, *weight*_*t*_(*d*), should reflect how likely the module *M*_*t*_ is to be contained in the genome or metagenome bin encoded as document *d* of the input collection. Given *N* genomes or bins as input, and *T* modules, we can summarize the weights in a weight matrix *W* ∈ *ℝ*^N×T^ with entries *w*_*dt*_ : = *weight*_*t*_(*d*).

Two different definitions of weights (probability weights and completeness scores) were tested (see Additional file 3: Section 2). We decided to use completeness scores, as they produced more relevant results, though the rankings obtained with both choices of weights largely agreed (see Additional file 2: Section 11). The completeness score of a module is the percentage of the protein families of a module that occurred in a specific genome or taxonomic bin. More precisely, we defined the weight of module *M*_*t*_ in document *d* of the (meta-)genome collection based on completeness as:

$$ weigh{t}_t(d):=\frac{\left|{M}_t\cap d\right|}{\left|{M}_t\right|}\times 100\%, $$

where |*M*_*t*_ ∩ *d*| is the size of the intersection of the protein family sets of module *M*_*t*_ and document *d*, and |*M*_*t*_| is the number of protein families contained in *M*_*t*_.

### Identification of phenotype-defining functional modules

To identify phenotype-associated modules, we used the weights of the modules in the input documents that corresponded to the manually curated phenotype-positive and phenotype-negative genomes. We refer to these genomes as the “learning set.” The selected weights were used to predict the phenotypes of these genomes, and we scored each of the 400 modules according to its ability to distinguish between the two phenotype classes. More precisely, the classification of the learning set with respect to module *M*_*t*_ was carried out by applying a threshold value *γ*_*t*_ to the weights of the module, that is, genomes were predicted to be phenotype-positive if the respective weights satisfied the threshold, or phenotype–negative if they did not.

The ranking procedure optimized independent thresholds for all modules by finding the threshold that maximized a criterion function. We used the F-measure [86] with the parameter *β* = 0.5 for scoring (recall half as important as precision [54]; see Additional file 3: Section 3), which can be computed using the confusion matrix shown in Table 6. Finally, we obtained the ranking of the modules by sorting them in decreasing order, based on their F-measure scores.

**Table 6:** **Confusion matrix**

***M***_***t***_	**Document** ***d*** **is phenotype-positive**	**Document** ***d*** **is phenotype-negative**
*weight*_*t*_(*d*) ≥ *γ*_*t*_	TP	FP
*weight*_*t*_(*d*) < *γ*_*t*_	FN	TN

FN, false negative; FP, false positive; TN, true negative; TP, true positive.

### Mapping of modules between Gibbs samples and runs

Finding the optimal assignments of protein families to functional modules, such that the observed data can be explained in the best possible way, is a combinatorially challenging task. We used Gibbs sampling to derive statistical estimates for the latent topic distributions of the LDA model, from which we derived the potential functional modules as described. We then searched for similar modules across several LDA runs to identify stable modules, because with the MCMC inference technique used, there is variance in the derived estimates across different runs. We used the Kullback–Leibler divergence [42] and the Jaccard distance [87] to calculate pairwise distances between topics (probability distributions) or modules (discrete protein family sets), respectively. As expected, we observed good agreement between the results with both distance measures. Given the matrix of pairwise distances for the modules of two LDA runs, we used the Hungarian algorithm [88] to find an optimal global mapping between these. The Bron–Kerbosch algorithm [89] was used to find cliques of similar modules efficiently across multiple LDA runs (see Additional file 3: Section 4).

### Consensus modules

In theory, Gibbs sampling efficiently estimates the posterior distribution of the model parameters and converges to a global optimum given a sufficient number of iterations [46]. However, in practice, there is variance in the results of individual LDA runs, and a common approach to derive a stable solution is to repeat the inference multiple times and to compare the results from a number of runs [74]. Therefore, we repeated the steps of our analysis several times with the same input data. In comparison with our previous study [42], we doubled the number of LDA runs to 18. In each run, we inferred 400 potential functional modules. As described in the previous section, we tracked the identities of the modules across all runs based on pairwise module distances, and thus characterized the stability of the modules. Next, we applied the described attribute-ranking scheme based on the completeness scores to each of the 18 sets of 400 inferred modules, and determined the top 15 modules for each run. Among these highly ranked modules from different runs, we searched for similar modules that occurred in at least 75% of the 18 runs. From these recurring modules, we derived consensus modules of protein families (see Additional file 1: Tables S1A-5A) as follows. Given a set of similar modules from different LDA runs, which were identified as representing a stable module across 14 runs or more, the corresponding consensus module contained all protein families that occurred in at least 9 modules of this set.

### LOO analysis and 10-fold cross-validation

For the consensus PDMs, we performed LOO and 10-fold cross-validation experiments to assess their predictive accuracy. In a loop, we successively left out each individual genome (or 10% of the genomes) of the learning set, and optimized the weight threshold of a module on the remaining learning set with the F-measure. For the omitted genomes, the PDM was predicted to be present if the genome-specific module weight was greater than or equal to the inferred threshold. In both settings, we obtained exactly one prediction for each genome of the learning set, based on which we calculated performance measures, such as precision and recall, the F-measure, the cross-validation accuracy, and the cross-validation macro-accuracy. For the 10-fold cross-validation experiments, we randomly split the data to create the different folds. The procedure was repeated 10 times, and the results were averaged. For a more accurate estimate of the test error, we also calculated 95% confidence intervals for the cross-validation accuracies of the modules. We used the Clopper–Pearson bound [53], which is an estimate based on the binomial distribution and the observed error rate on the omitted test samples. Note that the number of available test samples (120 in our case) is an important parameter of the binomial, and determines the sizes of the intervals. With a larger set, narrower bounds would be obtained.

### Prediction of module occurrences in genomes and metagenome bins

We optimized the cutoff thresholds *γ*_t_ for module prediction by maximizing the F-measure using the weights of the consensus modules for all genomes with a known phenotype (that is, the genomes of our learning set). We then considered the module weights in the genomes and metagenome bins of unknown phenotype to predict occurrences of the modules. We applied the following prediction rule to predict the presence of a module *M*_*t*_ in the genome or metagenome bin that corresponds to document *d* in the input of LDA:

$$ predict\;\left(d,{M}_t,{\gamma}_t\right)=\left\{\begin{array}{l}1\kern1em  if\kern0.5em  weigh{t}_t(d)\kern0.62em \ge {\gamma}_t\\ {}0\kern1em  otherwise\end{array}\right. $$

### Comparison of PDM occurrences in the taxonomic bins of metagenomes and isolate genomes of the corresponding clades

We constructed a tree based on the NCBI taxonomy tree with the tool iTOL [90] for the taxa represented by the metagenome bins in the dataset. Metagenome bins with fewer than 10 protein families were excluded from consideration. We used the taxonomic assignments inferred by the binning methods *PhyloPythia* [77] and *PhyloPythiaS* [78]*,* except for high-ranking bins, such as bacteria. To visualize the common occurrences of the PDMs at the leaf nodes of the tree, we collapsed some of the original leaf nodes to new leaf nodes of higher ranks. This was carried out if two or more of the PDMs were predicted to occur in taxa of the same clade, but with different ranks. In these cases, the PDMs involved were displayed for the highest common observed rank. The PDMs were predicted to occur in the bins of only five major taxa across the different metagenomes (Figure 5). In addition, we also mapped isolate genomes of the corresponding taxa with predicted occurrences of the PDMs to the leaf nodes of the tree.

### Identification of saprophytic genomes and taxonomic bins

As described by Medie *et al*. [44], we classified a genome or metagenome bin as belonging to a cellulase- and hemicellulase-containing saprophyte if the corresponding annotation set contained at least one cellulase and three or more hemicellulases or pectinases from the following families.

• Cellulase families: GH5, GH6, GH8, GH9, GH12, GH44, GH45, GH48, GH74, and GH124.

• Hemicellulase and pectinase families: GH10, GH11, GH26, GH28, GH30, GH43, GH53, GH67, GH78, PL1, PL2, PL9, PL10, PL11, and PL22.

### Implementation and parameter settings

We used the LDA implementation from the topic modeling toolbox [91]. The LDA model depends on two hyperparameters, *α* and *β*, which control the Dirichlet priors of the multinomial distributions. We used the default values of the topic modeling toolbox, that is, α = 50/*T* and *β* = 0.01. We specified 2,000 iterations as the burn-in phase of a run. After burn-in, we collected 50 Gibbs samples and derived the topic distributions by averaging over the samples (see Additional file 3: Section 5).

## Additional files

Additional file 1:
**Protein families of the consensus plant biomass degradation modules (PDMs).** The Tables S1A-5A show each consensus module as a list of Pfam/CAZy terms. The consensus modules summarize highly similar modules from the 18 LDA runs and contain all elements that occurred in nine runs or more. The tables S1B-5B contain information about all additional Pfam/CAZy families that occurred in the similar modules in less than nine runs. Tables S6A and S6B list the families of the additional PUL module (see Results section). Additional file 2:
**Supplementary Note.** Additional details about the results of the main manuscript. Additional file 3:
**Supplementary Methods.** Additional details about the methods and preparation of the input data. Additional file 4:
**Plant biomass degradation module (PDM) assignments to the genomes of the learning set.** PDM assignments to the genomes of known plant biomass degraders and non-degraders are visualized in Figure S1, and were obtained by leave-one-out classification. Additional file 5:
**Single predictions of the consensus modules on the learning set of genomes.** Each sheet of the Excel file lists the predictions of one of the consensus modules with respect to the learning set of genomes (Tables S1-5). Table S6 contains the predictions of the additional PUL module. We used different colors to mark true-positive (TP), true-negative (TN), false-positive (FP) and false-negative (FN) predictions (a description of the color coding is contained in the first sheet of the file). For each classified sample, we have provided several details (for example, name and phylum), as well as the genome-specific module weight (completeness score) of the respective consensus module. Additional file 6:
**Hemicellulolytic gene cluster in** ***Fibrobacter succinogenes*** **S85.** The gene cluster in Figure S1 encodes more than 10 hemicellulose-targeting enzymes in the genome of *F. succinogenes* S85. The protein domain architecture of the cluster genes has been described by Yoshida *et al*. [63,64]. *F.* s*uccinogenes* does not use a cellulosome-based degradation strategy, but rather a degradation paradigm that is still uncharacterized [92,93]. Additional file 7:
**Co-occurrence profiles of the M1 protein families and GH6/GH48 across the learning set.** Two heat maps display the combined co-occurrence profiles of the M1 protein families and two additional cellulases, GH6 and GH48, across the sets of the known phenotype-positive (Figure S1) and phenotype-negative (Figure S2) genomes, respectively. GH6 and GH48 were not assigned to module M1. The colors of the heat map cells represent the number of instances of each family in the respective genomes of the organisms (see legends and note that the counted number of instances was limited to a maximum of 10 per genome, as described in Methods). The phylogenetic relationships of the genomes are indicated by dendrograms alongside the rows of the heat maps. Additional file 8:
**Co-occurrence profiles of protein families that were weakly associated with M5 (across the learning set).** Two heat maps displaying protein family co-occurrence profiles across the known phenotype-positive (Figure S1) and phenotype-negative (Figure S2) genomes. The columns of the heat maps represent the families that were weakly associated with the M5 module. These families did not satisfy the required threshold *C* = 0.01*,* but they belonged to the 50 protein families with the highest probabilities in the 16 topics that were used to create the M5 consensus module. Because the families failed to match the threshold, they were not counted for the consensus of M5. One example for such a family is GH48 (discussed in the main text). Families are ordered from left to right according to the number of topics in which they occurred. Families that occurred in less than 9 of the 16 topics are not displayed. We also added the cohesin and dockerin domains of M5 for a comparison. The colors of the heat map cells encode the number of instances of each family in the respective genomes of the organisms. Additional file 9:
**Leave-one-out results for the consensus plant biomass degradation modules (PDMs) obtained with the threshold** ***C*** **= 0.005.** The threshold *C* = 0.01 was used to convert the discrete topic probability distributions of the LDA model into potential functional modules (see Methods). In additional tests, we used the threshold *C* = 0.005 instead. This cutoff level is less strict and allowed families with smaller probabilities to be included in the potential functional modules. This also resulted in enlarged consensus modules for the PDMs. Table S1 summarizes the results obtained in leave-one-out validation for the PDMs M1 to M5 based on the threshold *C* = 0.005. Additional file 10:
**Single predictions of the consensus modules on the remaining set of genomes and metagenome bins.** Each sheet of the Excel file lists the predictions of one of the consensus modules with respect to all genomes and metagenome bins, except for the 120 known (non-)degraders used for learning (Tables S1-5). Table S6 contains the predictions of the additional PUL module. For each classified sample, we provide several details (for example, name and phylum), as well as the genome-specific module weight (completeness score) of the respective consensus module. Additional file 11:
**Venn diagram of the predicted occurrences of the modules M1 to M4.** The diagram displays the overlap between the genomes and metagenome bins with predicted occurrences of the modules M1, M2, M3, and M4. Genomes from the learning set were excluded. Additional file 12:
**Gene cluster in the cow rumen draft genome AGa.** The red box in Figure S1 marks a gene cluster (NODE_457020_ORF_01660 to NODE_457020_ORF_01710), which was identified based on the families assigned to highest scoring module (M1). The cluster is located on a 97,191-bp contig of the draft genome AGa (Bacteroidales) from the cow rumen metagenome [19]. The cluster includes three cellulases, based on assignments of the GH5 family, and a cellobiose phosphorylase (GH94; EC 2.4.1.20) with an attached putative carbohydrate binding domain (PF06204). The GH94 family was not assigned to the consensus module of M1, but it was contained in the M1 modules in 7 of 18 LDA runs. Depending on the presence or absence of GH94 in the M1 modules of different runs, the gene cluster was identified either partly or completely. The cluster includes two genes (genes 01680 and 01690; green rectangle) without any annotated functional domains; these are uncharacterized genes that may be relevant for the degradation of lignocellulose. The presence of two Pfam families related to the major facilitator superfamily in gene 01640 (marked by the yellow box) indicates a link between the (hemi)cellulases of the GH5 and GH94 families, and sugar-binding or transport proteins located in the outer membrane (see Additional file 2: Section 10). Additional file 13:
**Protein sequences of the identified gene cluster in the cow rumen draft genome AGa.** Protein sequences (NODE_457020_ORF_01620 to NODE_457020_ORF_01740) from the cow rumen metagenome representing a gene cluster in the draft genome AGa (discussed in the main manuscript), as well as its surrounding genes. Additional file 14:
**Microbial isolate strains (lignocellulose degraders and non-degraders) that were used as the learning set.** Table S1 represents a manually curated list of 120 phenotype-positive or phenotype-negative prokaryotic genomes, including the respective literature references.

## Abbreviations

CAZyme: Carbohydrate-active enzyme
CBM: Carbohydrate-binding module
CE: Carbohydrate esterase
DUF: Domain of unknown function
EC: Enzyme classification
GH: Glycoside hydrolase
LDA: Latent Dirichlet allocation
PDM: Plant biomass degradation module
PL: Polysaccharide lyase
PUL: Polysaccharide utilization locus
Sus: Starch utilization system
SVM: Support vector machine
