# Supplementary material for: Inference of phenotype-defining functional modules of protein families for microbial plant biomass degraders
Source: Biotechnol Biofuels. 2014 Sep 9;7:124. doi: 10.1186/s13068-014-0124-8 (PMC4189754; doi:10.1186/s13068-014-0124-8)
Supplement: Additional file 1: — Protein families of the consensus plant biomass degradation modules (PDMs). The Tables S1A-5A show each consensus module as a list of Pfam/CAZy terms. The consensus modules summarize highly similar modules from the 18 LDA runs and contain all elements that occurred in nine runs or more. The tables S1B-5B contain information about all additional Pfam/CAZy families that occurred in the similar modules in less than nine runs. Tables S6A and S6B list the families of the additional PUL module (see Results section). [file 13068_2014_124_MOESM1_ESM.pdf]

Table S1A

M1 Consensus

Domain	# runs	Description
**GH5**	17	chitosanase (EC 3.2.1.132); beta-mannosidase (EC 3.2.1.25); Cellulase (EC 3.2.1.4); glucan 1,3-beta-glucosidase (EC 3.2.1.58); licheninase (EC 3.2.1.73); glucan endo-1,6-beta-glucosidase (EC 3.2.1.75); mannan endo-beta-1,4-mannosidase (EC 3.2.1.78); endo-beta-1,4-xylanase (EC 3.2.1.8); cellulose beta-1,4-cellobiosidase (EC 3.2.1.91); beta-1,3-mannanase (EC 3.2.1.-); xyloglucan-specific endo-beta-1,4-glucanase (EC 3.2.1.151); mannan transglycosylase (EC 2.4.1.-); endo-beta-1,6-galactanase (EC 3.2.1.164)
**PF00150**	18	Cellulase (glycosyl hydrolase family 5)
**GH9**	18	endoglucanase (EC 3.2.1.4); cellobiohydrolase (EC 3.2.1.91); beta-glucosidase (EC 3.2.1.21); exo-beta-glucosaminidase (EC 3.2.1.165)
**PF00759**	17	Glycosyl hydrolase family 9
**GH10**	17	endo-1,4-beta-xylanase (EC 3.2.1.8); endo-1,3-beta-xylanase (EC 3.2.1.32)
**PF00331**	17	Glycosyl hydrolase family 10
**GH26**	17	beta-mannanase (EC 3.2.1.78); beta-1,3-xylanase (EC 3.2.1.32)
**PF02156**	17	Glycosyl hydrolase family 26
**GH43**	18	beta-xylosidase (EC 3.2.1.37); beta-1,3-xylosidase (EC 3.2.1.-); alpha-L-arabinofuranosidase (EC 3.2.1.55); arabinanase (EC 3.2.1.99); xylanase (EC 3.2.1.8); galactan 1,3-beta-galactosidase (EC 3.2.1.145)
**PF04616**	17	Glycosyl hydrolases family 43
**CBM4**	16	Modules of approx. 150 residues found in bacterial enzymes. Binding of these modules has been demonstrated with xylan, beta-1,3-glucan, beta-1,3-1,4-glucan, beta-1,6-glucan and amorphous cellulose but not with crystalline cellulose.
**PF02018**	17	Carbohydrate binding domain (CBM_4_9)
**CBM6**	18	Modules of approx. 120 residues. The cellulose-binding function has been demonstrated in one case on amorphous cellulose and beta-1,4-xylan. Some of these modules also bind beta-1,3-glucan, beta-1,3-1,4-glucan, and beta-1,4-glucan.
**PF03422**	18	Carbohydrate binding module (family 6)
**PF02927**	17	N-terminal ig-like domain of cellulase
**CBM35**	17	Modules of approx. 130 residues. A module that is conserved in three Cellvibrio xylan-degrading enzymes binds to xylan and the interaction is calcium dependent, while a module from a Cellvibrio mannanase binds to decorated soluble mannans and mannooligosaccharides. A module in a
**PF00756**	13	Putative esterase
**PF13472**	10	GDSL-like Lipase/Acylhydrolase family

Table S1B

M1 Histogram

Domain	Description	# runs
**PF00150**	Cellulase (glycosyl hydrolase family 5)	18
**GH43**	beta-xylosidase (EC 3.2.1.37); beta-1,3-xylosidase (EC 3.2.1.-); alpha-L-arabin [...]	18
**CBM6**	Modules of approx. 120 residues. The cellulose-binding function has been demons [...]	18
**GH9**	endoglucanase (EC 3.2.1.4); cellobiohydrolase (EC 3.2.1.91); beta-glucosidase ([...]	18
**PF03422**	Carbohydrate binding module (family 6)	18
**GH5**	chitosanase (EC 3.2.1.132); beta-mannosidase (EC 3.2.1.25); Cellulase (EC 3.2.1 [...]	17
**PF02927**	N-terminal ig-like domain of cellulase	17
**GH10**	endo-1,4-beta-xylanase (EC 3.2.1.8); endo-1,3-beta-xylanase (EC 3.2.1.32)	17
**PF00759**	Glycosyl hydrolase family 9	17
**PF00331**	Glycosyl hydrolase family 10	17
**PF04616**	Glycosyl hydrolases family 43	17
**GH26**	beta-mannanase (EC 3.2.1.78); beta-1,3-xylanase (EC 3.2.1.32)	17
**CBM35**	Modules of approx. 130 residues. A module that is conserved in three Cellvibrio [...]	17
**PF02018**	Carbohydrate binding domain (CBM_4_9)	17
**PF02156**	Glycosyl hydrolase family 26	17
**CBM4**	Modules of approx. 150 residues found in bacterial enzymes. Binding of these mo [...]	16
**PF00756**	Putative esterase	13
**PF13472**	GDSL-like Lipase/Acylhydrolase family	10
**PF00657**	GDSL-like Lipase/Acylhydrolase	7
**PF00165**	Bacterial regulatory helix-turn-helix proteins, AraC family	7
**GH94**	cellobiose phosphorylase (EC 2.4.1.20); cellodextrin phosphorylase (EC 2.4.1.4 [...]	7
**PF06205**	Glycosyltransferase 36 associated family	5
**PF06165**	Glycosyltransferase family 36 (Deleted: now family GH94)	5
**GH117**	alpha-1,3-L-neoagarooligosaccharide hydrolase (EC 3.2.1.-); alpha-1,3-L-neoagar [...]	5
**CBM22**	A xylan binding function has been demonstrated in several cases and affinity wi [...]	4
**GH16**	xyloglucan:xyloglucosyltransferase (EC 2.4.1.207); keratan-sulfate endo-1,4-bet [...]	4
**PF06204**	Putative carbohydrate binding domain	4
**PF01882**	Protein of unknown function DUF58	4
**GH30**	glucosylceramidase (EC 3.2.1.45); beta-1,6-glucanase (EC 3.2.1.75); beta-xylosi [...]	4
**PF12833**	Helix-turn-helix domain	4
**CE3**	acetyl xylan esterase (EC 3.1.1.72).	4
**PF00722**	Glycosyl hydrolases family 16	4
**PF06964**	Alpha-L-arabinofuranosidase C-terminus	3
**GH51**	alpha-L-arabinofuranosidase (EC 3.2.1.55); endoglucanase (EC 3.2.1.4)	3
**GH8**	chitosanase (EC 3.2.1.132); cellulase (EC 3.2.1.4); licheninase (EC 3.2.1.73); [...]	3
**GH67**	alpha-glucuronidase (EC 3.2.1.139); xylan alpha-1,2-glucuronidase (EC 3.2.1.131)	3
**CBM36**	Modules of approx. 120-130 residues displaying structural similarities to CBM6 [...]	3
**PF01270**	Glycosyl hydrolases family 8	3
**PF00933**	Glycosyl hydrolase family 3 N terminal domain	3
**PF08757**	CotH protein	2
**PF07726**	ATPase family associated with various cellular activities (AAA)	2
**PF00942**	Cellulose binding domain	2
**PF13385**	Concanavalin A-like lectin/glucanases superfamily	2
**GH95**	alpha-1,2-L-fucosidase (EC 3.2.1.63); alpha-L-fucosidase (EC 3.2.1.51)	2
**GH3**	beta-glucosidase (EC 3.2.1.21); xylan 1,4-beta-xylosidase (EC 3.2.1.37); beta-N [...]	2
**PF00553**	Cellulose binding domain	2
**CBM3**	Modules of approx. 150 residues found in bacterial enzymes. The cellulose-bindi [...]	2

**GH39**	alpha-L-iduronidase (EC 3.2.1.76); beta-xylosidase (EC 3.2.1.37).	2
**PF01915**	Glycosyl hydrolase family 3 C-terminal domain	2
**PF07477**	Glycosyl hydrolase family 67 C-terminus	2
**PF07488**	Glycosyl hydrolase family 67 middle domain	2
**GH2**	beta-galactosidase (EC 3.2.1.23) ; beta-mannosidase (EC 3.2.1.25); beta-glucuro [...]	2
**PL1**	pectate lyase (EC 4.2.2.2); exo-pectate lyase (EC 4.2.2.9); pectin lyase (EC 4. [...]	2
**CBM2**	Modules of approx. 100 residues and which are found in a large number of bacter [...]	2
**PF02837**	Glycosyl hydrolases family 2, sugar binding domain	2
**CE2**	acetyl xylan esterase (EC 3.1.1.72).	2
**PF01055**	Glycosyl hydrolases family 31	1
**GH124**	endoglucanase (EC 3.2.1.4)	1
**PF13377**	Periplasmic binding protein-like domain	1
**GH36**	alpha-galactosidase (EC 3.2.1.22); alpha-N-acetylgalactosaminidase (EC 3.2.1.49 [...]	1
**PF07745**	Glycosyl hydrolase family 53	1
**PF00703**	Glycosyl hydrolases family 2	1
**dockerin**		1
**PF13407**	Periplasmic binding protein domain	1
**GH53**	endo-beta-1,4-galactanase (EC 3.2.1.89).	1
**PF01243**	Pyridoxamine 5'-phosphate oxidase	1
**PF01841**	Transglutaminase-like superfamily	1
**GH31**	alpha-glucosidase (EC 3.2.1.20); alpha-1,3-glucosidase (EC 3.2.1.84); sucrase-i [...]	1
**GH32**	invertase (EC 3.2.1.26); endo-inulinase (EC 3.2.1.7); beta-2,6-fructan 6-levanb [...]	1
**PF07691**	PA14 domain	1
**PF00544**	Pectate lyase	1
**PF02836**	Glycosyl hydrolases family 2, TIM barrel domain	1
**PF00457**	Glycosyl hydrolases family 11	1
**PF14310**	Fibronectin type III-like domain	1
**PF00404**	Dockerin type I repeat	1
**GH11**	xylanase (EC 3.2.1.8)	1
**PF00532**	Periplasmic binding proteins and sugar binding domain of LacI family	1

Table S2A

M2 Consensus

Domain	# runs	Description
**GH16**	16	xyloglucan:xyloglucosyltransferase (EC 2.4.1.207); keratan-sulfate endo-1,4-beta-galactosidase (EC 3.2.1.103); endo-1,3-beta-glucanase (EC 3.2.1.39); endo-1,3(4)-beta-glucanase (EC 3.2.1.6); licheninase (EC 3.2.1.73); beta-agarase (EC 3.2.1.81); kappa;-carrageenase (EC 3.2.1.83); xyloglucanase (EC 3.2.1.151)
**PF00722**	17	Glycosyl hydrolases family 16
**GH30**	10	glucosylceramidase (EC 3.2.1.45); beta-1,6-glucanase (EC 3.2.1.75); beta-xylosidase (EC 3.2.1.37); beta-fucosidase (EC 3.2.1.38); beta-glucosidase (3.2.1.21); endo-beta-1,6-galactanase (EC:3.2.1.164)
**CBM6**	18	Modules of approx. 120 residues. The cellulose-binding function has been demonstrated in one case on amorphous cellulose and beta-1,4-xylan. Some of these modules also bind beta-1,3-glucan, beta-1,3-1,4-glucan, and beta-1,4-glucan.
**PF03422**	18	Carbohydrate binding module (family 6)
**CBM16**	16	Carbohydrate-binding module 16. Binding to cellulose and glucomannan demonstrated [B. Bae et al (2008) J Biol Chem. 283:12415-25 (PMID: 18025086)]
**CBM35**	18	Modules of approx. 130 residues. A module that is conserved in three Cellvibrio xylan-degrading enzymes binds to xylan and the interaction is calcium dependent, while a module from a Cellvibrio mannanase binds to decorated soluble mannans and mannooligosaccharides. A module in a
**CBM61**	17	Modules of approx. 150 residues found appended to GH16, GH30, GH31, GH43, GH53 and GH66 catalytic domains. A beta-1,4-galactan binding function has been demonstrated for the CBM60 of Thermotoga maritima GH53 galactanase [PMID: 20826814].
**CBM47**	17	Modules of approx 150 residues. Fucose-binding activity demonstrated
**CBM32**	17	Binding to galactose and lactose has been demonstrated for the module of Micromonospora viridifaciens sialidase (PMID: 16239725). Binding to polygalacturonic acid has been shown for a Yersinia member (PMID: 17292916). Binding to LacNAc (beta-D-galactosyl-1,4-beta-D-N-acetylglucosamine) has been shown for an N-acetylglucosaminidase from Clostridium perfingens (PMID: 16990278). (Distantly related to CBM6 modules and to Anguilla anguilla agglutinin.)
**CBM13**	11	Modules of approx. 150 residues which always appear as a threefold internal repeat. The only apparent exception to this, xylanase II of Actinomadura sp. FC7 (GenBank U08894), is in fact not completely sequenced. These modules were first identified in several plant lectins such as ricin or agglutinin of Ricinus communis which bind galactose residues. The three-dimensional structure of a plant lectin has been determined and displays a pseudo-threefold symmetry in accord with the observed sequence threefold repeat. These modules have since been found in a number of other proteins of various functions including glycoside hydrolases and glycosyltransferases. While in the plant lectins this module binds mannose, binding to xylan has been demonstrated in the Streptomyces lividans xylanase A and arabinofuranosidase B. Binding to GalNAc has been shown for the corresponding module of GalNAc transferase 4. For the other proteins, the binding specificity of these modules has not been established. The pseudo three-fold symmetry of the CBM13 module has now been confirmed in the 3-D structure of the intact, two-domain, xylanase of Streptomyces olivaceoviridis.
**PF14200**	11	Ricin-type beta-trefoil lectin domain-like
**PF00652**	11	Ricin-type beta-trefoil lectin domain
**GH87**	17	mycodextranase (EC 3.2.1.61); alpha-1,3-glucanase (EC 3.2.1.59)
**PF00754**	17	F5/8 type C domain
**PF00041**	17	Fibronectin type III domain
**GH119**	17	alpha-amylase (EC 3.2.1.1) (Distantly related to family GH57)

**PF12708**	14	Pectate lyase superfamily protein
**PF02311**	13	AraC-like ligand binding domain
**PF02018**	13	Carbohydrate binding domain (CBM_4_9)
**GH55**	12	exo-beta-1,3-glucanase (EC 3.2.1.58); endo-beta-1,3-glucanase (EC 3.2.1.39).
**PF13483**	9	Beta-lactamase superfamily domain

Table S2B

M2 Histogram

Domain	Description	# runs
**CBM6**	Modules of approx. 120 residues. The cellulose-binding function has been demons [...]	18
**PF03422**	Carbohydrate binding module (family 6)	18
**CBM35**	Modules of approx. 130 residues. A module that is conserved in three Cellvibrio [...]	18
**PF00754**	F5/8 type C domain	17
**CBM47**	Modules of approx 150 residues. Fucose-binding activity demonstrated	17
**CBM32**	Binding to galactose and lactose has been demonstrated for the module of Microm [...]	17
**GH87**	mycodextranase (EC 3.2.1.61); alpha-1,3-glucanase (EC 3.2.1.59)	17
**PF00041**	Fibronectin type III domain	17
**PF00722**	Glycosyl hydrolases family 16	17
**GH119**	alpha-amylase (EC 3.2.1.1) (Distantly related to family GH57)	17
**CBM61**	Modules of approx. 150 residues found appended to GH16, GH30, GH31, GH43, GH53 [...]	17
**GH16**	xyloglucan:xyloglucosyltransferase (EC 2.4.1.207); keratan-sulfate endo-1,4-bet [...]	16
**CBM16**	Carbohydrate-binding module 16. Binding to cellulose and glucomannan demonstrat [...]	16
**PF12708**	Pectate lyase superfamily protein	14
**PF02311**	AraC-like ligand binding domain	13
**PF02018**	Carbohydrate binding domain (CBM_4_9)	13
**GH55**	exo-beta-1,3-glucanase (EC 3.2.1.58); endo-beta-1,3-glucanase (EC 3.2.1.39).	12
**PF14200**	Ricin-type beta-trefoil lectin domain-like	11
**CBM13**	Modules of approx. 150 residues which always appear as a threefold internal rep [...]	11
**PF00652**	Ricin-type beta-trefoil lectin domain	11
**GH30**	glucosylceramidase (EC 3.2.1.45); beta-1,6-glucanase (EC 3.2.1.75); beta-xylosi [...]	10
**PF13483**	Beta-lactamase superfamily domain	9
**GH18**	chitinase (EC 3.2.1.14); endo-beta-N-acetylglucosaminidase (EC 3.2.1.96); xylan [...]	8
**PF00704**	Glycosyl hydrolases family 18	8
**GH109**	alpha-N-acetylgalactosaminidase (EC 3.2.1.49)	6
**PF02894**	Oxidoreductase family, C-terminal alpha/beta domain	6
**PF13385**	Concanavalin A-like lectin/glucanases superfamily	6
**PF13229**	Right handed beta helix region	6
**PF02055**	O-Glycosyl hydrolase family 30	5
**PF06271**	RDD family	5
**PF13527**	Acetyltransferase (GNAT) domain	4
**PF00801**	PKD domain	3
**PF00553**	Cellulose binding domain	3
**PF05721**	Phytanoyl-CoA dioxygenase (PhyH)	3
**CBM2**	Modules of approx. 100 residues and which are found in a large number of bacter [...]	3
**CBM4**	Modules of approx. 150 residues found in bacterial enzymes. Binding of these mo [...]	3
**GH5**	chitosanase (EC 3.2.1.132); beta-mannosidase (EC 3.2.1.25); Cellulase (EC 3.2.1 [...]	2
**PF01638**	HxlR-like helix-turn-helix	2
**PF13463**	Winged helix DNA-binding domain	2
**GH3**	beta-glucosidase (EC 3.2.1.21); xylan 1,4-beta-xylosidase (EC 3.2.1.37); beta-N [...]	2
**CE3**	acetyl xylan esterase (EC 3.1.1.72).	2
**PF00165**	Bacterial regulatory helix-turn-helix proteins, AraC family	2
**PF00933**	Glycosyl hydrolase family 3 N terminal domain	2
**GH2**	beta-galactosidase (EC 3.2.1.23) ; beta-mannosidase (EC 3.2.1.25); beta-glucuro [...]	2
**PL9**	pectate lyase (EC 4.2.2.2); exopolygalacturonate lyase (EC 4.2.2.9); thiopeptid [...]	2
**CBM22**	A xylan binding function has been demonstrated in several cases and affinity wi [...]	1
**PF08757**	CotH protein	1
**GH92**	mannosyl-oligosaccharide alpha-1,2-mannosidase (EC 3.2.1.113); mannosyl-oligosa [...]	1

**PF06205**	Glycosyltransferase 36 associated family	1
**PF06165**	Glycosyltransferase family 36 (Deleted: now family GH94)	1
**PF00150**	Cellulase (glycosyl hydrolase family 5)	1
**PF13377**	Periplasmic binding protein-like domain	1
**PF00480**	ROK family	1
**PF06204**	Putative carbohydrate binding domain	1
**PF00756**	Putative esterase	1
**PF02927**	N-terminal ig-like domain of cellulase	1
**GH10**	endo-1,4-beta-xylanase (EC 3.2.1.8); endo-1,3-beta-xylanase (EC 3.2.1.32)	1
**PF12679**	ABC-2 family transporter protein	1
**PF00144**	Beta-lactamase	1
**PF00759**	Glycosyl hydrolase family 9	1
**PF07971**	Glycosyl hydrolase family 92	1
**PF00657**	GDSL-like Lipase/Acylhydrolase	1
**PF00331**	Glycosyl hydrolase family 10	1
**GH43**	beta-xylosidase (EC 3.2.1.37); beta-1,3-xylosidase (EC 3.2.1.-); alpha-L-arabin [...]	1
**PF13407**	Periplasmic binding protein domain	1
**CBM5**	Modules of approx. 60 residues found in bacterial enzymes. Chitin-binding descr [...]	1
**PF13472**	GDSL-like Lipase/Acylhydrolase family	1
**PF01522**	Polysaccharide deacetylase	1
**GT41**	UDP-GlcNAc: peptide beta-N-acetylglucosaminyltransferase (EC 2.4.1.94)	1
**GH9**	endoglucanase (EC 3.2.1.4); cellobiohydrolase (EC 3.2.1.91); beta-glucosidase ([...]	1
**PF03935**	Beta-glucan synthesis-associated protein (SKN1)	1
**PF13519**	von Willebrand factor type A domain	1
**CE4**	acetyl xylan esterase (EC 3.1.1.72); chitin deacetylase (EC 3.5.1.41); chitool [...]	1
**CBM44**	The C-terminal CBM44 module of the Clostridium thermocellum enzyme has been dem [...]	1
**CBM12**	Modules of approx. 40-60 residues. The majority of these modules is found among [...]	1
**PF12833**	Helix-turn-helix domain	1
**PF04616**	Glycosyl hydrolases family 43	1
**GH94**	cellobiose phosphorylase (EC 2.4.1.20); cellodextrin phosphorylase (EC 2.4.1.4 [...]	1
**PF01915**	Glycosyl hydrolase family 3 C-terminal domain	1
**GH26**	beta-mannanase (EC 3.2.1.78); beta-1,3-xylanase (EC 3.2.1.32)	1
**PF02839**	Carbohydrate binding domain	1
**GH59**	galactocerebrosidase (EC 3.2.1.46)	1
**PF00532**	Periplasmic binding proteins and sugar binding domain of LacI family	1
**PF02156**	Glycosyl hydrolase family 26	1
**CE7**	acetyl xylan esterase (EC 3.1.1.72); cephalosporin-C deacetylase (EC 3.1.1.41).	1
**PF00082**	Subtilase family	1

Table S3A

M3 Consensus

Domain	# runs	Description
**GH5**	18	chitosanase (EC 3.2.1.132); beta-mannosidase (EC 3.2.1.25); Cellulase (EC 3.2.1.4); glucan 1,3-beta-glucosidase (EC 3.2.1.58); licheninase (EC 3.2.1.73); glucan endo-1,6-beta-glucosidase (EC 3.2.1.75); mannan endo-beta-1,4-mannosidase (EC 3.2.1.78); endo-beta-1,4-xylanase (EC 3.2.1.8); cellulose beta-1,4-cellobiosidase (EC 3.2.1.91); beta-1,3-mannanase (EC 3.2.1.-); xyloglucan-specific endo-beta-1,4-glucanase (EC 3.2.1.151); mannan transglycosylase (EC 2.4.1.-); endo-beta-1,6-galactanase (EC 3.2.1.164)
**GH43**	12	beta-xylosidase (EC 3.2.1.37); beta-1,3-xylosidase (EC 3.2.1.-); alpha-L-arabinofuranosidase (EC 3.2.1.55); arabinanase (EC 3.2.1.99); xylanase (EC 3.2.1.8); galactan 1,3-beta-galactosidase (EC 3.2.1.145)
**PF04616**	12	Glycosyl hydrolases family 43
**PF03629**	17	Domain of unknown function (DUF303)
**PF01095**	18	Pectinesterase
**PL1**	18	pectate lyase (EC 4.2.2.2); exo-pectate lyase (EC 4.2.2.9); pectin lyase (EC 4.2.2.10).
**PF12708**	18	Pectate lyase superfamily protein
**GH28**	18	polygalacturonase (EC 3.2.1.15); exo-polygalacturonase (EC 3.2.1.67); exo-polygalacturonosidase (EC 3.2.1.82); rhamnogalacturonase (EC 3.2.1.-); endo-xylogalacturonan hydrolase (EC 3.2.1.-); rhamnogalacturonan alpha-L-rhamnopyranohydrolase (EC 3.2.1.40)
**PF00295**	18	Glycosyl hydrolases family 28
**CE6**	12	acetyl xylan esterase (EC 3.1.1.72).
**CE7**	18	acetyl xylan esterase (EC 3.1.1.72); cephalosporin-C deacetylase (EC 3.1.1.41).
**CE8**	18	pectin methylesterase (EC 3.1.1.11).
**CE12**	18	pectin acetylesterase (EC 3.1.1.-); rhamnogalacturonan acetylesterase (EC 3.1.1.-); acetyl xylan esterase (EC 3.1.1.72)
**PL9**	13	pectate lyase (EC 4.2.2.2); exopolygalacturonate lyase (EC 4.2.2.9); thiopeptidoglycan lyase (EC 4.2.2.-).
**GH106**	16	alpha-L-rhamnosidase (EC 3.2.1.40)
**GH88**	14	d-4,5 unsaturated beta-glucuronyl hydrolase (EC 3.2.1.-)
**PF07470**	18	Glycosyl Hydrolase Family 88
**GH105**	18	unsaturated rhamnogalacturonyl hydrolase (EC 3.2.1.-)
**PF00657**	18	GDSL-like Lipase/Acylhydrolase
**PF13229**	18	Right handed beta helix region
**GH95**	17	alpha-1,2-L-fucosidase (EC 3.2.1.63); alpha-L-fucosidase (EC 3.2.1.51)
**PF13472**	15	GDSL-like Lipase/Acylhydrolase family
**PF13524**	13	Glycosyl transferases group 1

Table S3B

M3 Histogram

Domain	Description	# runs
**PF01095**	Pectinesterase	18
**GH5**	chitosanase (EC 3.2.1.132); beta-mannosidase (EC 3.2.1.25); Cellulase (EC 3.2.1 [...]	18
**CE12**	pectin acetylesterase (EC 3.1.1.-); rhamnogalacturonan acetylesterase (EC 3.1.1 [...]	18
**GH28**	polygalacturonase (EC 3.2.1.15); exo-polygalacturonase (EC 3.2.1.67); exo-polyg [...]	18
**GH105**	unsaturated rhamnogalacturonyl hydrolase (EC 3.2.1.-)	18
**PF00657**	GDSL-like Lipase/Acylhydrolase	18
**PF00295**	Glycosyl hydrolases family 28	18
**PF07470**	Glycosyl Hydrolase Family 88	18
**PL1**	pectate lyase (EC 4.2.2.2); exo-pectate lyase (EC 4.2.2.9); pectin lyase (EC 4. [...]	18
**PF12708**	Pectate lyase superfamily protein	18
**PF13229**	Right handed beta helix region	18
**CE7**	acetyl xylan esterase (EC 3.1.1.72); cephalosporin-C deacetylase (EC 3.1.1.41).	18
**CE8**	pectin methylesterase (EC 3.1.1.11).	18
**GH95**	alpha-1,2-L-fucosidase (EC 3.2.1.63); alpha-L-fucosidase (EC 3.2.1.51)	17
**PF03629**	Domain of unknown function (DUF303)	17
**GH106**	alpha-L-rhamnosidase (EC 3.2.1.40)	16
**PF13472**	GDSL-like Lipase/Acylhydrolase family	15
**GH88**	d-4,5 unsaturated beta-glucuronyl hydrolase (EC 3.2.1.-)	14
**PF13524**	Glycosyl transferases group 1	13
**PL9**	pectate lyase (EC 4.2.2.2); exopolygalacturonate lyase (EC 4.2.2.9); thiopeptid [...]	13
**PF04616**	Glycosyl hydrolases family 43	12
**CE6**	acetyl xylan esterase (EC 3.1.1.72).	12
**GH43**	beta-xylosidase (EC 3.2.1.37); beta-1,3-xylosidase (EC 3.2.1.-); alpha-L-arabin [...]	12
**PL11**	rhamnogalacturonan lyase (EC 4.2.2.-); exo-unsaturated rhamnogalacturonan lyase [...]	8
**PF07944**	Putative glycosyl hydrolase of unknown function (DUF1680)	8
**GH115**	xylan alpha-1,2-glucuronidase (3.2.1.131); alpha-(4-O-methyl)-glucuronidase (3. [...]	7
**GH32**	invertase (EC 3.2.1.26); endo-inulinase (EC 3.2.1.7); beta-2,6-fructan 6-levanb [...]	6
**GH117**	alpha-1,3-L-neoagarooligosaccharide hydrolase (EC 3.2.1.-); alpha-1,3-L-neoagar [...]	6
**PF13632**	Glycosyl transferase family group 2	6
**PF07940**	Heparinase II/III-like protein	5
**GH78**	alpha-L-rhamnosidase (EC 3.2.1.40)	5
**PF05448**	Acetyl xylan esterase (AXE1)	4
**GH51**	alpha-L-arabinofuranosidase (EC 3.2.1.55); endoglucanase (EC 3.2.1.4)	4
**PF06964**	Alpha-L-arabinofuranosidase C-terminus	3
**PF05592**	Bacterial alpha-L-rhamnosidase	3
**PF00532**	Periplasmic binding proteins and sugar binding domain of LacI family	3
**PL12**	heparin-sulfate lyase (EC 4.2.2.8)	2
**PF00703**	Glycosyl hydrolases family 2	2
**CBM32**	Binding to galactose and lactose has been demonstrated for the module of Microm [...]	2
**PF07495**	Y_Y_Y domain	2
**GH97**	alpha-glucosidase (EC 3.2.1.20); alpha-galactosidase (EC 3.2.1.22)	2
**PF00756**	Putative esterase	1
**PF08666**	SAF domain	1
**PF02836**	Glycosyl hydrolases family 2, TIM barrel domain	1
**PF01263**	Aldose 1-epimerase	1
**GH55**	exo-beta-1,3-glucanase (EC 3.2.1.58); endo-beta-1,3-glucanase (EC 3.2.1.39).	1
**GH2**	beta-galactosidase (EC 3.2.1.23) ; beta-mannosidase (EC 3.2.1.25); beta-glucuro [...]	1

**PL17**	alginate lyase (EC 4.2.2.3).	1
**PF02837**	Glycosyl hydrolases family 2, sugar binding domain	1
**PF08531**	Alpha-L-rhamnosidase N-terminal domain	1
**PF13407**	Periplasmic binding protein domain	1

Table S4A

M4 Consensus

Domain	# runs	Description
**GH5**	18	chitosanase (EC 3.2.1.132); beta-mannosidase (EC 3.2.1.25); Cellulase (EC 3.2.1.4); glucan 1,3-beta-glucosidase (EC 3.2.1.58); licheninase (EC 3.2.1.73); glucan endo-1,6-beta-glucosidase (EC 3.2.1.75); mannan endo-beta-1,4-mannosidase (EC 3.2.1.78); endo-beta-1,4-xylanase (EC 3.2.1.8); cellulose beta-1,4-cellobiosidase (EC 3.2.1.91); beta-1,3-mannanase (EC 3.2.1.-); xyloglucan-specific endo-beta-1,4-glucanase (EC 3.2.1.151); mannan transglycosylase (EC 2.4.1.-); endo-beta-1,6-galactanase (EC 3.2.1.164)
**PF00150**	13	Cellulase (glycosyl hydrolase family 5)
**GH43**	16	beta-xylosidase (EC 3.2.1.37); beta-1,3-xylosidase (EC 3.2.1.-); alpha-L-arabinofuranosidase (EC 3.2.1.55); arabinanase (EC 3.2.1.99); xylanase (EC 3.2.1.8); galactan 1,3-beta-galactosidase (EC 3.2.1.145)
**PF04616**	15	Glycosyl hydrolases family 43
**GH2**	18	beta-galactosidase (EC 3.2.1.23) ; beta-mannosidase (EC 3.2.1.25); beta-glucuronidase (EC 3.2.1.31); mannosylglycoprotein endo-beta-mannosidase (EC 3.2.1.152); exo-beta-glucosaminidase (EC 3.2.1.165)
**PF02836**	18	Glycosyl hydrolases family 2, TIM barrel domain
**PF00703**	18	Glycosyl hydrolases family 2
**PF02837**	18	Glycosyl hydrolases family 2, sugar binding domain
**GH3**	18	beta-glucosidase (EC 3.2.1.21); xylan 1,4-beta-xylosidase (EC 3.2.1.37); beta-N-acetylhexosaminidase (EC 3.2.1.52); glucan 1,3-beta-glucosidase (EC 3.2.1.58); glucan 1,4-beta-glucosidase (EC 3.2.1.74); exo-1,3-1,4-glucanase (EC 3.2.1.-); alphalpha-L-arabinofuranosidase (EC 3.2.1.55).
**PF01915**	18	Glycosyl hydrolase family 3 C-terminal domain
**PF00933**	18	Glycosyl hydrolase family 3 N terminal domain
**GH35**	9	beta-galactosidase (EC 3.2.1.23); exo-beta-glucosaminidase (EC 3.2.1.165)
**PF02449**	10	Beta-galactosidase
**GH42**	10	beta-galactosidase (EC 3.2.1.23)
**PF02065**	17	Melibiase (GH27) [GH-D clan, a superfamily of alpha-galactosidases]
**GH31**	18	alpha-glucosidase (EC 3.2.1.20); alpha-1,3-glucosidase (EC 3.2.1.84); sucrase-isomaltase (EC 3.2.1.48) (EC 3.2.1.10); alpha-xylosidase (EC 3.2.1.-); alpha-glucan lyase (EC 4.2.2.13); isomaltosyltransferase (EC 2.4.1.-). [GH-D clan, a superfamily of alpha-galactosidases]
**PF01055**	18	Glycosyl hydrolases family 31
**GH36**	17	alpha-galactosidase (EC 3.2.1.22); alpha-N-acetylgalactosaminidase (EC 3.2.1.49); stachyose synthase (EC 2.4.1.67); raffinose synthase (EC 2.4.1.82) [GH-D clan, a superfamily of alpha-galactosidases]
**PF14310**	18	Fibronectin type III-like domain
**PF07859**	15	alpha/beta hydrolase fold
**CE10**	14	arylesterase (EC 3.1.1.-); carboxyl esterase (EC 3.1.1.3); acetylcholinesterase (EC 3.1.1.7); cholinesterase (EC 3.1.1.8); sterol esterase (EC 3.1.1.13); brefeldin A esterase (EC 3.1.1.-).
**GH32**	12	invertase (EC 3.2.1.26); endo-inulinase (EC 3.2.1.7); beta-2,6-fructan 6-levanbiohydrolase (EC 3.2.1.64); endo-levanase (EC 3.2.1.65); exo-inulinase (EC 3.2.1.80); fructan beta-(2,1)-fructosidase/1-exohydrolase (EC 3.2.1.153); fructan beta-(2,6)-fructosidase/6-exohydrolase (EC 3.2.1.154); sucrose:sucrose 1-fructosyltransferase (EC 2.4.1.99); fructan:fructan 1-fructosyltransferase (EC 2.4.1.100); sucrose:fructan 6-fructosyltransferase (EC 2.4.1.10); fructan:fructan 6G-fructosyltransferase (EC 2.4.1.243); levan fructosyltransferase (EC 2.4.1.-)
**PF00135**	10	Carboxylesterase family
**PF13802**	9	Galactose mutarotase-like
**GH106**	9	alpha-L-rhamnosidase (EC 3.2.1.40)

Table S4B

M4 Histogram

Domain	Description	# runs
**PF01055**	Glycosyl hydrolases family 31	18
**GH5**	chitosanase (EC 3.2.1.132); beta-mannosidase (EC 3.2.1.25); Cellulase (EC 3.2.1 [...]	18
**GH31**	alpha-glucosidase (EC 3.2.1.20); alpha-1,3-glucosidase (EC 3.2.1.84); sucrase-i [...]	18
**PF02836**	Glycosyl hydrolases family 2, TIM barrel domain	18
**PF01915**	Glycosyl hydrolase family 3 C-terminal domain	18
**PF00933**	Glycosyl hydrolase family 3 N terminal domain	18
**PF00703**	Glycosyl hydrolases family 2	18
**GH2**	beta-galactosidase (EC 3.2.1.23) ; beta-mannosidase (EC 3.2.1.25); beta-glucuro [...]	18
**PF14310**	Fibronectin type III-like domain	18
**GH3**	beta-glucosidase (EC 3.2.1.21); xylan 1,4-beta-xylosidase (EC 3.2.1.37); beta-N [...]	18
**PF02837**	Glycosyl hydrolases family 2, sugar binding domain	18
**PF02065**	Melibiase (GH27) [GH-D clan, a superfamily of alpha-galactosidases]	17
**GH36**	alpha-galactosidase (EC 3.2.1.22); alpha-N-acetylgalactosaminidase (EC 3.2.1.49 [...]	17
**GH43**	beta-xylosidase (EC 3.2.1.37); beta-1,3-xylosidase (EC 3.2.1.-); alpha-L-arabin [...]	16
**PF04616**	Glycosyl hydrolases family 43	15
**PF07859**	alpha/beta hydrolase fold	15
**CE10**	arylesterase (EC 3.1.1.-); carboxyl esterase (EC 3.1.1.3); acetylcholinesteras [...]	14
**PF00150**	Cellulase (glycosyl hydrolase family 5)	13
**GH32**	invertase (EC 3.2.1.26); endo-inulinase (EC 3.2.1.7); beta-2,6-fructan 6-levanb [...]	12
**PF02449**	Beta-galactosidase	10
**GH42**	beta-galactosidase (EC 3.2.1.23)	10
**PF00135**	Carboxylesterase family	10
**PF13802**	Galactose mutarotase-like	9
**GH35**	beta-galactosidase (EC 3.2.1.23); exo-beta-glucosaminidase (EC 3.2.1.165)	9
**GH106**	alpha-L-rhamnosidase (EC 3.2.1.40)	9
**PF08532**	Beta-galactosidase trimerisation domain	8
**PF00165**	Bacterial regulatory helix-turn-helix proteins, AraC family	7
**GH51**	alpha-L-arabinofuranosidase (EC 3.2.1.55); endoglucanase (EC 3.2.1.4)	7
**GH78**	alpha-L-rhamnosidase (EC 3.2.1.40)	7
**GH27**	alpha-galactosidase (EC 3.2.1.22); alpha-N-acetylgalactosaminidase (EC 3.2.1.49 [...]	7
**PF02929**	Beta galactosidase small chain	7
**PF06964**	Alpha-L-arabinofuranosidase C-terminus	6
**PF12833**	Helix-turn-helix domain	6
**PF05592**	Bacterial alpha-L-rhamnosidase	6
**PF02311**	AraC-like ligand binding domain	6
**PF08533**	Beta-galactosidase C-terminal domain	5
**PF07944**	Putative glycosyl hydrolase of unknown function (DUF1680)	5
**PF08531**	Alpha-L-rhamnosidase N-terminal domain	5
**GH39**	alpha-L-iduronidase (EC 3.2.1.76); beta-xylosidase (EC 3.2.1.37).	4
**PF00532**	Periplasmic binding proteins and sugar binding domain of LacI family	4
**PF13407**	Periplasmic binding protein domain	4
**PF00596**	Class II Aldolase and Adducin N-terminal domain	3
**PF13377**	Periplasmic binding protein-like domain	3
**PF01301**	Glycosyl hydrolases family 35	3
**GH1**	beta-glucosidase (EC 3.2.1.21); beta-galactosidase (EC 3.2.1.23); beta-mannosid [...]	3
**PF00356**	Bacterial regulatory proteins, lacI family	3
**PF00480**	ROK family	2

**PF00756**	Putative esterase	2
**PF00251**	Glycosyl hydrolases family 32 N-terminal domain	2
**PF00232**	Glycosyl hydrolase family 1	2
**PF04397**	LytTr DNA-binding domain	2
**PF10509**	Galactokinase galactose-binding signature	1
**PF02397**	Bacterial sugar transferase	1
**PF01074**	Glycosyl hydrolases family 38 N-terminal domain	1
**GH117**	alpha-1,3-L-neoagarooligosaccharide hydrolase (EC 3.2.1.-); alpha-1,3-L-neoagar [...]	1
**PF09261**	Alpha mannosidase, middle domain	1
**PF01757**	Acyltransferase family	1
**PF13524**	Glycosyl transferases group 1	1
**PF01663**	Type I phosphodiesterase / nucleotide pyrophosphatase	1
**PF01263**	Aldose 1-epimerase	1
**PF02614**	Glucuronate isomerase	1
**PF07748**	Glycosyl hydrolases family 38 C-terminal domain	1
**GH38**	alpha-mannosidase (EC 3.2.1.24) ; mannosyl-oligosaccharide alpha-1,3-1,6-mannos [...]	1
**PF06580**	Histidine kinase	1
**PF13472**	GDSL-like Lipase/Acylhydrolase family	1

Table S5A

M5 Consensus

Domain	# runs	Description
**PF00942**	16	Cellulose binding domain
**GH124**	16	endoglucanase (EC 3.2.1.4)
**CBM3**	16	Modules of approx. 150 residues found in bacterial enzymes. The cellulose-binding function has been demonstrated in many cases. In one instance binding to chitin has been reported.
**dockerin**		
**PF00404**	16	Dockerin type I repeat
**cohesin**		
**PF00963**	16	Cohesin domain
**PF07591**	16	Pretoxin HINT domain
**PF13186**	16	Domain of unknown function (DUF4008)
**CBM36**	15	Modules of approx. 120-130 residues displaying structural similarities to CBM6 modules. The only CBM36 currently characterised, that from Paenbacillus polymyxa xylanase 43A, shows calcium-dependent binding of xylans and xylooligosaccharides. X-ray crystallography shows that there is a direct interaction between calcium and ligand.
**PF05593**	12	RHS Repeat
**PF07238**	10	PilZ domain
**PF13403**	9	Hint domain

Table S5B

M5 Histogram

Domain	Description	# runs
**PF00942**	Cellulose binding domain	16
**GH124**	endoglucanase (EC 3.2.1.4)	16
**PF00404**	Dockerin type I repeat	16
**CBM3**	Modules of approx. 150 residues found in bacterial enzymes. The cellulose-bindi [...]	16
**dockerin**		16
**cohesin**		16
**PF07591**	Pretoxin HINT domain	16
**PF13186**	Domain of unknown function (DUF4008)	16
**PF00963**	Cohesin domain	16
**CBM36**	Modules of approx. 120-130 residues displaying structural similarities to CBM6 [...]	15
**PF05593**	RHS Repeat	12
**PF07238**	PilZ domain	10
**PF13403**	Hint domain	9
**PF00759**	Glycosyl hydrolase family 9	7
**GH9**	endoglucanase (EC 3.2.1.4); cellobiohydrolase (EC 3.2.1.91); beta-glucosidase ([...]	7
**PF01584**	CheW-like domain	5
**PF12791**	Anti-sigma factor N-terminus	5
**CBM35**	Modules of approx. 130 residues. A module that is conserved in three Cellvibrio [...]	5
**PF12833**	Helix-turn-helix domain	4
**PF03422**	Carbohydrate binding module (family 6)	2
**PF12730**	ABC-2 family transporter protein	2
**PF00150**	Cellulase (glycosyl hydrolase family 5)	2
**GH5**	chitosanase (EC 3.2.1.132); beta-mannosidase (EC 3.2.1.25); Cellulase (EC 3.2.1 [...]	2
**PF00239**	Resolvase, N terminal domain	2
**PF02018**	Carbohydrate binding domain (CBM_4_9)	2
**PF13231**	Dolichyl-phosphate-mannose-protein mannosyltransferase	2
**CBM6**	Modules of approx. 120 residues. The cellulose-binding function has been demons [...]	1
**PF07508**	Recombinase	1
**PF01797**	Transposase IS200 like	1

Table S6A

PUL module consensus

Domain	# runs	Description
**PF07980**	17	SusD family
**PF00593**	17	TonB dependent receptor
**PF07715**	17	TonB-dependent Receptor Plug Domain
**PF14322**	17	Starch-binding associating with outer membrane
**PF13715**	17	Cna protein B-type domain
**PF13620**	16	Carboxypeptidase regulatory-like domain
**PF13568**	13	Outer membrane protein beta-barrel domain
**PF00691**	13	OmpA family
**PF13505**	12	Outer membrane protein beta-barrel domain
**PF02321**	11	Outer membrane efflux protein

Table S6B

PUL module histogram

Domain	Description	# runs
**PF07980**	SusD family	17
**PF00593**	TonB dependent receptor	17
**PF07715**	TonB-dependent Receptor Plug Domain	17
**PF14322**	Starch-binding associating with outer membrane	17
**PF13715**	Cna protein B-type domain	17
**PF13620**	Carboxypeptidase regulatory-like domain	16
**PF13568**	Outer membrane protein beta-barrel domain	13
**PF00691**	OmpA family	13
**PF13505**	Outer membrane protein beta-barrel domain	12
**PF02321**	Outer membrane efflux protein	11
**PF12833**	Helix-turn-helix domain	7
**PF01551**	Peptidase family M23	5
**PF00165**	Bacterial regulatory helix-turn-helix proteins, AraC family	5
**PF03544**	Gram-negative bacterial tonB protein	4
**GT41**	UDP-GlcNAc: peptide beta-N-acetylglucosaminyltransferase (EC 2.4.1.94)	4
**PF13437**	HlyD family secretion protein	4
**PF12700**	HlyD family secretion protein	4
**CE10**	arylesterase (EC 3.1.1.-); carboxyl esterase (EC 3.1.1.3); acetylcholinesteras [...]	3
**PF13533**	Biotin-lipoyl like	2
**PF00529**	HlyD family secretion protein	1
**PF14289**	Domain of unknown function (DUF4369)	1
**PF13187**	4Fe-4S dicluster domain	1
**PF00873**	AcrB/AcrD/AcrF family	1
**PF12771**	Starch-binding associating with outer membrane	1
**PF03572**	Peptidase family S41	1
**PF00196**	Bacterial regulatory proteins, luxR family	1
**PF12741**	Susd and RagB outer membrane lipoprotein	1
**PF00082**	Subtilase family	1
