# Supplementary material for: Inference of phenotype-defining functional modules of protein families for microbial plant biomass degraders
Source: Biotechnol Biofuels. 2014 Sep 9;7:124. doi: 10.1186/s13068-014-0124-8 (PMC4189754; doi:10.1186/s13068-014-0124-8)
Supplement: Additional file 2: — Supplementary Note. Additional details about the results of the main manuscript. [file 13068_2014_124_MOESM2_ESM.pdf]

Inference of phenotype-defining functional modules of protein families for microbial plant biomass degraders

Sebastian G.A. Konietzny, Phillip B. Pope, Aaron Weimann, Alice C. McHardy

– Supplementary Note –

1. Pfam families with potential relevance for plant biomass degradation

Excluding most of the protein families with references in the CAZy database, the consensus modules M1 to M5 contained at least 20 Pfam families with rather unclear relationships to plant biomass degradation (see Table 3 of the main text). Examples of these are two 'domains of unknown function', DUF303 and DUF4008. Notably, DUF303 was a part of the hemicellulose-targeting gene cluster of *Fibrobacter succinogenes* (see section 6 below), and DUF4008 of module M5 seemed related to the CipA cellulosome scaffoldin protein of *Clostridium thermocellum*, as indicated by close genomic neighborhood (see discussion of PDM M5 in the main text). Further examples are 'fibronectin domains' (PF00041, PF14310), which are often observed as linker elements in modular cellulolytic enzymes [1], and a 'GDSL-like lipase/acylhydrolase' family (PF00657). The latter is an example of a family which is hard to assess at first sight, but is described as being involved in lignin degradation processes of white rot fungi [2]. Moreover, we discussed the 'ricin-type β-trefoil lectin' domains of module M2 in the main text, which could have functions for xylan-binding.

Many additional interesting Pfam and CAZy terms were associated with the PDMs, because they occurred in up to 8 corresponding modules of the 18 LDA runs (see the

numbers of their occurrences in the tables S1B-5B of Additional file 1). However, they were excluded from consideration due to the rather conservative construction of the consensus modules, which required a minimum of 9 occurrences. Despite the fact that these families occurred less frequently, they were clearly associated with the modules and represent further resources for the discovery of as yet unknown relationships among families. Note that in addition to the DUFs already contained in the PDMs, we identified 50 more DUFs that co-occur with module families within the identified gene clusters (18% of these were found in the 81 gene clusters of the phenotype-positive genomes).

2. Misclassified genomes of the learning set

Among the few genomes that were jointly misclassified by the modules M1 and M2 in our cross-validation experiments were *Bryantella formatexigens* (FN), *Xylanimonas cellulosilytica* (FN), *Thermonospora curvata* 43183 (FN) and *Actinosynnema mirum* (FP).

One reason for the misclassification of the first two species might be that both genomes were lacking annotations for GH9 (PF00759) (among other elements of M1). This cellulase family was described as an important component of the M1-associated *cip-cel* operon (see Figure 3A of the main text) and has essential cellulolytic activities in several organisms of our phenotype-positive set (see section 3 below). Based on this observation, it is likely that the reported activity of β-glucosidase in *B. formatexigens* [3] is mediated by families other than GH9, e.g. GH1, GH3, GH4 or GH5. Similarly, important elements of M2 – i.e. GH16, CBM13, CBM35, CBM61 and CBM47 – were missing in the annotations of the FN genomes.

In contrast to this, 'misclassification' of the other two species may be seen as a proof of concept. *A. mirum* and *T. curvata* were presumably correctly classified by the modules, whereas the phenotype characterization of the species in the original studies seems to have been wrong. According to a recent review on Actinobacteria [4], evidence of high

cellulase activity in *A. mirum* has been found, whereas *T. curvata* 43183 shows no cellulolytic activity and thus was probably characterized incorrectly in previous literature.

3. A discussion of the GH9 family and its role in lignocellulose-degrading species

The GH9 family is a large family of endoglucanases, some of which have been shown to act as processive cellulases on crystalline cellulose [5]. Despite the family's distribution across aerobic and anaerobic bacteria from a broad range of taxa, we found GH9 family annotations in only 313 isolate genomes and 22 metagenome bins, i.e. ~10% of our input collection. Remarkably, the GH9 family was annotated for 32 of the 38 phenotype-positive genomes, whereas it was almost absent in the phenotype-negative set, where it occurred in only 4 out of 82 genomes (see Additional file 7: Figures S1 and S2 (heat maps)). Thus GH9 was very specific for the known degraders.

The GH9 family has a key role in many cellulosomes [6], in which it is assumed to act in concert with other cellulosomal enzymes [7]. However, it has also been demonstrated to have cellulolytic activity in organisms of our phenotype-positive set that do not possess cellulosomes, such as *Caldicellulosiruptor bescii* [8] and *Thermobifida fusca* [9]. Furthermore, the cellulolytic activity of GH9 has been demonstrated for two more strains of species in our phenotype-positive set, *Clostridium phytofermentans* [10] and *Ruminoccus albus* 8 [11].

As described in the main text, GH9 occurred together with GH5 and CBM4 in the *cip-cel* operon of *Clostridium cellulolyticum* H10, for which it is known that the contained genes are involved in cellulose degradation [12]. Even more direct interactions of GH9 and GH5 families have been described. GH9 has been observed to be functionally linked with GH5 in a gene (Cel9B/Man5A) of *C. bescii* that encodes a highly thermophilic cellulase with potential application in biofuel production [8].

Concerning the reasons for the assignment of GH9 to module M1, we measured strong pairwise Pearson correlation of GH9 with the other elements across the learning set,

and identified gene clusters of GH9 and other M1 elements, such as GH10, GH43, CBM35, PF00756, PF02927, PF02018 and PF13472. Notably, in accordance with the known role of GH9 in cellulosomes, the GH9 family was also found to be associated with the cellulosome-related module M5 (the family was present in 7 out of 16 modules).

4. Characterization of the PDM M4

Many of the M4 protein families, such as GH2, GH3, GH5 and GH43, represent large protein families with a variety of possible functions. Members of GH3, GH5 and GH43 possess hemicellulose degradation activities (see Table 2 of the main text). Interestingly, the M4 module contained two different groups of structurally related protein families. These were β-galactosidase families (EC 3.2.1.23), like GH2, GH35 and GH42, and the three known members of the GH-D clan (a superfamily of α-galactosidases), i.e. GH27 (PF02065), GH31 and GH36. The GH31 family represents the most recent addition to this clan, based on structural and mechanistic similarities to GH27 and GH36 [13]. GH-D families have been studied mostly in the context of the degradation of galactoglucomannans (hemicelluloses) [13]. However, GH31 is also characterized as an α-xylosidase (EC 3.2.1.177) that catalyzes the hydrolysis of terminal xyloside residues at the extreme reducing end of xyloglucan-oligosaccharides.

To gain further insights into the possible functions of the M4 families, we investigated the gene clusters that were predicted by M4 in known lignocellulose degraders. Evidence for a link of M4 to xyloglucan degradation was found in the form of a long gene cluster of 8 genes (BACCELL_02066, BACCELL_02068–69 and BACCELL_02071–75) in the lignocellulose degrader *Bacteroides cellulosilyticus* DSM 14838, which was identified based on the protein family content of module M4. According to the gene annotations in IMG, the cluster comprises genes for 1 β-galactosidase, 2 α-glucosidases, 1 melibiase and 4 β-xylosidases. Three genes of the cluster were linked to the MetaCyc pathway of xyloglucan degradation based on their assigned EC numbers. In particular, EC number 3.2.1.23 of the GH2 family, which was annotated for the gene BACCELL_02066, mapped to a ‘xyloglucan oligosaccharide β-

galactosidase’ enzyme of the MetaCyc pathway. The M4 families that mapped to the gene cluster are: GH2, GH5, GH31, GH32, GH36, GH43 and PF02065 (melibiase/GH27).

We then searched for additional evidence of a common link between β-galactosidases and members of the GH-D clan. Larsbrink *et al*. recently proposed a model for xyloglucan utilization by *Cellvibrio japonicus* based on functional evidence [14], which comprises β-galactosidases, a member of the GH31 family and an endo-xyloglucanase enzyme (EC 3.2.1.151). In accordance with this, the GH5 family, which was also included in M4, contains members with endo-xyloglucanase activities [15]. Thus there is evidence for the participation of M4 families in xyloglucan degradation. It is also interesting to see the clustering of two groups of structurally related protein families in M4 (i.e. β-galactosidases and the GH-D clan), both of which have members with known activities in xyloglucan degradation.

5. Low abundance of GH6 and GH48 in our dataset

The families GH6 and GH48 play important roles in cellulose hydrolysis, but they are not universally present in known lignocellulose degraders. Their absence is known for *Fibrobacter succinogenes*, *Cytophaga hutchinsonii* and various gut/rumen metagenomes with lignocellulose-degrading capabilities [16-19]. As further examples, an absence of GH48 has been described for *Cellvibrio japonicus* [20] and *Saccharophagus degradans* [21]. According to our in-house CAZy/Pfam annotation sets, only 11/38 (29%) and 24/38 (63%) of the phenotype-positive genomes in our learning set contained GH6 or GH48, respectively.

We observed sparse co-occurrence signals for these two families. Both families were annotated for less than 5% of the 3,216 input documents. Only one protein-coding sequence of the analyzed metagenome bins was predicted to contain GH6 by our HMM-based annotation pipeline (none for GH48), i.e. both families were largely absent from the annotations of the metagenome bins. It is of course possible that some members of these families remained undetected if they had only remote sequence

homology to the family models of the dbCAN and Pfam databases that we used. Taupp *et al.* have described how GH6 could not be detected in the Tammar wallaby foregut metagenome by sequence analysis; however, it was later found in functional screens of fosmid libraries from the same source material [22]. Interestingly, our own annotation sets revealed a GH6-annotated gene sequence in a Clostridia-related bin of the Tammar wallaby forestomach microbiome.

Given these sparse and weak signals of GH6 and GH48, the LDA model performed well in placing the two GH families into a functional context, at least in the case of the GH48 family. Both families exhibited considerable associations (topic probabilities ≥0.005) to some of the inferred functional modules. Although the strength of these associations was less than that required by our rather strict threshold value (C = 0.01) for converting the topic probabilities to potential functional modules, further analyses showed that GH6 and GH48 were always placed among the top 50 protein families of their respective modules (typically at positions 30–35 in the list of families sorted by decreasing probability). We concluded that the sparseness of the co-occurrence signals was the main reason why both families had low probabilities in our topic model.

The modules with associations to GH6 did not match our stability criteria and we therefore did not report a consensus module for them. Apparently, LDA could not learn a stable clustering for GH6. In contrast to this, GH48 was weakly associated with the modules used for creating the M5 consensus module. M5 grouped cohesin and dockerin elements together and seemed to be related to the structural components of the cellulosome complex. A weak association to this particular module can be explained by the fact that many of the known bacterial cellulosomes contain proteins of the GH48 family [23]. However, members of the family GH48 also occur in organisms with multifunctional (*Caldicellulosiruptor saccharolyticus*) and free cellulolytic enzymes [24], e.g. in *Thermobifida fusca*. Therefore, GH48 is by no means exclusive to cellulosome complexes and so one should not expect a much stronger association with M5.

Concerning the role of GH48 in cellulosomes, the family was shown to be non-essential for the cellulose degradation abilities of *Clostridium thermocellum*. Olson *et al*. have created a Cel48S/Cel48Y double knockout-mutant of a *C. thermocellum* strain that

maintained its ability for solubilization of crystalline cellulose, albeit with a reduced rate compared to the wild type [25].

GH6 and GH48 did not correlate well with the elements of module M1 based on pairwise Pearson correlations, so it makes sense that they were not assigned into this module (see Additional file 7: Figures S1 and S2 (heat maps)).

6. Predicted occurrences of M1 in *Fibrobacter succinogenes* S85 and *Thermobifida fusca* YX

The protein families of M1 were organized in a large gene cluster in the genome of *Fibrobacter succinogenes* S85, which is centered on the gene FSU_2269 (note that in the ATCC 19169 strain that we analyzed, this corresponds to the gene Fisuc_1769). The cluster mainly contained xylanases composed of CBM6, CBM35, GH43 and DUF303 protein families, and has previously been described by Yoshida *et al*. [26, 27] (see Additional file 6: Figure S1). *F. succinogenes* is assumed to use an as yet uncharacterized degradation paradigm [28]. In contrast to this, *Thermobifida fusca* is one of the model organisms for the free enzyme strategy [29]. Almost 80% of the protein families in M1 were annotated in *T. fusca* YX, such that the organism was predicted to encode module M1 by our method; CBM4, CBM6, PF02018, GH5, GH9, GH10 and GH43 were among these. Although we found only very short gene clusters in this genome, this observation demonstrates how the module M1 spans organisms with different degradation paradigms. It is therefore likely that in organisms using the free enzyme strategy, such as *T. fusca,* the elements of M1 act alongside elements of other modules. As an example, we identified functional modules that were rich in 'type-II secretion system'-related Pfam families in many LDA runs, which, in principle, might be responsible for the secretion of cellulolytic enzymes. Type-II secretion systems for cellulases have been described previously, e.g. in the plant pathogen *Erwinia chrysanthemi* [30]. However, for *T. fusca* in particular, doubts about a type-II secretion process for secreting cellulolytic enzymes have been raised, based on missing homologs [29].

7. Predictions of the cellulosome-related PDM M5

In some species, as shown for the cellulosome-related gene clusters *cip-cel* and *xyl-doc* in *Clostridium cellulolyticum* H10, the protein families of M1 and M5 mixed together. It is a good result that LDA split the protein families involved into different modules, despite the co-occurrence patterns induced by such mixed gene clusters. However, the M5 module only covered part of the structural components of cellulosomes (cohesin and dockerin) and thus was a weak predictor for the cellulosome paradigm. For example, M5 was assigned to 4 out of 8 *Caldicellulosiruptor* species, though these species are assumed to employ the free enzyme strategy [31]. It has been described recently that cohesins and dockerins appear in many bacteria that have no cellulosomes, where they seem to mediate diverse functions [32]. Although this might explain some of the observations where M5 was predicted for non-cellulosomal organisms, it is not an explanation of why 4 of the 8 *Caldicellulosiruptor* species fulfilled the weight threshold condition for M5, as these organisms had neither cohesin nor dockerin annotations. Instead, they possessed annotations for other elements of M5, such as CBM3 (PF00942), CBM36, PF07591 (Pretoxin HINT domain), PF13186 (DUF4008), PF05593 (RHS repeat) and PF07238 (PilZ domain) (see Figure 4 of the main text; see Additional file 5: Table S5). These elements were grouped into M5 because they often co-occurred with the cohesin/dockerin families across the input collection (though not in gene clusters in general). Some of the predicted occurrences of M5 in non-cellulosomal organisms were due to the module's low completeness threshold (38.46%), which was the lowest for all PDMs, meaning that just a fraction of the member families of M5 needed to be present in a genome for a positive prediction. A similar case could be the M5 prediction for *Sorangium cellulosum*, because there were no cohesin or dockerin domains present in the genome. Finally, we would like to mention that unravelling the lignocellulolytic capabilities of *Caldicellulosiruptor* species is a topic of recent research, as these are thermophilic bacteria that have the potential to improve industrial biofuel production [8, 33].

8. Predicted occurrences of PDMs in metagenome bins

PDMs were mainly identified for the taxonomic bins of the orders Clostridiales and Bacteroidales (see Figure 5 of the main text). Species of these clades, together with species of the order Fibrobacter, have cellulolytic activities in microbial communities in the rumen and large intestine of mammalian species [34]. Naas *et al.* have described cellulose degradation enzymes in Bacteroidales-related genome assemblages reconstructed from the rumen microbiome of cows [35], which agrees well with the predictions of M1 to M4 for the Bacteroidales bins of this metagenome (see Table 5 of the main text). Predictions of the PDMs for Bacteroidales-affiliated bins of the foregut microbial community from the Tammar wallaby [36] and the reindeer rumen [19] could thus indicate similar capabilities in species from this order. Overall, only two metagenome bins in our dataset, from the termite hindgut metagenome and related fosmid sequences, represented the order Fibrobacter. These bins were annotated with only a few CAZy families, including GH9 and GH2, but not GH3, GH5, GH6, GH10, GH26, GH30, GH43, GH44 or GH48, which may be why there were no PDMs identified for these bins.

Interestingly, M1 and M4 were predicted to occur in the Treponema bins of the termite hindgut metagenome and the corresponding fosmid sequences. Species of this genus are involved in the degradation of cellulose and hemicellulose in the termite hindgut community [18]. Treponema species seem to be quite diverse in this respect, as no PDMs were identified for the six isolated *Treponema* species of our dataset. *Treponema bryantii*, which was not included in our dataset, is known to promote the degradation activities of cellulolytic species like *Fibrobacter succinogenes* but is itself likely non-cellulolytic [37, 38].

M4 and M5 were predicted for two Euryarchaeota (Archaea) bins of terephtalate-degrading microbial communities from a bioreactor. So far, only very few archaeal species that are capable of degrading lignocellulosic biomass are known; however, the extremophiles in particular have great potential to improve industrial biofuel production [39]. One of the predicted Euryarchaeota bins had an annotation for the cohesin family, while the other bin encoded 84% of the M4 families, including GH2, GH3 and GH5. For

this phylum, three isolate genomes were predicted by the PDMs. The genomes of *Halorhabdus utahensis* (M1, M2) and *Haloterrigena turkmenica* (M3, M4) both have large repertoires of glycoside hydrolases, and, in case of *H. turkmenica,* for pectin degradation as well [40]. *H. utahensis* grows on xylan and it was recently discovered that it possesses an active GH5 cellulase gene, with possible application for biofuel production due to its tolerance of extreme conditions during the pretreatment of biomass [40].

9. Predicted polysaccharide utilization loci (PULs) and Sus-like PUL systems

We identified two gene clusters (the genes BT4145–50 and BT4152–58) predicted by the pectin module M3 for *Bacteroides thetaiotaomicron*, which closely correspond to two genomic regions (BT4145–51 and BT4152–55, BT4158) that were characterized as being active in rhamnogalacturonan degradation in a PUL-targeted study [41]. Moreover, it is well known that some PULs contain SusD- and TonB-like membrane proteins. The Sus gene cluster was originally characterized as a membrane-bound degradation system for starch [42], but this hypothesis has since been generalized to include PULs targeting other polysaccharides and cellulose (Sus-like systems [35, 43, 44]). Our method did not assign the SusD or TonB protein families to any one of the highly ranked modules. However, in most of the 18 LDA runs, it consistently grouped these elements into potential functional modules of lower ranks which also incorporated some protein families that are involved in starch binding or known to be located at the outer membrane. The modules were stable and we included their consensus module (PUL module) in Additional file 1 (Table S6A). PULs are known to be involved in the degradation of various polysaccharides [44], particularly starch, which explains why the PUL module was not identified as being highly specific for lignocellulose degraders and thus obtained lower ranks in our module rankings. Because the PUL module contained elements of Sus-like systems but no glycoside hydrolases, we combined the protein families of this module with those of modules M1 and M2, and used this pooled family set to search for associated gene clusters in the combined prediction sets of the three modules. We found almost 300 gene clusters of four or more genes including *susD*

(PF07980), a TonB-dependent receptor domain (PF00593) and one or more genes annotated with the GH5, GH9, GH10 or GH43 family. Among these clusters, we identified a few Sus-like PULs from *Bacteroides ovatus* that have previously been characterized [41]. The PUL cluster BACOVA_02649–56 contained SusD, TonB, GH5, GH9 and GH43 annotations, and corresponds to a genomic region (BACOVA_02644–56) which has been characterized as targeting xyloglucan [41]. Similarly, SusD, TonB, GH10, GH30 and GH43 families were annotated in another predicted cluster (BACOVA_03424–33) that is embedded within a slightly longer genomic region, BACOVA_3421–36, which has been characterized as targeting oat spelt xylan and wheat arabinoxylan [41]. These well characterized clusters represent only a few examples from the overall ~300 gene clusters.

10. Gene clusters identified in the cow rumen bin AGa

The 15 draft genomes from the cow rumen metagenome were partially fragmented and not fully assembled [45]. The PDMs mapped to six gene clusters with four or more genes, and several shorter clusters in the draft genomes. We found an interesting large cluster on a 97,191-bp contig of the Bacteroidales-associated draft genome ‘AGa' (see Additional file 12: Figure S1), which was partly matched by the protein families of M1. The gene cluster (NODE_457020_ORF_01660 to NODE_457020_ORF_01710; protein sequences in Additional file 13) includes three cellulases, based on the assignments of the GH5 family, and a cellobiose phosphorylase (GH94; EC 2.4.1.20) with an attached putative CBM (PF06204). The GH94 family was not assigned to the consensus module of M1 but it was contained in the M1 modules in 7 out of 18 LDA runs. Depending on the presence or absence of GH94 in the M1 modules of different runs, the gene cluster was identified either partly or completely. The cluster was flanked by genes annotated with GH3 (gene 01650) and GH5 (01630) on the left-hand side of the cluster, and Pfam annotations that do not appear to be associated with lignocellulose degradation for the genes 01720 and 01730 on the right-hand side. The gene 01640 lies downstream of the identified gene cluster and was annotated with two members of the major facilitator superfamily (MFS), PF07690 and PF13347. The broadly defined major facilitator

superfamily has a variety of functions and includes proteins which are active in sugar uptake [46]. In particular, PF13347 is characterized as a 'MFS/sugar transport protein' in the Pfam database. As additional evidence, we observed that the pentose transporter gene BACOVA_04388, which is part of a conserved xylan hydrolase gene cluster in *Bacteroides ovatus* (described by Dodd *et al*. [47]), also contained a MFS annotation (PF13347) according to IMG. Based on this evidence, the predicted AGa gene cluster might be involved in the uptake of sugars. The gene 01720, which flanks the predicted cluster from the upstream side, includes the uncharacterized family PF13585. Blasting the protein sequence of 01720 yielded many hits for hypothetical proteins, but among the top hits, we found a 'C-type lectin domain-containing protein' from *Flavobacterium sp.* F52. Lectins are required for sugar binding [48]. Finally, BLAST searches for the genes 01680 and 01690, which are the uncharacterized candidate genes of interest (green box in Figure S1, Additional file 12), resulted in hits to a 'putative outer-membrane insertion C-signal' domain for gene 01680 and a 'partial iron chelating ABC transporter substrate' (binding) domain for gene 01690. In summary, we found evidence for functionally related genes in the vicinity of the cellulase genes in the cluster. Similar to some PUL systems, the cluster seems to encode proteins with catalytic activities targeting cellulose or hemicellulose, as well as proteins with functions located in the membrane, such as sugar binding or sugar transport.

11. Ranking results with two different choices of genome-specific module weights

The ranking depends on our definition of the 'genome-specific' module weights. We compared two different definitions of the weights. First of all, the LDA model already provides probabilities that we could use as weights. But, as outlined in the supplementary Methods (see Additional file 3: Section 2), these weights rely on assumptions that are not appropriate in our case. Indeed, we observed that the weights of modules may depend on the presence of other, more dominant modules in a genome and cannot be compared across genomes. In some cases, this resulted in high ranks for modules that were not relevant for the phenotype of interest. For our analyses, we

therefore used alternative weights based on the completeness of modules that measure the percentage of the elements of a module that can be found in a (meta-)genome.

Despite the drawbacks of the probability weights, the results obtained with the two different types of weights agreed well. This indicates the robustness of the ranking with respect to the specific choice of weights. Both approaches mostly placed the same module at the top rank and the modules M1 to M5 were generally among the top 15 of ranked modules (in the top 10 in most cases).

References

1. Sweeney MD, Xu F: **Biomass converting enzymes as industrial biocatalysts for fuels and chemicals: Recent developments.** *Catalysts* 2012, **2:**244-263.
2. Singh D, Zeng JJ, Laskar DD, Deobald L, Hiscox WC, Chen SL: **Investigation of wheat straw biodegradation by *Phanerochaete chrysosporium*.** *Biomass Bioenerg* 2011, **35:**1030-1040.
3. Wolin MJ, Miller TL, Collins MD, Lawson PA: **Formate-dependent growth and homoacetogenic fermentation by a bacterium from human feces: description of *Bryantella formatexigens gen. nov., sp. nov*.** *Appl Environ Microbiol* 2003, **69:**6321-6326.
4. Anderson I, Abt B, Lykidis A, Klenk HP, Kyrpides N, Ivanova N: **Genomics of aerobic cellulose utilization systems in actinobacteria.** *PLoS ONE* 2012, **7:**e39331.
5. Gilad R, Rabinovich L, Yaron S, Bayer EA, Lamed R, Gilbert HJ, Shoham Y: **CelI, a noncellulosomal family 9 enzyme from *Clostridium thermocellum*, is a processive endoglucanase that degrades crystalline cellulose.** *J Bacteriol* 2003, **185:**391-398.
6. Doi RH, Kosugi A: **Cellulosomes: plant-cell-wall-degrading enzyme complexes.** *Nat Rev Microbiol* 2004, **2:**541-551.
7. Gaudin C, Belaich A, Champ S, Belaich JP: **CelE, a multidomain cellulase from *Clostridium cellulolyticum*: a key enzyme in the cellulosome?** *J Bacteriol* 2000, **182:**1910-1915.
8. Su X, Mackie RI, Cann IK: **Biochemical and mutational analyses of a multidomain cellulase/mannanase from *Caldicellulosiruptor bescii*.** *Appl Environ Microbiol* 2012, **78:**2230-2240.
9. Sakon J, Irwin D, Wilson DB, Karplus PA: **Structure and mechanism of endo/exocellulase E4 from *Thermomonospora fusca*.** *Nat Struct Biol* 1997, **4:**810-818.
10. Tolonen AC, Chilaka AC, Church GM: **Targeted gene inactivation in *Clostridium phytofermentans* shows that cellulose degradation requires the family 9 hydrolase Cphy3367.** *Mol Microbiol* 2009, **74:**1300-1313.

11. Devillard E, Goodheart DB, Karnati SK, Bayer EA, Lamed R, Miron J, Nelson KE, Morrison M: ***Ruminococcus albus* 8 mutants defective in cellulose degradation are deficient in two processive endocellulases, Cel48A and Cel9B, both of which possess a novel modular architecture.** *J Bacteriol* 2004, **186:**136-145.
12. Blouzard J-C, Coutinho PM, Fierobe H-P, Henrissat B, Lignon S, Tardif C, Pagès S, de Philip P: **Modulation of cellulosome composition in *Clostridium cellulolyticum*: Adaptation to the polysaccharide environment revealed by proteomic and carbohydrate-active enzyme analyses.** *Proteomics* 2010, **10:**541-554.
13. Gilbert HJ, Stalbrand H, Brumer H: **How the walls come crumbling down: Recent structural biochemistry of plant polysaccharide degradation.** *Curr Opin Plant Biol* 2008, **11:**338-348.
14. Larsbrink J, Izumi A, Ibatullin FM, Nakhai A, Gilbert HJ, Davies GJ, Brumer H: **Structural and enzymatic characterization of a glycoside hydrolase family 31 α-xylosidase from Cellvibrio japonicus involved in xyloglucan saccharification.** *Biochem J* 2011, **436:**567-580.
15. Gloster TM, Ibatullin FM, Macauley K, Eklof JM, Roberts S, Turkenburg JP, Bjornvad ME, Jorgensen PL, Danielsen S, Johansen KS, et al: **Characterization and three-dimensional structures of two distinct bacterial xyloglucanases from families GH5 and GH12.** *J Biol Chem* 2007, **282:**19177-19189.
16. Wilson D: **Evidence for a novel mechanism of microbial cellulose degradation.** *Cellulose* 2009, **16:**723-727.
17. Duan C-J, Feng J-X: **Mining metagenomes for novel cellulase genes.** *Biotechnol Lett* 2010, **32:**1765-1775.
18. Warnecke F, Luginbuhl P, Ivanova N, Ghassemian M, Richardson TH, Stege JT, Cayouette M, McHardy AC, Djordjevic G, Aboushadi N, et al: **Metagenomic and functional analysis of hindgut microbiota of a wood-feeding higher termite.** *Nature* 2007, **450:**560-565.
19. Pope PB, Mackenzie AK, Gregor I, Smith W, Sundset MA, McHardy AC, Morrison M, Eijsink VG: **Metagenomics of the Svalbard reindeer rumen microbiome reveals abundance of polysaccharide utilization loci.** *PLoS ONE* 2012, **7:**e38571.
20. Deboy RT, Mongodin EF, Fouts DE, Tailford LE, Khouri H, Emerson JB, Mohamoud Y, Watkins K, Henrissat B, Gilbert HJ, Nelson KE: **Insights into plant cell wall degradation from the genome sequence of the soil bacterium *Cellvibrio japonicus*.** *J Bacteriol* 2008, **190:**5455-5463.
21. Taylor LE, 2nd, Henrissat B, Coutinho PM, Ekborg NA, Hutcheson SW, Weiner RM: **Complete cellulase system in the marine bacterium *Saccharophagus degradans* strain 2-40T.** *J Bacteriol* 2006, **188:**3849-3861.
22. Taupp M, Mewis K, Hallam SJ: **The art and design of functional metagenomic screens.** *Curr Opin Biotechnol* 2011, **22:**465-472.
23. Schwarz WH: **The cellulosome and cellulose degradation by anaerobic bacteria.** *Appl Microbiol Biotechnol* 2001, **56:**634-649.
24. Kumar M, Khanna S: **Shift in microbial population in response to crystalline cellulose degradation during enrichment with a semi-desert soil.** *Int Biodeterior Biodegradation* 2014, **88:**134-141.
25. Olson DG, Tripathi SA, Giannone RJ, Lo J, Caiazza NC, Hogsett DA, Hettich RL, Guss AM, Dubrovsky G, Lynd LR: **Deletion of the Cel48S cellulase from Clostridium thermocellum.** *Proc Natl Acad Sci U S A* 2010, **107:**17727-17732.
26. Yoshida S, Hespen CW, Beverly RL, Mackie RI, Cann IK: **Domain analysis of a modular α-L-arabinofuranosidase with a unique carbohydrate binding strategy from the fiber-degrading bacterium *Fibrobacter succinogenes S85*.** *J Bacteriol* 2010, **192:**5424-5436.

27. Yoshida S, Mackie RI, Cann IK: **Biochemical and domain analyses of *FSUAxe6B*, a modular acetyl xylan esterase, identify a unique carbohydrate binding module in *Fibrobacter succinogenes S85*.** *J Bacteriol* 2010, **192:**483-493.
28. Wilson DB: **Three microbial strategies for plant cell wall degradation.** *Ann N Y Acad Sci* 2008, **1125:**289-297.
29. Lykidis A, Mavromatis K, Ivanova N, Anderson I, Land M, DiBartolo G, Martinez M, Lapidus A, Lucas S, Copeland A, et al: **Genome sequence and analysis of the soil cellulolytic actinomycete *Thermobifida fusca YX*.** *J Bacteriol* 2007, **189:**2477-2486.
30. Chapon V, Czjzek M, El Hassouni M, Py B, Juy M, Barras F: **Type II protein secretion in gram-negative pathogenic bacteria: The study of the structure/secretion relationships of the *cellulase Cel5* (formerly EGZ) from *Erwinia chrysanthemi*.** *J Mol Biol* 2001, **310:**1055-1066.
31. Blumer-Schuette SE, Lewis DL, Kelly RM: **Phylogenetic, microbiological, and glycoside hydrolase diversities within the extremely thermophilic, plant biomass-degrading genus *Caldicellulosiruptor*.** *Appl Environ Microbiol* 2010, **76:**8084-8092.
32. Peer A, Smith SP, Bayer EA, Lamed R, Borovok I: **Noncellulosomal cohesin- and dockerin-like modules in the three domains of life.** *FEMS Microbiol Lett* 2009, **291:**1-16.
33. Blumer-Schuette SE, Giannone RJ, Zurawski JV, Ozdemir I, Ma Q, Yin Y, Xu Y, Kataeva I, Poole FL, 2nd, Adams MW, et al: ***Caldicellulosiruptor* core and pangenomes reveal determinants for noncellulosomal thermophilic deconstruction of plant biomass.** *J Bacteriol* 2012, **194:**4015-4028.
34. Flint HJ, Bayer EA, Rincon MT, Lamed R, White BA: **Polysaccharide utilization by gut bacteria: Potential for new insights from genomic analysis.** *Nat Rev Microbiol* 2008, **6:**121-131.
35. Naas AE, Mackenzie AK, J. M, Schückel J, Willats WGT, Eijsink VGH, Pope PB: **Do rumen Bacteroidetes utilize an alternative mechanism for cellulose degradation?** *mBio* 2014, **5:**e01401-01414.
36. Pope PB, Denman SE, Jones M, Tringe SG, Barry K, Malfatti SA, McHardy AC, Cheng JF, Hugenholtz P, McSweeney CS, Morrison M: **Adaptation to herbivory by the Tammar wallaby includes bacterial and glycoside hydrolase profiles different from other herbivores.** *Proc Natl Acad Sci U S A* 2010, **107:**14793-14798.
37. Kudo H, Cheng KJ, Costerton JW: **Interactions between *Treponema bryantii* and cellulolytic bacteria in the in vitro degradation of straw cellulose.** *Can J Microbiol* 1987, **33:**244-248.
38. Nouaille R, Matulova M, Delort AM, Forano E: **Oligosaccharide synthesis in *Fibrobacter succinogenes* S85 and its modulation by the substrate.** *FEBS J* 2005, **272:**2416-2427.
39. Graham JE, Clark ME, Nadler DC, Huffer S, Chokhawala HA, Rowland SE, Blanch HW, Clark DS, Robb FT: **Identification and characterization of a multidomain hyperthermophilic cellulase from an archaeal enrichment.** *Nat Commun* 2011, **2:**375.
40. Anderson I, Scheuner C, Göker M, Mavromatis K, Hooper SD, Porat I, Klenk H-P, Ivanova N, Kyrpides N: **Novel insights into the diversity of catabolic metabolism from ten haloarchaeal genomes.** *PLoS ONE* 2011, **6:**e20237.
41. Martens EC, Lowe EC, Chiang H, Pudlo NA, Wu M, McNulty NP, Abbott DW, Henrissat B, Gilbert HJ, Bolam DN, Gordon JI: **Recognition and degradation of plant cell wall polysaccharides by two human gut symbionts.** *PLoS Biol* 2011, **9:**e1001221.
42. Reeves AR, Wang GR, Salyers AA: **Characterization of four outer membrane proteins that play a role in utilization of starch by *Bacteroides thetaiotaomicron*.** *J Bacteriol* 1997, **179:**643-649.
43. Mackenzie AK, Pope PB, Pedersen HL, Gupta R, Morrison M, Willats WG, Eijsink VG: **Two SusD-like proteins encoded within a polysaccharide utilization locus of an uncultured ruminant bacteroidetes phylotype bind strongly to cellulose.** *Appl Environ Microbiol* 2012, **78:**5935-5937.

44. Martens EC, Koropatkin NM, Smith TJ, Gordon JI: **Complex glycan catabolism by the human gut microbiota: The Bacteroidetes Sus-like paradigm.** *J Biol Chem* 2009, **284:**24673-24677.
45. Hess M, Sczyrba A, Egan R, Kim TW, Chokhawala H, Schroth G, Luo S, Clark DS, Chen F, Zhang T, et al: **Metagenomic discovery of biomass-degrading genes and genomes from cow rumen.** *Science* 2011, **331:**463-467.
46. Pao SS, Paulsen IT, Saier MH, Jr.: **Major facilitator superfamily.** *Microbiol Mol Biol Rev* 1998, **62:**1-34.
47. Dodd D, Mackie RI, Cann IKO: **Xylan degradation, a metabolic property shared by rumen and human colonic bacteroidetes.** *Mol Microbiol* 2011, **79:**292-304.
48. Boraston AB, Tomme P, Amandoron EA, Kilburn DG: **A novel mechanism of xylan binding by a lectin-like module from *Streptomyces lividans* xylanase 10A.** *Biochem J* 2000, **350 Pt 3:**933-941.
