# Supplementary material for: Inference of phenotype-defining functional modules of protein families for microbial plant biomass degraders
Source: Biotechnol Biofuels. 2014 Sep 9;7:124. doi: 10.1186/s13068-014-0124-8 (PMC4189754; doi:10.1186/s13068-014-0124-8)
Supplement: Additional file 3: — Supplementary Methods. Additional details about the methods and preparation of the input data. [file 13068_2014_124_MOESM3_ESM.pdf]

Inference of phenotype-defining functional modules of protein families for microbial plant biomass degraders

Sebastian G.A. Konietzny, Phillip B. Pope, Aaron Weimann, Alice C. McHardy

– Supplementary Methods –

1. Preparing the input for LDA

From the set of protein family annotations for the (meta-)genomes, a suitable input collection for LDA was created. We limited our consideration to metagenomic sequences with taxonomic assignments because our aim was to process individual taxonomic bins, rather than whole metagenome samples. This strategy of splitting large metagenome samples into multiple taxonomic bins sharpened the co-occurrence signals, as it increased the number of input documents for LDA while reducing the effective sizes of the corresponding documents.

Protein families occurring in more than 75% of the genomes/taxonomic bins were removed from the annotation set, as these tend to be uninformative. To reduce the impact of very abundant families (e.g. those found by unspecific HMMs), the maximum number of hits considered for any family per genome or taxonomic bin was limited to 10, even if we found more than 10 hits for this particular family.

After filtering, for each genome and taxonomic bin, we defined a ‘document’ as the list of all protein families present in the corresponding annotation set. We therefore treated

protein family identifiers (e.g. GH16, PF00150) as equivalents to words in a natural language in text documents, and used these identifiers to define the input vocabulary V for LDA. Each created document can be seen as a multiset of protein families, meaning that single families may occur more than once. The resulting vocabulary comprised 8,413 family identifiers, 8,141 Pfam-A terms and 272 CAZy/dbCAN terms. In summary, we created an input collection of 3,216 documents, representing 2,884 prokaryotic genomes and 332 taxonomic bins from 18 metagenomes.

2. Two definitions of genome-specific module weights

We tested two definitions of weights: The probability weights $\theta_d(t)$ of the LDA model and 'completeness scores'.

In the generative LDA model, $\theta_d(t) = P(t \mid d)$ describes the probability that a newly sampled word for document d originates from topic t. Thus the word content of a document is most likely shaped by the topics with high probability values $\theta_d(t)$, and $\theta_d(t)$ reflects the relative importance of a topic.

It might appear intuitive by analogy to use the inferred $\theta_d(t)$ probabilities of topics to estimate the importance of modules for (meta-)genomes in the context of the phenotype prediction problem. This would correspond to setting the weight matrix W to be equivalent to the matrix Θ of the inferred LDA model, where single values are computed according to the formula:

$$\theta_t^d = \frac{n_t^{(d)} + \alpha}{\sum_{k=1}^{T} n_k^{(d)} + T\alpha} = \frac{n_t^{(d)} + \alpha}{|d| + T\alpha}$$

where $n_t^{(d)}$ is the number of words in document d that were assigned to topic t after the Gibbs sampling, T is the number of topics, and α is one of the two Dirichlet hyperparameters of the LDA model. However, one potential problem is that the values

reflect the relative amount of words that were assigned to the respective topic, which means the values are relative with respect to the importance of other topics that compete for the word assignments in the same document. Thus if more dominant topics exist in a document, they are likely to decrease the weights of all the other topics. This effect could be problematic when we compare the weights of a particular functional module in two different (meta-)genomes. The dominant topics of the individual documents are usually different from each other and also have varying relevance for the particular phenotype of interest. In both cases, the result is a document-specific shift of the weights of the non-dominant topics, which, in some cases, might hinder unbiased comparisons between different documents. We therefore aimed to define an intuitive and direct measure for the presence of a module in a (meta-)genome that does not depend on the document size or on other modules. The 'completeness score' of a module is the percentage of a module's protein families that occur in a specific genome or taxonomic bin. More precisely, we defined the weight of a module M_t in document d of the (meta-)genome collection based on completeness as:

$$weight_t(d) := \frac{|M_t \cap d|}{|M_t|} \times 100\%,$$

where $|M_t \cap d|$ is the size of the intersection of the protein family sets of module M_t and document d, and $|M_t|$ is the number of protein families contained in M_t. Note that the thresholds for these weights are more intuitive than those for weights based on the probability values $\theta_d(t)$.

3. The F-measure

The F-measure [1] is a combined measure of precision and recall, and is commonly used for assessing the performance in classification tasks [2] and for attribute ranking [3-5]. Since the process of lignocellulose degradation can be decomposed into different paradigms, we are searching for functional modules that are exclusive to some (but not all) of the lignocellulose degraders. Therefore, we should not require a module to

achieve perfect recall, although we are generally interested in modules that are specific to lignocellulose degraders. The F-measure is the weighted harmonic mean of precision and recall, where the weight factor β controls the relative importance of precision (P) and recall (R) [6]:

$$F_\beta = \frac{(\beta^2 + 1)PR}{\beta^2 P + R}$$

Setting $\beta = 1.0$ gives equal importance to precision and recall, and F_β reduces to F. We used $\beta = 0.5$ to give more importance to precision than to recall (i.e. recall half as important as precision) as suggested by Lewis [7].

4. Identification of similar modules across runs using Bron-Kerbosch

The Bron–Kerbosch algorithm [8] operates on an undirected graph and detects all maximal cliques, i.e. all complete subgraphs that cannot be extended by edges to additional vertices of the graph without losing their complete connectivity. In a complete subgraph, any two vertices are connected by an edge.

We constructed the input graph for the Bron-Kerbosch algorithm by inserting edges that represented the pairwise optimal mappings between the modules of any two LDA runs, as identified by the Hungarian algorithm [9]. Depending on the choice of the pairwise distance measure, we required KL-distances ≤ 5 or Jaccard distances ≤ 0.75 for the inclusion of edges. Cliques in this graph, found by the Bron-Kerbosch algorithm, represent sets of functional modules from different LDA runs with highly similar protein family content, as it holds true for all modules of a clique that they were identified as being their pairwise best matches by the Hungarian algorithm.

For the high-ranking modules, we found overlapping cliques of sizes ≥ 9, which shared one or more modules. Such overlaps establish (transitive) similarities between the modules of the overlapping cliques. Therefore, we merged the module sets of

overlapping cliques to define the set of functional modules used for generating a consensus module.

5. LDA model stability

Model inference with LDA depends on Gibbs sampling and needs to be monitored for successful convergence. We monitored the progression of the likelihood to assess the convergence of the Gibbs sampling procedure and specified 2000 iterations as the burn-in phase of a run, though a tendency for convergence could be observed much earlier. After burn-in, we collected 50 Gibbs samples from the posterior distribution over the model's parameters by taking a sample after every 10 iterations. Each sample was defined by an instance of the model's $\vec{z}$ vector, and we computed the distributions $\phi_t(w)$ and $\theta_d(t)$ for all documents and topics by averaging over the 50 samples. In general, averaging over samples allows us to summarize information from Gibbs samples [10, 11], but in the case of LDA, there is a theoretical risk of topic identity switching, even between samples from the same Markov chain [12]. We therefore used pairwise KL distances to track topics across the 50 samples and found no evidence for switching.

References

1. Van Rijsbergen CJ: *Information retrieval.* 2nd edn. London ; Boston: Butterworths; 1979.
2. Witten IH, Frank E: *Data mining: Practical machine learning tools and techniques, second edition (Morgan Kaufmann series in data management systems)* San Francisco, USA: Morgan Kaufmann Publishers Inc.; 2005.
3. Wang Z, Willard HF: **Evidence for sequence biases associated with patterns of histone methylation.** *BMC Genomics* 2012, **13:**367.
4. Akay MF: **Support vector machines combined with feature selection for breast cancer diagnosis.** *Expert Systems with Applications* 2009, **36:**3240-3247.
5. Chen YK, Li KB: **Predicting membrane protein types by incorporating protein topology, domains, signal peptides, and physicochemical properties into the general form of Chou's pseudo amino acid composition.** *J Theor Biol* 2013, **318:**1-12.
6. Shatkay H, Feldman R: **Mining the biomedical literature in the genomic era: an overview.** *J Comput Biol* 2003, **10:**821-855.

7. Lewis DD: **Evaluating and optimizing autonomous text classification systems.** *Proceedings of the Eighteenth Annual International ACM SIGIR Conference on Research and Development in Information Retrieval* 1995**:**246-254.
8. Bron C, Kerbosch J: **Algorithm 457: Finding all cliques of an undirected graph.** *Commun ACM* 1973, **16:**575-577.
9. Kuhn HW: **The Hungarian method for the assignment problem.** *Nav Res Log* 1955, **2:**83-97.
10. Gilks WR, Richardson S, Spiegelhalter DJ: *Markov Chain Monte Carlo in Practice* Boca Raton, Florida, USA: Chapman and Hall/CRC; 1999.
11. Heinrich G: *Technical report: Parameter estimation for text analysis* Darmstadt, Germany: Fraunhofer IGD; 2009.
12. Griffiths TL, Steyvers M: **Finding scientific topics.** *Proc Natl Acad Sci U S A* 2004, **101 Suppl 1:**5228-5235.
