# Supplementary material for: Inference of phenotype-defining functional modules of protein families for microbial plant biomass degraders
Source: Biotechnol Biofuels. 2014 Sep 9;7:124. doi: 10.1186/s13068-014-0124-8 (PMC4189754; doi:10.1186/s13068-014-0124-8)
Supplement: Additional file 4: — Plant biomass degradation module (PDM) assignments to the genomes of the learning set. PDM assignments to the genomes of known plant biomass degraders and non-degraders are visualized in Figure S1, and were obtained by leave-one-out classification. [file 13068_2014_124_MOESM4_ESM.pdf]

Figure S1

IMG OID	M1	M2	M3	M4	M5	Predicted saprophyte	Cellulosome	Organism name	Phylum	Oxygen requirement
648276600						yes	yes	Acetivibrio cellulolyticus CD2, DSM 1870	Firmicutes	?
639633001						yes	?	Acidothermus cellulolyticus 11B	Actinobacteria	Aerobe
648028006						yes	?	Amycolatopsis mediterranei U32	Actinobacteria	?
643886111						yes	?	Bacteroides cellulosilyticus DSM 14838	Bacteroidetes	Anaerobe
643886107						yes	?	Bryantella formatexigens I-52, DSM 14469	Firmicutes	Anaerobe
650377919						yes	?	Butyrivibrio fibrisolvens 16/4	Firmicutes	Anaerobe
643692002						yes	?	Caldicellulosiruptor bescii Z-1320, DSM 6725	Firmicutes	Obligate anaerobe
649633022						yes	?	Caldicellulosiruptor hydrothermalis 108	Firmicutes	Anaerobe
649633023						yes	?	Caldicellulosiruptor kristjanssonii 177R1B, DSM 12137	Firmicutes	Anaerobe
649633024						yes	?	Caldicellulosiruptor kronotskyensis 2002	Firmicutes	Anaerobe
649989918						yes	?	Caldicellulosiruptor lactoaceticus 6A, DSM 9545	Firmicutes	Anaerobe
648028013						yes	?	Caldicellulosiruptor obsidiansis OB47	Firmicutes	Anaerobe
649633025						yes	?	Caldicellulosiruptor owensensis OL	Firmicutes	Anaerobe
640427106						yes	?	Caldicellulosiruptor saccharolyticus DSM 8903	Firmicutes	Anaerobe
646564520						yes	?	Cellulomonas flavigena 134, DSM 20109	Actinobacteria	Facultative
647000229						yes	?	Cellulosilyticum lentocellum RHM5, DSM 5427	Firmicutes	?
642555119						yes	?	Cellvibrio japonicus Ueda107	Proteobacteria	Aerobe
637000076						yes	yes	Clostridium acetobutylicum ATCC 824	Firmicutes	Obligate anaerobe
643348528						yes	yes	Clostridium cellulolyticum H10	Firmicutes	Anaerobe
648028016						yes	yes	Clostridium cellulovorans 743B, ATCC 35296	Firmicutes	Anaerobe
645058763						yes	yes	Clostridium papyrosolvens DSM 2782	Firmicutes	Anaerobe
641228486						yes	?	Clostridium phytofermentans ISDg	Firmicutes	Anaerobe
640069309						yes	yes	Clostridium thermocellum ATCC 27405	Firmicutes	Anaerobe
637000087						yes	?	Cytophaga hutchinsonii ATCC 33406	Bacteroidetes	Aerobe
643348543						yes	?	Dictyoglomus turgidum DSM 6724	Dictyoglomi	Anaerobe
649989949						yes	?	Eubacterium cellulosolvens 6	Firmicutes	?
646311927						yes	?	Fibrobacter succinogenes subsp. succinogenes S85	Fibrobacteres	Anaerobe
649633094						yes	yes	Ruminococcus albus 7	Firmicutes	Anaerobe
647000309						yes	yes	Ruminococcus albus 8	Firmicutes	Anaerobe
645951829						yes	yes	Ruminococcus flavefaciens FD-1	Firmicutes	Anaerobe
637000249						yes	?	Saccharophagus degradans 2-40	Proteobacteria	Aerobe
641228509						yes	?	Sorangium cellulosum So ce 56	Proteobacteria	Aerobe
648028053						yes	?	Spirochaeta thermophila DSM 6192	Spirochaetes	Anaerobe
645058856						yes	?	Streptomyces lividans TK24	Actinobacteria	Aerobe
644736410						yes	?	Teredinibacter turnerae T7901	Proteobacteria	?
637000319						yes	?	Thermobifida fusca YX	Actinobacteria	Aerobe
646311968						yes	?	Xylanimonas cellulosilytica DSM 15894	Actinobacteria	Aerobe
644736323						yes	?	Actinosynnema mirum 101, DSM 43827	Actinobacteria	Aerobe
639633005						yes	?	Arthrobacter aurescens TC1	Actinobacteria	Aerobe
641228485						yes	?	Chloroflexus aurantiacus J-10-fl	Chloroflexi	Anaerobe
643348536						yes	?	Desulfatibacillum alkenivorans AK-01	Proteobacteria	Anaerobe
643348560						yes	?	Klebsiella pneumoniae 342	Proteobacteria	Facultative
642555144						yes	?	Nostoc punctiforme PCC 73102	Cyanobacteria	Aerobe
640427137						yes	?	Rhizobium etli CFN 42, DSM 11541	Proteobacteria	Aerobe
641228511						yes	?	Thermotoga lettingae TMO	Thermotogae	Anaerobe
646311963						yes	?	Thermomonospora curvata DSM 43183	Actinobacteria	Aerobe
644736321						no	?	Acetobacter pasteurianus IFO 3283-01	Proteobacteria	Aerobe
644736322						no	?	Acidimicrobium ferrooxidans ICP, DSM 10331	Actinobacteria	Aerobe
643348501						no	?	Acidithiobacillus ferrooxidans ATCC 23270	Proteobacteria	Obligate aerobe
643348541						no	?	Acidovorax ebreus TPSY	Proteobacteria	Facultative
639279301						yes	?	Agrobacterium tumefaciens C58-Cereon	Proteobacteria	Aerobe
637000004						no	?	Alcanivorax borkumensis SK2	Proteobacteria	Aerobe
637000005						no	?	Alkalilimnicola ehrlichei MLHE-1	Proteobacteria	Anaerobe
640753002						yes	?	Alkaliphilus metalliredigens QYMF	Firmicutes	Anaerobe
638154502						no	?	Archaeoglobus fulgidus VC-16, DSM 4304	Euryarchaeota	Anaerobe
639633007						no	?	Azoarcus sp. BH72	Proteobacteria	Facultative
641228476						no	?	Azorhizobium caulinodans ORS 571	Proteobacteria	?
643692004						yes	?	Azotobacter vinelandii DJ, ATCC BAA-1303	Proteobacteria	Aerobe
641522606						yes	?	Beijerinckia indica indica ATCC 9039	Proteobacteria	Aerobe
642555114						no	?	Candidatus Amoebophilus asiaticus 5a2	Bacteroidetes	?
637000074						no	?	Chromobacterium violaceum ATCC 12472	Proteobacteria	Facultative
645058871						no	?	Comamonas testosteroni KF-1	Proteobacteria	Aerobe
644736347						yes	?	Cupriavidus taiwanensis LMG 19424	Proteobacteria	Facultative
641522622						no	?	Cyanothece sp. BH68, ATCC 51142	Cyanobacteria	Facultative
637000089						no	?	Dehalococcoides ethenogenes 195	Chloroflexi	Anaerobe
643348537						no	?	Desulfitobacterium hafniense DCB-2	Firmicutes	Anaerobe
644736349						no	?	Desulfohalobium retbaense HR100, DSM 5692	Proteobacteria	Obligate anaerobe
644736350						no	?	Desulfomicrobium baculatum X, DSM 4028	Proteobacteria	Obligate anaerobe
637000094						no	?	Desulfotalea psychrophila LSv54	Proteobacteria	Anaerobe
640069310						no	?	Desulfotomaculum reducens MI-1	Firmicutes	Anaerobe
640753051						yes	?	Ensifer medicae WSM419	Proteobacteria	Aerobe
637000115						yes	?	Frankia alni ACN14a	Actinobacteria	Aerobe
642555129						no	?	Geobacter bemidjiensis Bem, DSM 16622	Proteobacteria	Anaerobe
640069314						no	?	Hyperthermus butylicus DSM 5456	Crenarchaeota	Anaerobe
637000143						no	?	Lactobacillus salivarius salivarius UCC118	Firmicutes	Facultative
639633036						no	?	Magnetococcus sp. MC-1	Proteobacteria	Facultative
639633037						no	?	Marinobacter aquaeolei VT8	Proteobacteria	Facultative
637000159						yes	?	Mesorhizobium loti MAFF303099	Proteobacteria	Aerobe
640427120						no	?	Metallosphaera sedula DSM 5348	Crenarchaeota	Aerobe
640427121						no	?	Methanobrevibacter smithii PS, ATCC 35061	Euryarchaeota	Anaerobe
644736385						no	?	Methanocaldococcus fervens AG86	Euryarchaeota	Anaerobe
637000161						no	?	Methanococcoides burtonii DSM 6242	Euryarchaeota	Anaerobe
640069317						no	?	Methanocorpusculum labreanum Z	Euryarchaeota	Anaerobe
640069318						no	?	Methanoculleus marisnigri JR1, DSM 1498	Euryarchaeota	Anaerobe
638154507						no	?	Methanopyrus kandleri AV19	Euryarchaeota	Anaerobe
638154508						no	?	Methanosarcina acetivorans C2A	Euryarchaeota	Anaerobe
637000163						no	?	Methanosphaera stadtmanae DSM 3091	Euryarchaeota	Anaerobe
640069319						no	?	Methylibium petroleiphilum PM1	Proteobacteria	Facultative
643348565						no	?	Methylocella silvestris BL2, DSM 15510	Proteobacteria	Aerobe
643692029						no	?	Nautilia profundicola Am-H	Proteobacteria	Obligate anaerobe
637000192						no	?	Nitrobacter hamburgensis X14	Proteobacteria	Aerobe
637000194						no	?	Nitrosococcus oceani C-107, ATCC 19707	Proteobacteria	Aerobe
637000195						no	?	Nitrosomonas europaea ATCC 19718	Proteobacteria	Aerobe
641228499						no	?	Nitrosopumilus maritimus SCM1	Thaumarchaeota	Aerobe
637000197						no	?	Nitrosospira multiformis ATCC 25196	Proteobacteria	Aerobe
639633048						no	?	Paracoccus denitrificans PD1222	Proteobacteria	Aerobe
640753040						no	?	Parvibaculum lavamentivorans DS-1	Proteobacteria	Aerobe
640427128						no	?	Pelotomaculum thermopropionicum SI	Firmicutes	Anaerobe
643692030						no	?	Persephonella marina EX-H1	Aquificae	Microaerophilic
639633051						no	?	Polaromonas naphthalenivorans CJ2	Proteobacteria	Aerobe
637000310						no	?	Prochlorococcus sp. CC9605	Cyanobacteria	?
640427131						no	?	Pseudomonas mendocina ymp	Proteobacteria	Aerobe
638154513						no	?	Pyrobaculum aerophilum IM2	Crenarchaeota	?
638154514						no	?	Pyrococcus abyssi GE5	Euryarchaeota	Anaerobe
643348570						yes	?	Rhodobacter sphaeroides KD131	Proteobacteria	Facultative
637000234						yes	?	Rhodococcus jostii RHA1	Actinobacteria	Aerobe
637000235						no	?	Rhodoferax ferrireducens T118	Proteobacteria	Anaerobe
637000241						no	?	Rhodospirillum rubrum S1, ATCC 11170	Proteobacteria	Facultative
644736405						no	?	Slackia heliotrinireducens RHS 1, DSM 20476	Actinobacteria	Obligate anaerobe
639633062						no	?	Streptococcus thermophilus LMD-9	Firmicutes	Facultative
638154517						no	?	Sulfolobus acidocaldarius DSM 639	Crenarchaeota	Aerobe
637000326						no	?	Sulfurimonas denitrificans DSM 1251	Proteobacteria	Anaerobe
646311960						no	?	Sulfurospirillum deleyianum DSM 6946	Proteobacteria	Microaerophilic
637000308						no	?	Synechococcus elongatus PCC 7942	Cyanobacteria	Facultative
637000316						no	?	Syntrophomonas wolfei Goettingen, DSM 2245B	Firmicutes	Anaerobe
637000317						no	?	Syntrophus aciditrophicus SB	Proteobacteria	Anaerobe
643348585						no	?	Thioalkalivibrio sp. HL-EbGR7	Proteobacteria	Facultative
637000324						no	?	Thiobacillus denitrificans ATCC 25259	Proteobacteria	Facultative
637000325						no	?	Thiomicrospira crunogena XCL-2	Proteobacteria	Anaerobe
637000356						no	?	Zymomonas mobilis mobilis ZM4	Proteobacteria	Facultative
