# Supplementary figures and images for: Inference of phenotype-defining functional modules of protein families for microbial plant biomass degraders
Source: Biotechnol Biofuels. 2014 Sep 9;7:124. doi: 10.1186/s13068-014-0124-8 (PMC4189754; doi:10.1186/s13068-014-0124-8)

Figure S1

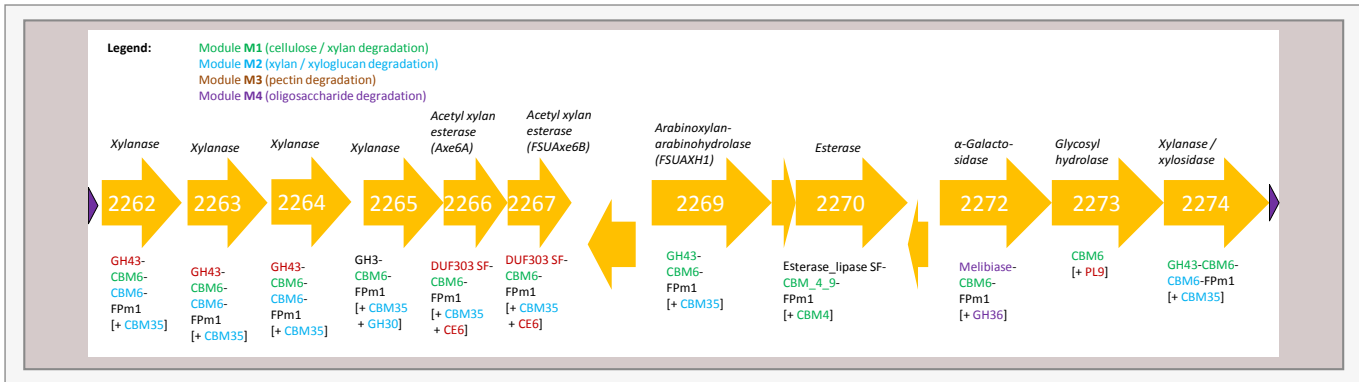

Supplement: Additional file 6: — Hemicellulolytic gene cluster in Fibrobacter succinogenes S85. The gene cluster in Figure S1 encodes more than 10 hemicellulose-targeting enzymes in the genome of F. succinogenes S85. The protein domain architecture of the cluster genes has been described by Yoshida et al. [63,64]. F. succinogenes does not use a cellulosome-based degradation strategy, but rather a degradation paradigm that is still uncharacterized [92,93]. [file 13068_2014_124_MOESM6_ESM.pdf]

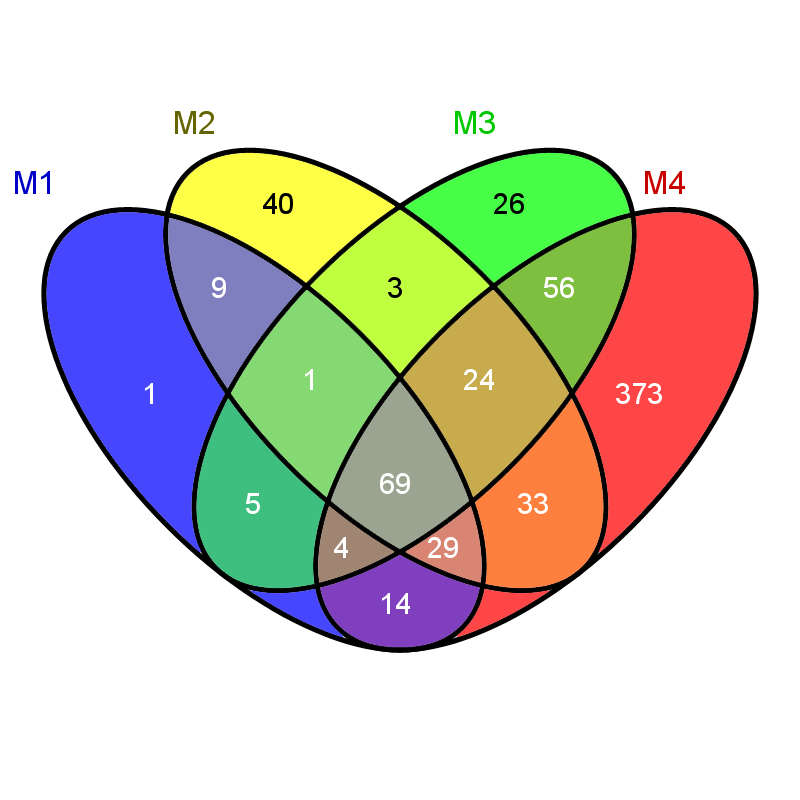

Supplement: Additional file 11: — Venn diagram of the predicted occurrences of the modules M1 to M4. The diagram displays the overlap between the genomes and metagenome bins with predicted occurrences of the modules M1, M2, M3, and M4. Genomes from the learning set were excluded. [file 13068_2014_124_MOESM11_ESM.png]

Figure S1

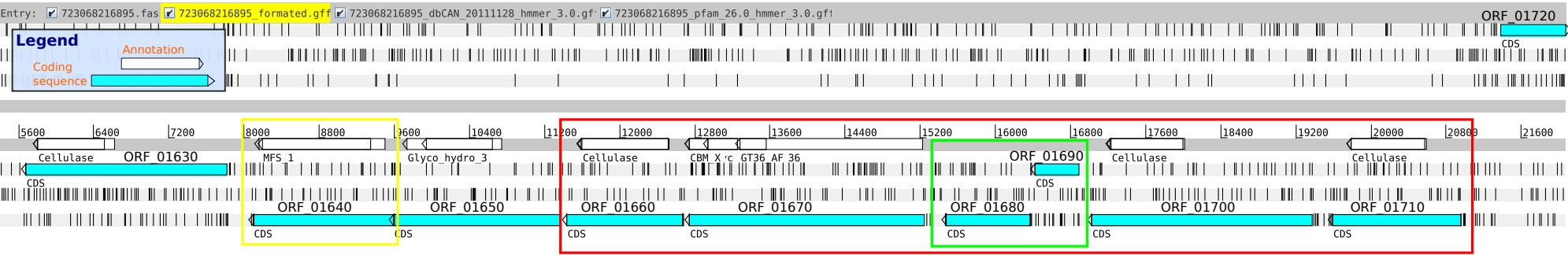

Supplement: Additional file 12: — Gene cluster in the cow rumen draft genome AGa. The red box in Figure S1 marks a gene cluster (NODE_457020_ORF_01660 to NODE_457020_ORF_01710), which was identified based on the families assigned to highest scoring module (M1). The cluster is located on a 97,191-bp contig of the draft genome AGa (Bacteroidales) from the cow rumen metagenome [19]. The cluster includes three cellulases, based on assignments of the GH5 family, and a cellobiose phosphorylase (GH94; EC 2.4.1.20) with an attached putative carbohydrate binding domain (PF06204). The GH94 family was not assigned to the consensus module of M1, but it was contained in the M1 modules in 7 of 18 LDA runs. Depending on the presence or absence of GH94 in the M1 modules of different runs, the gene cluster was identified either partly or completely. The cluster includes two genes (genes 01680 and 01690; green rectangle) without any annotated functional domains; these are uncharacterized genes that may be relevant for the degradation of lignocellulose. The presence of two Pfam families related to the major facilitator superfamily in gene 01640 (marked by the yellow box) indicates a link between the (hemi)cellulases of the GH5 and GH94 families, and sugar-binding or transport proteins located in the outer membrane (see Additional file 2: Section 10). [file 13068_2014_124_MOESM12_ESM.pdf]
