# Supplementary material for: Inference of phenotype-defining functional modules of protein families for microbial plant biomass degraders
Source: Biotechnol Biofuels. 2014 Sep 9;7:124. doi: 10.1186/s13068-014-0124-8 (PMC4189754; doi:10.1186/s13068-014-0124-8)
Supplement: Additional file 7: — Co-occurrence profiles of the M1 protein families and GH6/GH48 across the learning set. Two heat maps display the combined co-occurrence profiles of the M1 protein families and two additional cellulases, GH6 and GH48, across the sets of the known phenotype-positive (Figure S1) and phenotype-negative (Figure S2) genomes, respectively. GH6 and GH48 were not assigned to module M1. The colors of the heat map cells represent the number of instances of each family in the respective genomes of the organisms (see legends and note that the counted number of instances was limited to a maximum of 10 per genome, as described in Methods). The phylogenetic relationships of the genomes are indicated by dendrograms alongside the rows of the heat maps. [file 13068_2014_124_MOESM7_ESM.pdf]

Figure S1

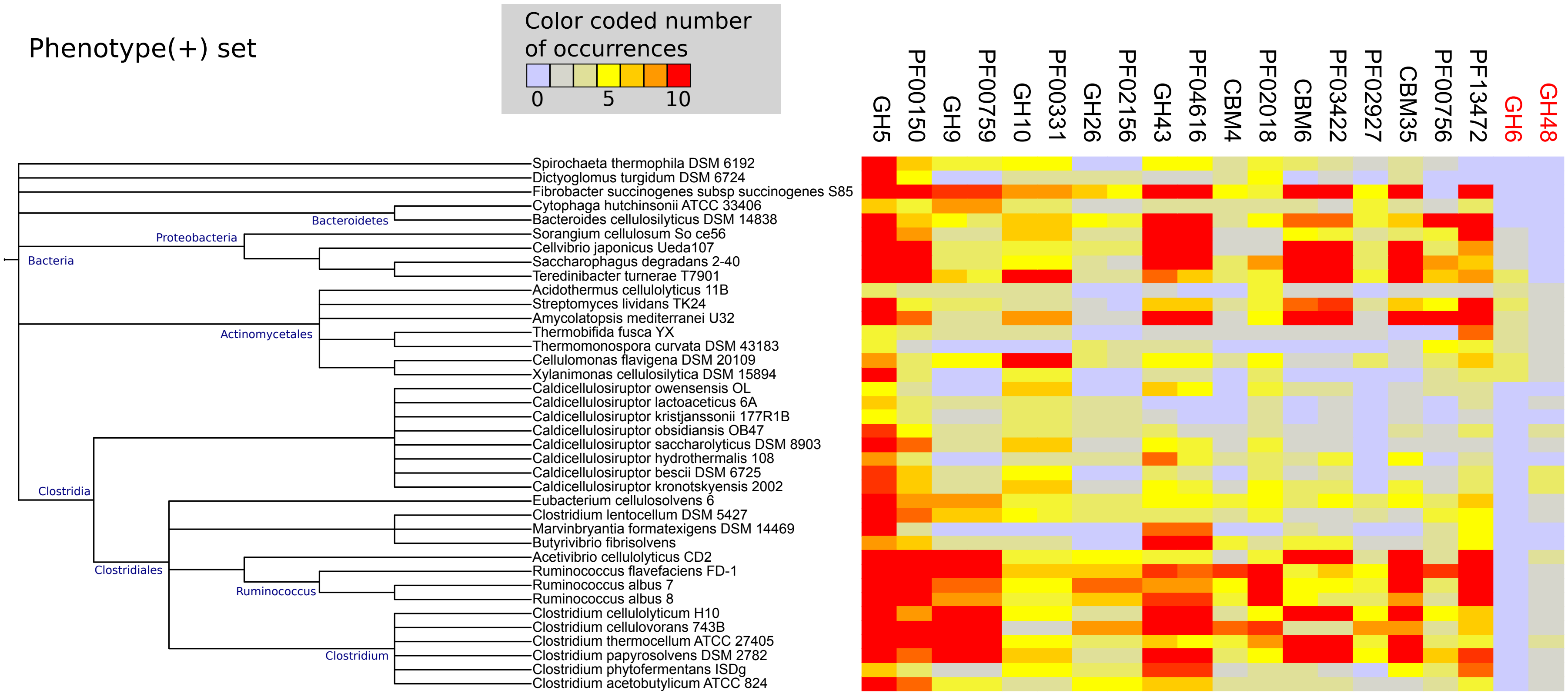

Phenotype(+) set
Color coded number of occurrences
0
5
10
GH5
PF00150
GH9
PF00759
GH10
PF00331
GH26
PF02156
GH43
PF04616
CBM4
PF02018
CBM6
PF03422
PF02927
CBM35
PF00756
PF13472
GH6
GH48
Bacteria
Bacteroidetes
Proteobacteria
Actinomycetales
Clostridia
Clostridiales
Ruminococcus
Clostridium
Spirochaeta thermophila DSM 6192
Dictyoglomus turgidum DSM 6724
Fibrobacter succinogenes subsp succinogenes S85
Cytophaga hutchinsonii ATCC 33406
Bacteroides cellulosilyticus DSM 14838
Sorangium cellulosum So ce56
Cellvibrio japonicus Ueda107
Saccharophagus degradans 2-40
Teredinibacter turnerae T7901
Acidothermus cellulolyticus 11B
Streptomyces lividans TK24
Amycolatopsis mediterranei U32
Thermobifida fusca YX
Thermomonospora curvata DSM 43183
Cellulomonas flavigena DSM 20109
Xylanimonas cellulosilytica DSM 15894
Caldicellulosiruptor owensensis OL
Caldicellulosiruptor lactoaceticus 6A
Caldicellulosiruptor kristjanssonii 177R1B
Caldicellulosiruptor obsidiansis OB47
Caldicellulosiruptor saccharolyticus DSM 8903
Caldicellulosiruptor hydrothermalis 108
Caldicellulosiruptor bescii DSM 6725
Caldicellulosiruptor kronotskyensis 2002
Eubacterium cellulosolvens 6
Clostridium lentocellum DSM 5427
Marvinbryantia formatexigens DSM 14469
Butyrivibrio fibrisolvens
Acetivibrio cellulolyticus CD2
Ruminococcus flavefaciens FD-1
Ruminococcus albus 7
Ruminococcus albus 8
Clostridium cellulolyticum H10
Clostridium cellulovorans 743B
Clostridium thermocellum ATCC 27405
Clostridium papyrosolvens DSM 2782
Clostridium phytofermentans ISDg
Clostridium acetobutylicum ATCC 824

Figure S2

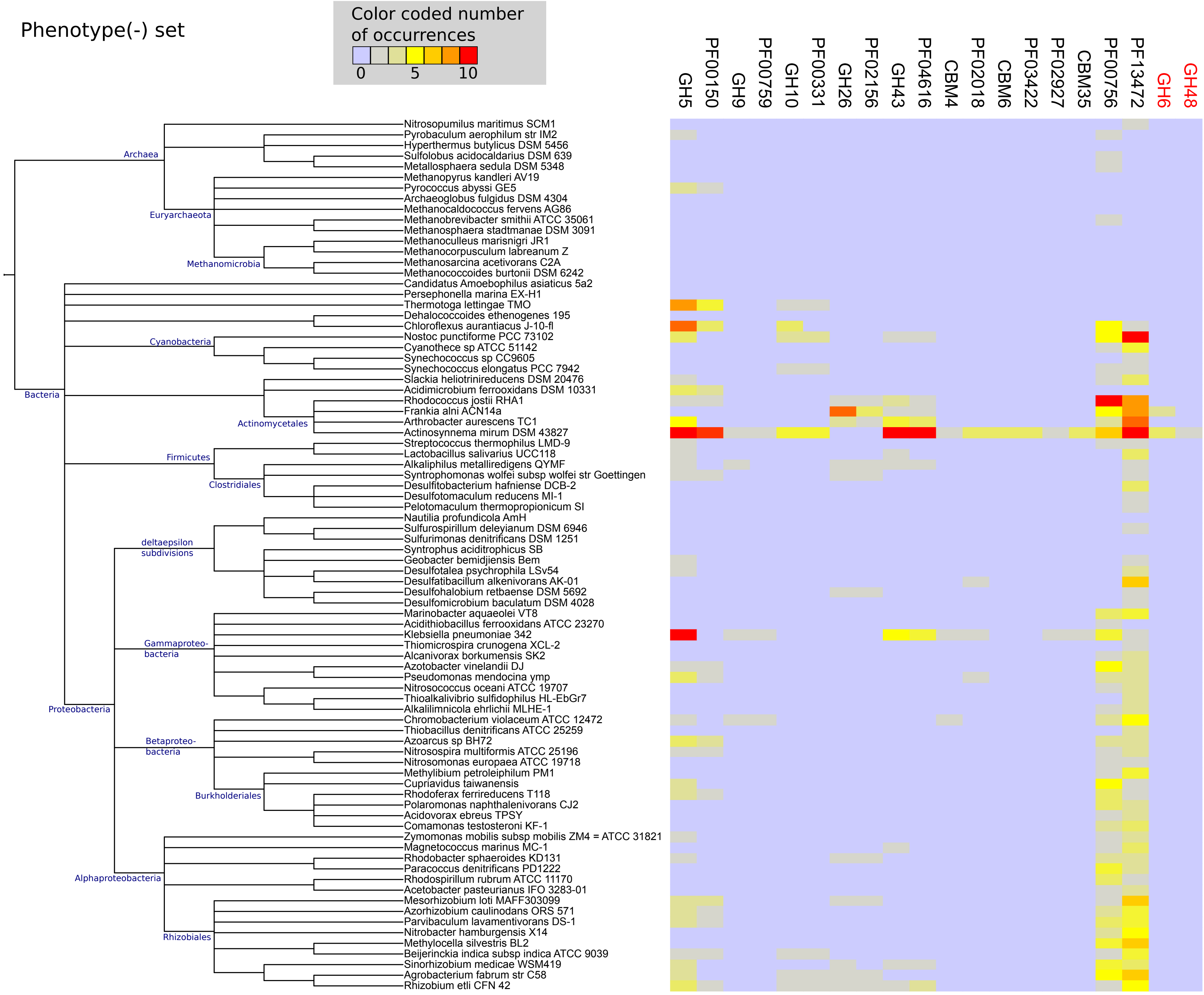
Phenotype(-) set
Color coded number of occurrences
0
5
10
GH5
PF00150
GH9
PF00759
GH10
PF00331
GH26
PF02156
GH43
PF04616
CBM4
PF02018
CBM6
PF03422
PF02927
CBM35
PF00756
PF13472
GH6
GH48
Archaea
Euryarchaeota
Methanomicrobia
Bacteria
Cyanobacteria
Actinomycetales
Firmicutes
Clostridiales
deltaepsilon subdivisions
Gammaproteo-bacteria
Proteobacteria
Betaproteo-bacteria
Burkholderiales
Alphaproteobacteria
Rhizobiales
Nitrosopumilus maritimus SCM1
Pyrobaculum aerophilum str IM2
Hyperthermus butylicus DSM 5456
Sulfolobus acidocaldarius DSM 639
Metallosphaera sedula DSM 5348
Methanopyrus kandleri AV19
Pyrococcus abyssi GE5
Archaeoglobus fulgidus DSM 4304
Methanocaldococcus fervens AG86
Methanobrevibacter smithii ATCC 35061
Methanosphaera stadtmanae DSM 3091
Methanoculleus marisnigri JR1
Methanocorpusculum labreanum Z
Methanosarcina acetivorans C2A
Methanococcoides burtonii DSM 6242
Candidatus Amoebophilus asiaticus 5a2
Persephonella marina EX-H1
Thermotoga lettingae TMO
Dehalococcoides ethenogenes 195
Chloroflexus aurantiacus J-10-fl
Nostoc punctiforme PCC 73102
Cyanothece sp ATCC 51142
Synechococcus sp CC9605
Synechococcus elongatus PCC 7942
Slackia heliotrinireducens DSM 20476
Acidimicrobium ferrooxidans DSM 10331
Rhodococcus jostii RHA1
Frankia alni ACN14a
Arthrobacter aurescens TC1
Actinosynnema mirum DSM 43827
Streptococcus thermophilus LMD-9
Lactobacillus salivarius UCC118
Alkaliphilus metalliredigens QYMF
Syntrophomonas wolfei subsp wolfei str Goettingen
Desulfitobacterium hafniense DCB-2
Desulfotomaculum reducens MI-1
Pelotomaculum thermopropionicum SI
Nautilia profundicola AmH
Sulfurospirillum deleyianum DSM 6946
Sulfurimonas denitrificans DSM 1251
Syntrophus aciditrophicus SB
Geobacter bemidjiensis Bem
Desulfotalea psychrophila LSv54
Desulfatibacillum alkenivorans AK-01
Desulfohalobium retbaense DSM 5692
Desulfomicrobium baculatum DSM 4028
Marinobacter aquaeolei VT8
Acidithiobacillus ferrooxidans ATCC 23270
Klebsiella pneumoniae 342
Thiomicrospira crunogena XCL-2
Alcanivorax borkumensis SK2
Azotobacter vinelandii DJ
Pseudomonas mendocina ymp
Nitrosococcus oceani ATCC 19707
Thioalkalivibrio sulfidophilus HL-EbGr7
Alkalilimnicola ehrlichii MLHE-1
Chromobacterium violaceum ATCC 12472
Thiobacillus denitrificans ATCC 25259
Azoarcus sp BH72
Nitrosospira multiformis ATCC 25196
Nitrosomonas europaea ATCC 19718
Methylibium petroleiphilum PM1
Cupriavidus taiwanensis
Rhodoferax ferrireducens T118
Polaromonas naphthalenivorans CJ2
Acidovorax ebreus TPSY
Comamonas testosteroni KF-1
Zymomonas mobilis subsp mobilis ZM4 = ATCC 31821
Magnetococcus marinus MC-1
Rhodobacter sphaeroides KD131
Paracoccus denitrificans PD1222
Rhodospirillum rubrum ATCC 11170
Acetobacter pasteurianus IFO 3283-01
Mesorhizobium loti MAFF303099
Azorhizobium caulinodans ORS 571
Parvibaculum lavamentivorans DS-1
Nitrobacter hamburgensis X14
Methylocella silvestris BL2
Beijerinckia indica subsp indica ATCC 9039
Sinorhizobium medicae WSM419
Agrobacterium fabrum str C58
Rhizobium etli CFN 42
