# Supplementary material for: Inference of phenotype-defining functional modules of protein families for microbial plant biomass degraders
Source: Biotechnol Biofuels. 2014 Sep 9;7:124. doi: 10.1186/s13068-014-0124-8 (PMC4189754; doi:10.1186/s13068-014-0124-8)
Supplement: Additional file 8: — Co-occurrence profiles of protein families that were weakly associated with M5 (across the learning set). Two heat maps displaying protein family co-occurrence profiles across the known phenotype-positive (Figure S1) and phenotype-negative (Figure S2) genomes. The columns of the heat maps represent the families that were weakly associated with the M5 module. These families did not satisfy the required threshold C = 0.01, but they belonged to the 50 protein families with the highest probabilities in the 16 topics that were used to create the M5 consensus module. Because the families failed to match the threshold, they were not counted for the consensus of M5. One example for such a family is GH48 (discussed in the main text). Families are ordered from left to right according to the number of topics in which they occurred. Families that occurred in less than 9 of the 16 topics are not displayed. We also added the cohesin and dockerin domains of M5 for a comparison. The colors of the heat map cells encode the number of instances of each family in the respective genomes of the organisms. [file 13068_2014_124_MOESM8_ESM.pdf]

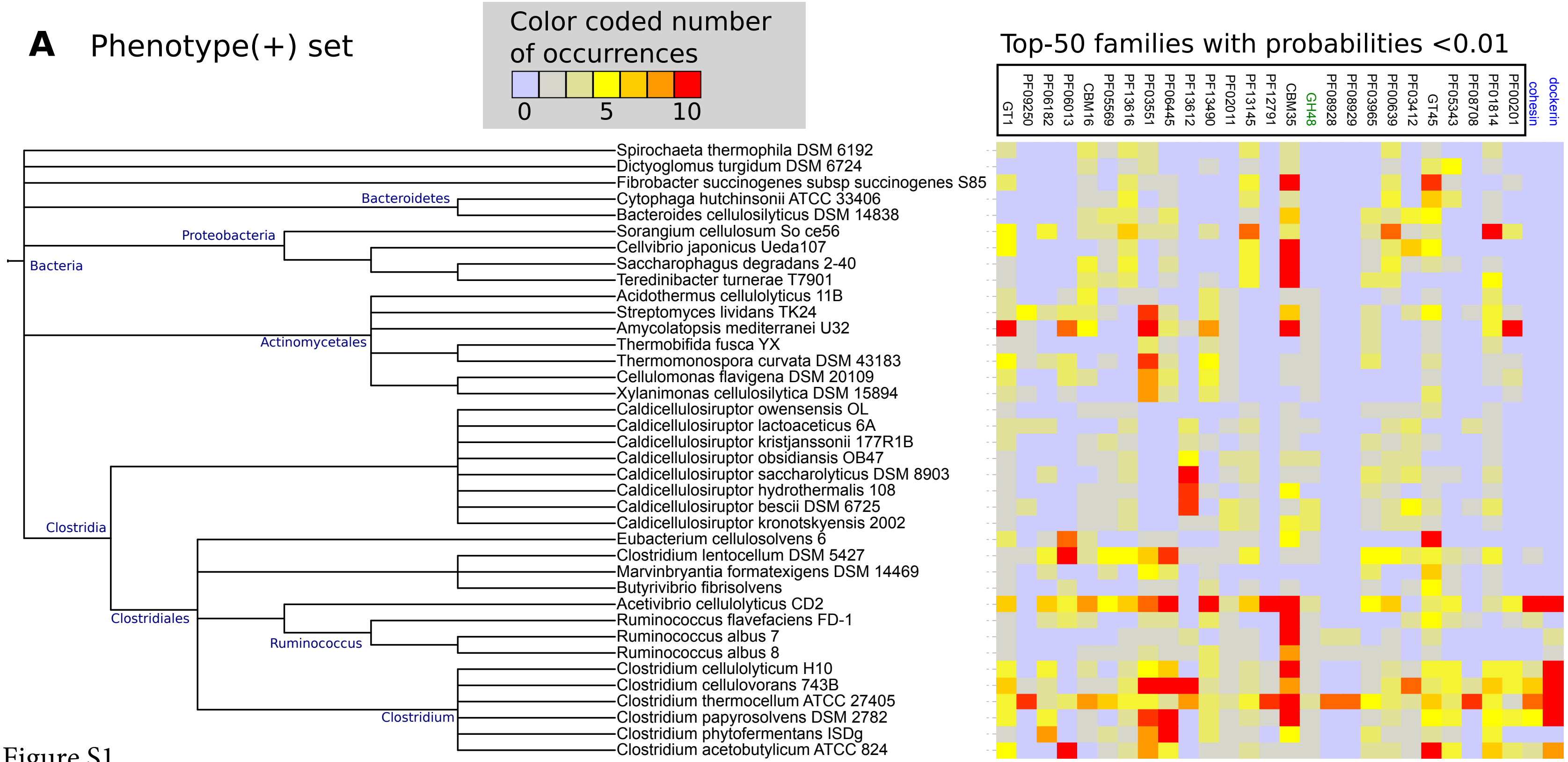
A
Phenotype(+) set
Color coded number of occurrences
0
5
10
Top-50 families with probabilities <0.01
GT1
PF09250
PF06182
PF06013
CBM16
PF05569
PF13616
PF03551
PF06445
PF13612
PF13490
PF02011
PF13145
PF12791
CBM35
GH48
PF08928
PF08929
PF03965
PF00639
PF03412
GT45
PF05343
PF08708
PF01814
PF00201
cohesin
dockerin
Bacteria
Bacteroidetes
Proteobacteria
Actinomycetales
Clostridia
Clostridiales
Ruminococcus
Clostridium
Spirochaeta thermophila DSM 6192
Dictyoglomus turgidum DSM 6724
Fibrobacter succinogenes subsp succinogenes S85
Cytophaga hutchinsonii ATCC 33406
Bacteroides cellulosilyticus DSM 14838
Sorangium cellulosum So ce56
Cellvibrio japonicus Ueda107
Saccharophagus degradans 2-40
Teredinibacter turnerae T7901
Acidothermus cellulolyticus 11B
Streptomyces lividans TK24
Amycolatopsis mediterranei U32
Thermobifida fusca YX
Thermomonospora curvata DSM 43183
Cellulomonas flavigena DSM 20109
Xylanimonas cellulosilytica DSM 15894
Caldicellulosiruptor owensensis OL
Caldicellulosiruptor lactoaceticus 6A
Caldicellulosiruptor kristjanssonii 177R1B
Caldicellulosiruptor obsidiansis OB47
Caldicellulosiruptor saccharolyticus DSM 8903
Caldicellulosiruptor hydrothermalis 108
Caldicellulosiruptor bescii DSM 6725
Caldicellulosiruptor kronotskyensis 2002
Eubacterium cellulosolvens 6
Clostridium lentocellum DSM 5427
Marvinbryantia formatexigens DSM 14469
Butyrivibrio fibrisolvens
Acetivibrio cellulolyticus CD2
Ruminococcus flavefaciens FD-1
Ruminococcus albus 7
Ruminococcus albus 8
Clostridium cellulolyticum H10
Clostridium cellulovorans 743B
Clostridium thermocellum ATCC 27405
Clostridium papyrosolvens DSM 2782
Clostridium phytofermentans ISDg
Clostridium acetobutylicum ATCC 824

Figure S1

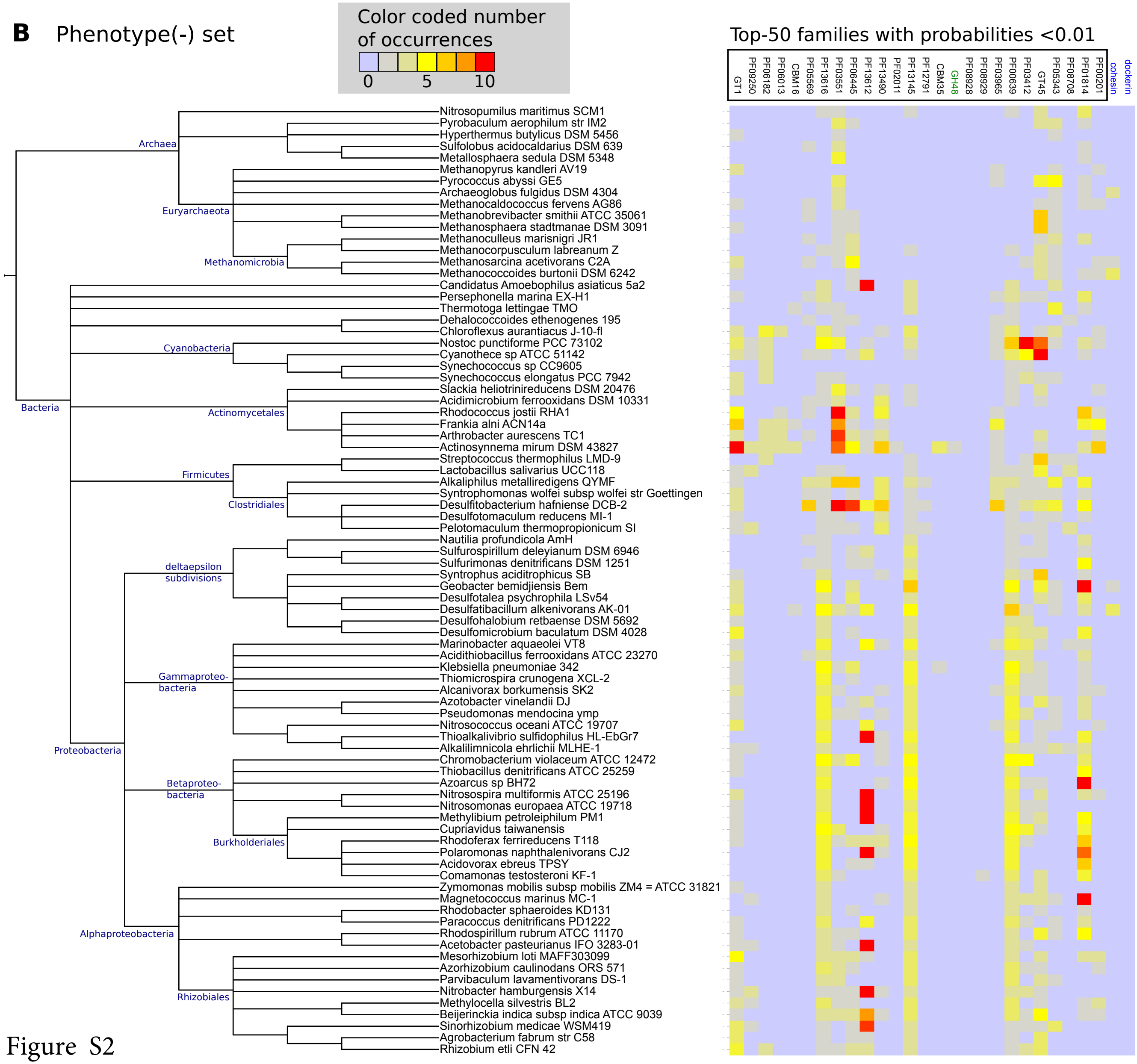
B
Phenotype(-) set
Color coded number of occurrences
0
5
10
Top-50 families with probabilities <0.01
GT1
PF09250
PF06182
PF06013
CBM16
PF05569
PF13616
PF03551
PF06445
PF13612
PF13490
PF02011
PF13145
PF12791
CBM35
GH48
PF08928
PF08929
PF03965
PF00639
PF03412
GT45
PF05343
PF08708
PF01814
PF00201
cohesin
dockerin
Archaea
Euryarchaeota
Methanomicrobia
Bacteria
Cyanobacteria
Actinomycetales
Firmicutes
Clostridiales
deltaepsilon subdivisions
Gammaproteo-bacteria
Betaproteo-bacteria
Burkholderiales
Proteobacteria
Alphaproteobacteria
Rhizobiales
Nitrosopumilus maritimus SCM1
Pyrobaculum aerophilum str IM2
Hyperthermus butylicus DSM 5456
Sulfolobus acidocaldarius DSM 639
Metallosphaera sedula DSM 5348
Methanopyrus kandleri AV19
Pyrococcus abyssi GE5
Archaeoglobus fulgidus DSM 4304
Methanocaldococcus fervens AG86
Methanobrevibacter smithii ATCC 35061
Methanosphaera stadtmanae DSM 3091
Methanoculleus marisnigri JR1
Methanocorpusculum labreanum Z
Methanosarcina acetivorans C2A
Methanococcoides burtonii DSM 6242
Candidatus Amoebophilus asiaticus 5a2
Persephonella marina EX-H1
Thermotoga lettingae TMO
Dehalococcoides ethenogenes 195
Chloroflexus aurantiacus J-10-fl
Nostoc punctiforme PCC 73102
Cyanothece sp ATCC 51142
Synechococcus sp CC9605
Synechococcus elongatus PCC 7942
Slackia heliotrinireducens DSM 20476
Acidimicrobium ferrooxidans DSM 10331
Rhodococcus jostii RHA1
Frankia alni ACN14a
Arthrobacter aurescens TC1
Actinosynnema mirum DSM 43827
Streptococcus thermophilus LMD-9
Lactobacillus salivarius UCC118
Alkaliphilus metalliredigens QYMF
Syntrophomonas wolfei subsp wolfei str Goettingen
Desulfitobacterium hafniense DCB-2
Desulfotomaculum reducens MI-1
Pelotomaculum thermopropionicum SI
Nautilia profundicola AmH
Sulfurospirillum deleyianum DSM 6946
Sulfurimonas denitrificans DSM 1251
Syntrophus aciditrophicus SB
Geobacter bemidjiensis Bem
Desulfotalea psychrophila LSv54
Desulfatibacillum alkenivorans AK-01
Desulfohalobium retbaense DSM 5692
Desulfomicrobium baculatum DSM 4028
Marinobacter aquaeolei VT8
Acidithiobacillus ferrooxidans ATCC 23270
Klebsiella pneumoniae 342
Thiomicrospira crunogena XCL-2
Alcanivorax borkumensis SK2
Azotobacter vinelandii DJ
Pseudomonas mendocina ymp
Nitrosococcus oceani ATCC 19707
Thioalkalivibrio sulfidophilus HL-EbGr7
Alkalilimnicola ehrlichii MLHE-1
Chromobacterium violaceum ATCC 12472
Thiobacillus denitrificans ATCC 25259
Azoarcus sp BH72
Nitrosospira multiformis ATCC 25196
Nitrosomonas europaea ATCC 19718
Methylibium petroleiphilum PM1
Cupriavidus taiwanensis
Rhodoferax ferrireducens T118
Polaromonas naphthalenivorans CJ2
Acidovorax ebreus TPSY
Comamonas testosteroni KF-1
Zymomonas mobilis subsp mobilis ZM4 = ATCC 31821
Magnetococcus marinus MC-1
Rhodobacter sphaeroides KD131
Paracoccus denitrificans PD1222
Rhodospirillum rubrum ATCC 11170
Acetobacter pasteurianus IFO 3283-01
Mesorhizobium loti MAFF303099
Azorhizobium caulinodans ORS 571
Parvibaculum lavamentivorans DS-1
Nitrobacter hamburgensis X14
Methylocella silvestris BL2
Beijerinckia indica subsp indica ATCC 9039
Sinorhizobium medicae WSM419
Agrobacterium fabrum str C58
Rhizobium etli CFN 42

Figure S2
