# Supplementary material for: Inference of phenotype-defining functional modules of protein families for microbial plant biomass degraders
Source: Biotechnol Biofuels. 2014 Sep 9;7:124. doi: 10.1186/s13068-014-0124-8 (PMC4189754; doi:10.1186/s13068-014-0124-8)
Supplement: Additional file 9: — Leave-one-out results for the consensus plant biomass degradation modules (PDMs) obtained with the threshold C = 0.005. The threshold C = 0.01 was used to convert the discrete topic probability distributions of the LDA model into potential functional modules (see Methods). In additional tests, we used the threshold C = 0.005 instead. This cutoff level is less strict and allowed families with smaller probabilities to be included in the potential functional modules. This also resulted in enlarged consensus modules for the PDMs. Table S1 summarizes the results obtained in leave-one-out validation for the PDMs M1 to M5 based on the threshold C = 0.005. [file 13068_2014_124_MOESM9_ESM.pdf]

Table S1 Leave-one-out results for the consensus plant biomass degradation modules (PDMs) obtained with the threshold *C* = 0.005.

		**M1**	**M2**	**M3**	**M4**	**M5**
Set of recurring modules (18 repetitions of analyses)	**Number of modules in set**	18	18	18	18	16
	**Average rank**	1.3 (±0.57)	2.4 (±0.61)	4.2 (±1.5)	6 (±1.6)	7.5 (±3.4)
Consensus PDM	**Size**	45	37	53	37	34
Performance evaluation	**LOO $F_{0.5}$-score (%)**	89.08	93.37	83.33	85.86	80
	**LOO recall (%)**	81.58	81.58	76.32	89.47	63.16
	**LOO precision (%)**	91.18	96.88	85.29	85	85.71
	**LOO accuracy (%)**	91.67	93.33	88.33	91.67	85
	**LOO MAC (%)**	88.96	90.18	85.11	91.08	79.14
	**Weight threshold (mean)**	57.77	45.96	56.47	56.77	41.27
