# Supplementary material for: Inference of phenotype-defining functional modules of protein families for microbial plant biomass degraders
Source: Biotechnol Biofuels. 2014 Sep 9;7:124. doi: 10.1186/s13068-014-0124-8 (PMC4189754; doi:10.1186/s13068-014-0124-8)
Supplement: Additional file 14: — Microbial isolate strains (lignocellulose degraders and non-degraders) that were used as the learning set. Table S1 represents a manually curated list of 120 phenotype-positive or phenotype-negative prokaryotic genomes, including the respective literature references. [file 13068_2014_124_MOESM14_ESM.pdf]

Table S1 – Microbial isolate strains (lignocellulose degraders and non-degraders) that were used as the learning set

We used and extended a manually curated set of lignocellulose and non-lignocellulose degrading microbial strains [1]. For our study, we identified 20 additional lignocellulose-degrading strains from the literature (see Methods), which are marked by bold numbers in the following table. The symbol * marks strains for which we provide another reference than the genome publication characterizing the metabolic capacities of the respective strain.

	**Microbial strain**		**Reference**
Lignocellulose degrading organisms	1	*Acidothermus cellulolyticus* 11B	Barabote et al 2009 [2]
	2	*Caldicellulosiruptor bescii* Z-1320, DSM 6725 *('Anaerocellum thermophilum')*	Yang et al 2009 [3]
	3	*Bryantella formatexigens* I-52, DSM 14469	Wolin et al 2003 [4]
	4	*Caldicellulosiruptor saccharolyticus* DSM 8903	Rainey et al 1994* [5]
	5	*Cellulomonas flavigena* 134, DSM 20109	Abt et al 2010 [6]
	6	*Cellvibrio japonicus Ueda* 107	DeBoy et al 2008 [7]
	7	*Clostridium cellulolyticum* H10	Petitdemange et al 1984* [8]
	8	*Clostridium phytofermentans* ISDg	Warnick et al 2002* [9]
	9	*Clostridium thermocellum* ATCC 27405	Feinberg et al 2011 [10]
	10	*Cytophaga hutchinsonii* ATCC 33406	Xie et al 2007 [11]
	11	*Dictyoglomus turgidum* DSM 6724	Brumm et al 2011 [12]
	12	*Fibrobacter succinogenes succinogenes* S85	Bae et al 1993* [13]
	13	*Ruminococcus flavefaciens* FD-1	Berg Miller et al 2009 [14]
	14	*Saccharophagus degradans* 2-40	Fraiberg et al 2010 [15]
	15	*Teredinibacter turnerae* T7901	Yang et al 2009 [16]
	16	*Thermobifida fusca* YX	Lykidis et al 2007 [17]
	17	*Thermomonospora curvata* DSM 43183	Chertkov et al 2011 [18]
	18	*Xylanimonas cellulosilytica* XIL07, DSM 15894	Foster et al 2010 [19]
	**19**	*Cellulosilyticum lentocellum* RHM5, DSM 5427	Miller et al 2011 [20]
	**20**	*Clostridium cellulovorans* 743B	Sleat et al 1984 * [21]
	**21**	*Caldicellulosiruptor lactoaceticus* 6A	Mladenovska et al * 1995 [22]
	**22**	*Spirochaeta thermophila* DSM 6192	Rainey et al * 1991 [23]
	**23**	*Acetivibrio cellulolyticus* CD2, DSM 1870	Saddler et al * 1981 [24]
	**24**	*Ruminococcus albus* 7	Suen et al 2011 [25]
	**25**	*Ruminococcus albus* 8	Devillard et al * 2004 [26]
	**26**	*Butyrivibrio fibrosolvens* 16/4	Hespell et al * 1987 [27]
	**27**	*Bacteroides cellulosilyticus* DSM 14838	Robert et al * 2007 [28]
	**28**	*Clostridium papyrosolvens* DSM 2782	Madden et al * 1982 [29]
	**29**	*Clostridium acetobutylicum* ATCC 824	Nölling et al 2001 [30]
	**30**	*Caldicellulosiruptor obsidiansis* OB47	Blumer-Schuette et al 2011 [31]

	**31**	*Caldicellulosiruptor hydrothermalis* 108	Blumer-Schuette et al 2011 [31]
	**32**	*Caldicellulosiruptor kristjanssonii* 177R1B, DSM 12137	Blumer-Schuette et al 2011 [31]
	**33**	*Caldicellulosiruptor kronotskyensis* 2002	Blumer-Schuette et al 2011 [31]
	**34**	*Caldicellulosiruptor owensensis* OL	Blumer-Schuette et al 2011 [31]
	**35**	*Streptomyces lividans* TK24	Kluepfel et al * 1986 [32]
	**36**	*Amycolatopsis mediterranei* U32	Zhao et al 2010 [33]
	**37**	*Sorangium cellulosum* So ce 56	Schneiker et al 2007 [34]
	**38**	*Eubacterium cellulosolvens* 6	Flint et al * 2008 [35]
Non-lignocellulose-degrading organisms	1	*Acetobacter pasteurianus* IFO 3283-01	Azuma et al 2009 [36]
	2	*Acidimicrobium ferrooxidans* DSM 10331	Clum et al 2009 [37]
	3	*Acidithiobacillus ferrooxidans* ATCC 23270	Valdés et al 2008 [38]
	4	*Actinosynnema mirum* DSM 43827	Land et al 2009 [39]
	5	*Agrobacterium tumefaciens* C58 (Cereon)	Wood et al 2001 [40]
	6	*Alcanivorax borkumensis* SK2	Schneiker et al 2006 [41]
	7	*Alkalilimnicola ehrlichei* MLHE-1	Hoeft et al 2007* [42]*
	8	*Alkaliphilus metalliredigens* QYMF	Fu et al 2009)* [43]
	9	*Archaeoglobus fulgidus* DSM 4304	Klenk et al 1997 [44]
	10	*Arthrobacter aurescens* TC1	Mongodin et al 2006 [45]
	11	*Azoarcus* sp. BH72	Krause et al 2006 [46]
	12	*Azorhizobium caulinodans* ORS 571	Liu et al 2011 [47]
	13	*Azotobacter vinelandii* DJ, ATCC BAA-1303	Setubal et al 2009 [48]
	14	*Beijerinckia indica indica* ATCC 9039	Tamas et al 2010 [49]
	15	*Candidatus amoebophilus asiaticus* 5a2	Schmitz-Esser et al 2010 [50]
	16	*Chloroflexus aurantiacus* J-10-fl	Tang et al 2011 [51]
	17	*Chromobacterium violaceum* ATCC 12472	Brazilian National Genome Project Consortium 2003 [52]
	18	*Comamonas testosteroni* KF-1	Ma et al 2009 [53]
	19	*Cupriavidus taiwanensis*	Amadou et al 2008 [54]
	20	*Cyanothece* sp. ATCC 51142	Welsh et al 2008 [55]
	21	*Dehalococcoides ethenogenes* 195	Seshadri et al 2005 [56]
	22	*Desulfatibacillum alkenivorans* AK-01	Callaghan et al 2012 [57]
	23	*Desulfitobacterium hafniense* DCB-2	Shinoda et al 2006) [58]
	24	*Desulfohalobium retbaense* DSM 5692	Spring et al 2010 [59]
	25	*Desulfomicrobium baculatum* DSM 4028	Copeland et al 2009 [60]
	26	*Desulfotalea psychrophila* LSv54	Rabus et al 2004 [61]
	27	*Desulfotomaculum reducens* MI-1	Junier et al 2010 [62]
	28	*Diaphorobacter* sp. TPSYc	Byrne-Bailey et al 2010 [63]
	29	*Frankia alni* ACN14a	Normand et al 2007 [64]
	30	*Geobacter bemidjiensis* Bem	Aklujkar et al 2010 [65]
	31	*Hyperthermus butylicus* DSM 5456	Brügger et al 2007 [66]
	32	*Klebsiella pneumoniae* 342	Yi et al 2010 [67]
	33	*Lactobacillus salivarius salivarius* UCC118	Jimenez et al 2010 [68]
	34	*Magnetococcus* sp. MC-1	Schübbe et al 2009 [69]
	35	*Marinobacter aquaeolei* VT8	Singer et al 2011* [70]
	36	*Mesorhizobium loti* MAFF303099	Kaneko et al 2000 [71]

	37	*Metallosphaera sedula* DSM 5348	Auernik et al 2008 [72]
	38	*Methanobrevibacter smithii* ATCC 35061	Hansen et al 2011 [73]
	39	*Methanocaldococcus fervens* AG86	Galperin and Cochrane 2009 [74]
	40	*Methanococcoides burtonii* DSM 6242	Saunders et al 2003 [75]
	41	*Methanocorpusculum labreanum* Z	Anderson et al 2009 [76]
	42	*Methanoculleus marisnigri* JR1	Anderson et al 2009 [76]
	43	*Methanopyrus kandleri* AV19	Slesarev et al 2002 [77]
	44	*Methanosarcina acetivorans* C2A	Galagan et al 2002 [78]
	45	*Methanosphaera stadtmanae* DSM 3091	Fricke et al 2006 [79]
	46	*Methylibium petroleiphilum* PM1	Kane et al 2007 [80]
	47	*Methylocella silvestris* BL2	Chen et al 2010 [81]
	48	*Nautilia profundicola* Am-H	Campbell et al 2009 [82]
	49	*Nitrobacter hamburgensis* X14	Starkenburg et al 2008 [83]
	50	*Nitrosococcus oceani* ATCC 19707	Klotz et al 2006 [84]
	51	*Nitrosomonas europaea* ATCC 19718	Chain et al 2003 [85]
	52	*Nitrosopumilus maritimus* SCM1	Walker et al 2010 [86]
	53	*Nitrosospira multiformis* ATCC 25196	Norton et al 2008 [87]
	54	*Nostoc punctiforme* PCC 73102	Meeks et al 2001 [88]
	55	*Paracoccus denitrificans* PD1222	Siddavattam et al 2011 [89]
	56	*Parvibaculum lavamentivorans* DS-1	Schleheck et al 2007 [90]
	57	*Pelotomaculum thermopropionicum* SI	Kosaka et al 2008 [91]
	58	*Persephonella marina* EX-H1	Reysenbach et al 2009 [92]
	59	*Polaromonas naphthalenivorans* CJ2	Yagi et al 2009 [93]
	60	*Pseudomonas mendocina* ymp	Guo et al 2011 [94]
	61	*Pyrobaculum aerophilum* IM2	Fitz-Gibbon et al 2002 [95]
	62	*Pyrococcus abyssi* GE5	Cohen et al 2003 [96]
	63	*Rhizobium etli* CFN 42	Fauvart et al 2011 [97]
	64	*Rhodobacter sphaeroides* KD131	Porter et al 2011 [98]
	65	*Rhodococcus* sp. RHA1	Takeda et al 2010 [99]
	66	*Rhodoferax ferrireducens* T118	Risso et al 2009 [100]
	67	*Rhodospirillum rubrum* ATCC 11170	Munk et al 2011 [101]
	68	*Sinorhizobium medicae* WSM419	Reeve et al 2010 [102]
	69	*Slackia heliotrinireducens* DSM 20476	Pukall et al 2009 [103]
	70	*Streptococcus thermophilus* LMD-9	Sun et al 2011 [104]
	71	*Sulfolobus acidocaldarius* DSM 639	Chen et al 2005 [105]
	72	*Sulfurospirillum deleyianum* DSM 6946	Sikorski et al 2010 [106]
	73	*Synechococcus elongatus* PCC 7942	Holtman et al 2005 [107]
	74	*Synechococcus* sp. CC9605	Jenkins et al 2006)* [108]
	75	*Syntrophomonas wolfei wolfei* Goettingen	Sieber et al 2010 [109]
	76	*Syntrophus aciditrophicus* SB	McInerney et al 2007 [110]
	77	*Thermotoga lettingae* TMO	Zhaxybayeva et al 2009 [111]
	78	*Thioalkalivibrio* sp. HL-EbGR7	Muyzer et al 2011 [112]
	79	*Thiobacillus denitrificans* ATCC 25259	Beller et al 2006 [113]
	80	*Thiomicrospira crunogena* XCL-2	Scott et al 2006 [114]
	81	*Thiomicrospira denitrificans* ATCC 33889	Sievert et al 2008 [115]
	82	*Zymomonas mobilis mobilis* ZM4	Pappas et al 2011 [116]

References

1. Weimann A, Trukhina Y, Pope PB, Konietzny SG, McHardy AC: **De novo prediction of the genomic components and capabilities for microbial plant biomass degradation from (meta-)genomes.** *Biotechnology for biofuels* 2013, **6:**24.
2. Barabote RD, Xie G, Leu DH, Normand P, Necsulea A, Adney WS, Xu XC, Lapidus A, Daubin V, Me C, et al: **Complete genome of the cellulolytic thermophile Acidothermus cellulolyticus 11B provides insights into its ecophysiological and evolutionary adaptations.** *Genome Research* 2009**:**1033-1043.
3. Yang SJ, Kataeva I, Hamilton-Brehm SD, Engle NL, Tschaplinski TJ, Doeppke C, Davis M, Westpheling J, Adams MWW: **Efficient Degradation of Lignocellulosic Plant Biomass, without Pretreatment, by the Thermophilic Anaerobe "Anaerocellum thermophilum" DSM 6725.** *Applied and environmental microbiology* 2009, **75:**4762-4769.
4. Wolin MJ, Miller TL, Collins MD, Lawson PA: **Formate-dependent growth and homoacetogenic fermentation by a bacterium from human feces: description of Bryantella formatexigens gen. nov., sp. nov.** *Applied and environmental microbiology* 2003, **69:**6321-6326.
5. Rainey FA, Donnison AM, Janssen PH, Saul D, Rodrigo A, Bergquist PL, Daniel RM, Stackebrandt E, Morgan HW: **Description of Caldicellulosiruptor saccharolyticus gen. nov., sp. nov: an obligately anaerobic, extremely thermophilic, cellulolytic bacterium.** *FEMS microbiology letters* 1994, **120:**263-266.
6. Abt B, Foster B, Lapidus A, Clum A, Sun H, Pukall Ru, Lucas S, Glavina Del Rio T, Nolan M, Tice H, et al: **Complete genome sequence of Cellulomonas flavigena type strain (134).** *Stand Genomic Sci* 2010, **3:**15-25.
7. DeBoy RT, Mongodin EF, Fouts DE, Tailford LE, Khouri H, Emerson JB, Mohamoud Y, Watkins K, Henrissat B, Gilbert HJ, Nelson KE: **Insights into plant cell wall degradation from the genome sequence of the soil bacterium Cellvibrio japonicus.** *Journal of bacteriology* 2008, **190:**5455-5463.
8. Petitdemange E, Biologique LDC, Ce V-l-n, Bacte C: **Clostridium cellulolyticum sp. nov. , a Cellulolytic, Mesophilic Species from Decayed Grass.** *International Journal* 1984**:**155-159.
9. Warnick TA, Methe BA, Leschine SB: **Clostridium phytofermentans sp. nov., a cellulolytic mesophile from forest soil.** *International journal of systematic and evolutionary microbiology* 2002, **52:**1155-1160.
10. Feinberg L, Foden J, Barrett T, Davenport KW, Bruce D, Detter C, Tapia R, Han C, Lapidus A, Lucas S, et al: **Complete genome sequence of the cellulolytic thermophile Clostridium thermocellum DSM1313.** *J Bacteriol* 2011, **193:**2906-2907.
11. Xie G, Bruce DC, Challacombe JF, Chertkov O, Detter JC, Gilna P, Han CS, Lucas S, Misra M, Myers GL, et al: **Genome sequence of the cellulolytic gliding bacterium Cytophaga hutchinsonii.** *Applied and environmental microbiology* 2007, **73:**3536-3546.
12. Brumm P, Hermanson S, Hochstein B, Boyum J, Hermersmann N, Gowda K, Mead D: **Mining Dictyoglomus turgidum for enzymatically active carbohydrases.** *Applied biochemistry and biotechnology* 2011, **163:**205-214.

13. Bae HD, McAllister Ta, Yanke J, Cheng KJ, Muir aD: **Effects of Condensed Tannins on Endoglucanase Activity and Filter Paper Digestion by Fibrobacter succinogenes S85.** *Applied and environmental microbiology* 1993, **59:**2132-2138.
14. Berg Miller ME, Antonopoulos Da, Rincon MT, Band M, Bari A, Akraiko T, Hernandez A, Thimmapuram J, Henrissat B, Coutinho PM, et al: **Diversity and strain specificity of plant cell wall degrading enzymes revealed by the draft genome of Ruminococcus flavefaciens FD-1.** *PLoS One* 2009, **4:**e6650.
15. Fraiberg M, Borovok I, Weiner RM, Lamed R: **Discovery and characterization of cadherin domains in Saccharophagus degradans 2-40.** *J Bacteriol* 2010, **192:**1066-1074.
16. Yang JC, Madupu R, Durkin aS, Ekborg Na, Pedamallu CS, Hostetler JB, Radune D, Toms BS, Henrissat B, Coutinho PM, et al: **The complete genome of Teredinibacter turnerae T7901: an intracellular endosymbiont of marine wood-boring bivalves (shipworms).** *PLoS One* 2009, **4:**e6085.
17. Lykidis A, Mavromatis K, Ivanova N, Anderson I, Land M, DiBartolo G, Martinez M, Lapidus A, Lucas S, Copeland A, et al: **Genome sequence and analysis of the soil cellulolytic actinomycete Thermobifida fusca YX.** *J Bacteriol* 2007, **189:**2477-2486.
18. Chertkov O, Sikorski J, Nolan M, Lapidus A, Lucas S, Del Rio TG, Tice H, Cheng J-F, Goodwin L, Pitluck S, et al: **Complete genome sequence of Thermomonospora curvata type strain (B9).** *Stand Genomic Sci* 2011, **4:**13-22.
19. Foster B, Pukall Ru, Abt B, Nolan M, Glavina Del Rio T, Chen F, Lucas S, Tice H, Pitluck S, Cheng J-F, et al: **Complete genome sequence of Xylanimonas cellulosilytica type strain (XIL07).** *Stand Genomic Sci* 2010, **2:**1-8.
20. Miller DA, Suen G, Bruce D, Copeland A, Cheng JF, Detter C, Goodwin LA, Han CS, Hauser LJ, Land ML, et al: **Complete genome sequence of the cellulose-degrading bacterium Cellulosilyticum lentocellum.** *J Bacteriol* 2011, **193:**2357-2358.
21. Sleat R, Mah RA, Robinson R: **Isolation and Characterization of an Anaerobic, Cellulolytic Bacterium, Clostridium cellulovorans sp. nov.** *Applied and environmental microbiology* 1984, **48:**88-93.
22. Mladenovska Z, Mathrani IM, Ahring BK: **Isolation and Characterization of Caldicellulosiruptor Lactoaceticus Sp-Nov, an Extremely Thermophilic, Cellulolytic, Anaerobic Bacterium.** *Arch Microbiol* 1995, **163:**223-230.
23. Rainey FA, Janssen PH, Wild DJC, Morgan HW: **Isolation and Characterization of an Obligately Anaerobic, Polysaccharolytic, Extremely Thermophilic Member of the Genus Spirochaeta.** *Arch Microbiol* 1991, **155:**396-401.
24. Saddler JN, Khan AW: **Cellulolytic enzyme system of Acetivibrio cellulolyticus.** *Canadian journal of microbiology* 1981, **27:**288-294.
25. Suen G, Stevenson DM, Bruce DC, Chertkov O, Copeland A, Cheng JF, Detter C, Detter JC, Goodwin LA, Han CS, et al: **Complete genome of the cellulolytic ruminal bacterium Ruminococcus albus 7.** *J Bacteriol* 2011, **193:**5574-5575.
26. Devillard E, Goodheart DB, Karnati SK, Bayer EA, Lamed R, Miron J, Nelson KE, Morrison M: **Ruminococcus albus 8 mutants defective in cellulose degradation are deficient in two processive endocellulases, Cel48A and Cel9B, both of which possess a novel modular architecture.** *J Bacteriol* 2004, **186:**136-145.
27. Hespell RB, Wolf R, Bothast RJ: **Fermentation of xylans by Butyrivibrio fibrisolvens and other ruminal bacteria.** *Applied and environmental microbiology* 1987, **53:**2849-

2853.
28. Robert C, Chassard C, Lawson PA, Bernalier-Donadille A: **Bacteroides cellulosilyticus sp. nov., a cellulolytic bacterium from the human gut microbial community.** *International journal of systematic and evolutionary microbiology* 2007, **57:**1516-1520.
29. Madden RH, Bryder MJ, Poole NJ: **Isolation and Characterization of an Anaerobic, Cellulolytic Bacterium, Clostridium-Papyrosolvens Sp-Nov.** *Int J Syst Bacteriol* 1982, **32:**87-91.
30. Nolling J, Breton G, Omelchenko MV, Makarova KS, Zeng Q, Gibson R, Lee HM, Dubois J, Qiu D, Hitti J, et al: **Genome sequence and comparative analysis of the solvent-producing bacterium Clostridium acetobutylicum.** *J Bacteriol* 2001, **183:**4823-4838.
31. Blumer-Schuette SE, Ozdemir I, Mistry D, Lucas S, Lapidus A, Cheng JF, Goodwin LA, Pitluck S, Land ML, Hauser LJ, et al: **Complete genome sequences for the anaerobic, extremely thermophilic plant biomass-degrading bacteria Caldicellulosiruptor hydrothermalis, Caldicellulosiruptor kristjanssonii, Caldicellulosiruptor kronotskyensis, Caldicellulosiruptor owensensis, and Caldicellulosiruptor lactoaceticus.** *J Bacteriol* 2011, **193:**1483-1484.
32. Kluepfel D, Shareck F, Mondou F, Morosoli R: **Characterization of Cellulase and Xylanase Activities of Streptomyces-Lividans.** *Appl Microbiol Biot* 1986, **24:**230-234.
33. Zhao W, Zhong Y, Yuan H, Wang J, Zheng H, Wang Y, Cen X, Xu F, Bai J, Han X, et al: **Complete genome sequence of the rifamycin SV-producing Amycolatopsis mediterranei U32 revealed its genetic characteristics in phylogeny and metabolism.** *Cell research* 2010, **20:**1096-1108.
34. Schneiker S, Perlova O, Kaiser O, Gerth K, Alici A, Altmeyer MO, Bartels D, Bekel T, Beyer S, Bode E, et al: **Complete genome sequence of the myxobacterium Sorangium cellulosum.** *Nature Biotechnology* 2007, **25:**1281-1289.
35. Flint HJ, Bayer EA, Rincon MT, Lamed R, White BA: **Polysaccharide utilization by gut bacteria: Potential for new insights from genomic analysis.** *Nature Reviews Microbiology* 2008, **6:**121-131.
36. Azuma Y, Hosoyama A, Matsutani M, Furuya N, Horikawa H, Harada T, Hirakawa H, Kuhara S, Matsushita K, Fujita N, Shirai M: **Whole-genome analyses reveal genetic instability of Acetobacter pasteurianus.** *Nucleic acids research* 2009, **37:**5768-5783.
37. Clum A, Nolan M, Lang E, Glavina Del Rio T, Tice H, Copeland A, Cheng J-F, Lucas S, Chen F, Bruce D, et al: **Complete genome sequence of Acidimicrobium ferrooxidans type strain (ICP).** *Stand Genomic Sci* 2009, **1:**38-45.
38. Valdés J, Pedroso I, Quatrini R, Dodson RJ, Tettelin H, Blake R, Eisen Ja, Holmes DS: **Acidithiobacillus ferrooxidans metabolism: from genome sequence to industrial applications.** *BMC genomics* 2008, **9:**597.
39. Land M, Lapidus A, Mayilraj S, Chen F, Copeland A, Del Rio TG, Nolan M, Lucas S, Tice H, Cheng J-F, et al: **Complete genome sequence of Actinosynnema mirum type strain (101).** *Stand Genomic Sci* 2009, **1:**46-53.
40. Wood DW, Setubal JC, Kaul R, Monks DE, Kitajima JP, Okura VK, Zhou Y, Chen L, Wood GE, Almeida NF, et al: **The genome of the natural genetic engineer Agrobacterium tumefaciens C58.** *Science (New York, NY)* 2001, **294:**2317-2323.
41. Schneiker S, Martins dos Santos ViaP, Bartels D, Bekel T, Brecht M, Buhrmester J, Chernikova TN, Denaro R, Ferrer M, Gertler C, et al: **Genome sequence of the**

ubiquitous hydrocarbon-degrading marine bacterium Alcanivorax borkumensis. *Nature biotechnology* 2006, **24:**997-1004.

42. Hoeft SE, Blum JS, Stolz JF, Tabita FR, Witte B, King GM, Santini JM, Oremland RS: **Alkalilimnicola ehrlichii sp. nov., a novel, arsenite-oxidizing haloalkaliphilic gammaproteobacterium capable of chemoautotrophic or heterotrophic growth with nitrate or oxygen as the electron acceptor.** *International journal of systematic and evolutionary microbiology* 2007, **57:**504-512.
43. Fu H-l, Meng Y, Ordóñez E, Villadangos AF, Bhattacharjee H, Gil JA, Mateos LM, Rosen BP: **Properties of arsenite efflux permeases (Acr3) from Alkaliphilus metalliredigens and Corynebacterium glutamicum.** *The Journal of biological chemistry* 2009, **284:**19887-19895.
44. Klenk HP, Clayton Ra, Tomb JF, White O, Nelson KE, Ketchum Ka, Dodson RJ, Gwinn M, Hickey EK, Peterson JD, et al: **The complete genome sequence of the hyperthermophilic, sulphate-reducing archaeon Archaeoglobus fulgidus.** *Nature* 1997, **390:**364-370.
45. Mongodin EF, Shapir N, Daugherty SC, DeBoy RT, Emerson JB, Shvartzbeyn A, Radune D, Vamathevan J, Riggs F, Grinberg V, et al: **Secrets of soil survival revealed by the genome sequence of Arthrobacter aurescens TC1.** *PLoS genetics* 2006, **2:**e214.
46. Krause A, Ramakumar A, Bartels D, Battistoni F, Bekel T, Boch J, B\"ohm M, Friedrich F, Hurek T, Krause L, et al: **Complete genome of the mutualistic, N2-fixing grass endophyte Azoarcus sp. strain BH72.** *Nature biotechnology* 2006, **24:**1385-1391.
47. Liu C-T, Lee K-B, Wang Y-S, Peng M-H, Lee K-T, Suzuki S, Suzuki T, Oyaizu H: **Involvement of the azorhizobial chromosome partition gene (parA) in the onset of bacteroid differentiation during Sesbania rostrata stem nodule development.** *Applied and environmental microbiology* 2011, **77:**4371-4382.
48. Setubal JC, dos Santos P, Goldman BS, Ertesvag H, Espin G, Rubio LM, Valla S, Almeida NF, Balasubramanian D, Cromes L, et al: **Genome sequence of Azotobacter vinelandii, an obligate aerobe specialized to support diverse anaerobic metabolic processes.** *J Bacteriol* 2009, **191:**4534-4545.
49. Tamas I, Dedysh SN, Liesack W, Stott MB, Alam M, Murrell JC, Dunfield PF: **Complete genome sequence of Beijerinckia indica subsp. indica.** *J Bacteriol* 2010, **192:**4532-4533.
50. Schmitz-Esser S, Tischler P, Arnold R, Montanaro J, Wagner M, Rattei T, Horn M: **The genome of the amoeba symbiont "Candidatus Amoebophilus asiaticus" reveals common mechanisms for host cell interaction among amoeba-associated bacteria.** *J Bacteriol* 2010, **192:**1045-1057.
51. Tang K-h, Barry K, Chertkov O, Dalin E, Han CS, Hauser LJ, Honchak BM, Karbach LE, Land ML, Lapidus A, et al: **Complete genome sequence of the filamentous anoxygenic phototrophic bacterium Chloroflexus aurantiacus.** *BMC genomics* 2011, **12:**334.
52. Brazilian National Genome Project Consortium: **The complete genome sequence of Chromobacterium violaceum reveals remarkable and exploitable bacterial adaptability.** *Proc Natl Acad Sci U S A* 2003, **100:**11660-11665.
53. Ma Y-F, Zhang Y, Zhang J-y, Chen D-w, Zhu Y, Zheng H, Wang S-y, Jiang C-y, Zhao G-p, Liu S-j: **The complete genome of Comamonas testosteroni reveals its genetic adaptations to changing environments.** *Applied and environmental microbiology* 2009,

75:6812-6819.

54. Amadou C, Mangenot S, Glew M, Bontemps C, Capela D, Dossat C, Marchetti M, Servin B, Saad M, Schenowitz C, et al: **Genome sequence of the -rhizobium Cupriavidus taiwanensis and comparative genomics of rhizobia.** *Genome Research* 2008**:**1472-1483.
55. Welsh Ea, Liberton M, Stöckel J, Loh T, Elvitigala T, Wang C, Wollam A, Fulton RS, Clifton SW, Jacobs JM, et al: **The genome of Cyanothece 51142, a unicellular diazotrophic cyanobacterium important in the marine nitrogen cycle.** *Proc Natl Acad Sci U S A* 2008, **105:**15094-15099.
56. Seshadri R, Adrian L, Fouts DE, Eisen Ja, Phillippy AM, Methe Ba, Ward NL, Nelson WC, Deboy RT, Khouri HM, et al: **Genome sequence of the PCE-dechlorinating bacterium Dehalococcoides ethenogenes.** *Science (New York, NY)* 2005, **307:**105-108.
57. Callaghan AV, Morris BEL, Pereira IAC, McInerney MJ, Austin RN, Groves JT, Kukor JJ, Suflita JM, Young LY, Zylstra GJ, Wawrik B: **The genome sequence of Desulfatibacillum alkenivorans AK-01: a blueprint for anaerobic alkane oxidation.** *Environmental Microbiology* 2012, **14:**101-113.
58. Shinoda Y, Ikenaga Y, Abe M, Naito K, Inatomi K, Furukawa K, Inui M, Yukawa H: **Complete Genome Sequence of the Dehalorespiring Bacterium Desulfitobacterium hafniense Y51 and Comparison with Dehalococcoides ethenogenes 195.** *J Bacteriol* 2006, **188:**2262-2274.
59. Spring S, Nolan M, Lapidus A, Glavina Del Rio T, Copeland A, Tice H, Cheng J-F, Lucas S, Land M, Chen F, et al: **Complete genome sequence of Desulfohalobium retbaense type strain (HR(100)).** *Stand Genomic Sci* 2010, **2:**38-48.
60. Copeland A, Spring S, Göker M, Schneider S, Lapidus A, Del Rio TG, Tice H, Cheng J-F, Chen F, Nolan M, et al: **Complete genome sequence of Desulfomicrobium baculatum type strain (X).** *Stand Genomic Sci* 2009, **1:**29-37.
61. Rabus R, Ruepp A, Frickey T, Rattei T, Fartmann B, Stark M, Bauer M, Zibat A, Lombardot T, Becker I, et al: **The genome of Desulfotalea psychrophila, a sulfate-reducing bacterium from permanently cold Arctic sediments.** *Environ Microbiol* 2004, **6:**887-902.
62. Junier P, Junier T, Podell S, Sims DR, Detter JC, Lykidis A, Han CS, Wigginton NS, Gaasterland T, Bernier-Latmani R: **The genome of the Gram-positive metal- and sulfate-reducing bacterium Desulfotomaculum reducens strain MI-1.** *Environmental Microbiology* 2010, **12:**2738-2754.
63. Byrne-Bailey KG, Weber Ka, Chair AH, Bose S, Knox T, Spanbauer TL, Chertkov O, Coates JD: **Completed genome sequence of the anaerobic iron-oxidizing bacterium Acidovorax ebreus strain TPSY.** *J Bacteriol* 2010, **192:**1475-1476.
64. Normand P, Lapierre P, Tisa LS, Gogarten JP, Alloisio N, Bagnarol E, Bassi CA, Berry AM, Bickhart DM, Choisne N, et al: **Genome characteristics of facultatively symbiotic Frankia sp. strains reflect host range and host plant biogeography.** *Genome Research* 2007**:**7-15.
65. Aklujkar M, Young ND, Holmes D, Chavan M, Risso C, Kiss HE, Han CS, Land ML, Lovley DR: **The genome of Geobacter bemidjiensis, exemplar for the subsurface clade of Geobacter species that predominate in Fe(III)-reducing subsurface environments.** *BMC genomics* 2010, **11:**490.
66. Brügger K, Chen L, Stark M, Zibat A, Redder P, Ruepp A, Awayez M, She Q, Garrett Ra,

Klenk H-P: **The genome of Hyperthermus butylicus: a sulfur-reducing, peptide fermenting, neutrophilic Crenarchaeote growing up to 108 degrees C.** *Archaea (Vancouver, BC)* 2007, **2:**127-135.

67. Yi H, Xi Y, Liu J, Wang J, Wu J, Xu T, Chen W, Chen B, Lin M, Wang H, et al: **Sequence analysis of pKF3-70 in Klebsiella pneumoniae: probable origin from R100-like plasmid of Escherichia coli.** *PLoS One* 2010, **5:**e8601.
68. Jimenez E, Langa S, Martin V, Arroyo R, Martin R, Fernandez L, Rodriguez JM: **Complete genome sequence of Lactobacillus fermentum CECT 5716, a probiotic strain isolated from human milk.** *J Bacteriol* 2010, **192:**4800.
69. Schübbe S, Williams TJ, Xie G, Kiss HE, Brettin TS, Martinez D, Ross Ca, Sch\"uler D, Cox BL, Nealson KH, Bazylinski Da: **Complete genome sequence of the chemolithoautotrophic marine magnetotactic coccus strain MC-1.** *Applied and environmental microbiology* 2009, **75:**4835-4852.
70. Singer E, Webb EA, Nelson WC, Heidelberg JF, Ivanova N, Pati A, Edwards KJ: **Genomic potential of Marinobacter aquaeolei, a biogeochemical "opportunitroph".** *Applied and environmental microbiology* 2011, **77:**2763-2771.
71. Kaneko T, Nakamura Y, Sato S, Asamizu E, Kato T, Sasamoto S, Watanabe a, Idesawa K, Ishikawa a, Kawashima K, et al: **Complete genome structure of the nitrogen-fixing symbiotic bacterium Mesorhizobium loti (supplement).** *DNA research : an international journal for rapid publication of reports on genes and genomes* 2000, **7:**381-406.
72. Auernik KS, Maezato Y, Blum PH, Kelly RM: **The genome sequence of the metal-mobilizing, extremely thermoacidophilic archaeon Metallosphaera sedula provides insights into bioleaching-associated metabolism.** *Applied and environmental microbiology* 2008, **74:**682-692.
73. Hansen EE, Lozupone Ca, Rey FE, Wu M, Guruge JL, Narra A, Goodfellow J, Zaneveld JR, McDonald DT, Goodrich Ja, et al: **Pan-genome of the dominant human gut-associated archaeon, Methanobrevibacter smithii, studied in twins.** *Proc Natl Acad Sci U S A* 2011, **108 Suppl** 4599-4606.
74. Galperin MY, Cochrane GR: **Nucleic Acids Research annual Database Issue and the NAR online Molecular Biology Database Collection in 2009.** *Nucleic acids research* 2009, **37:**D1-4.
75. Saunders NFW, Thomas T, Curmi PMG, Mattick JS, Kuczek E, Slade R, Davis J, Franzmann PD, Boone D, Rusterholtz K, et al: **Mechanisms of thermal adaptation revealed from the genomes of the Antarctic Archaea Methanogenium frigidum and Methanococcoides burtonii.** *Genome Research* 2003, **13:**1580-1588.
76. Anderson I, Ulrich LE, Lupa B, Susanti D, Porat I, Hooper SD, Lykidis A, Sieprawska-Lupa M, Dharmarajan L, Goltsman E, et al: **Genomic characterization of methanomicrobiales reveals three classes of methanogens.** *PLoS One* 2009, **4:**e5797.
77. Slesarev AI, Mezhevaya KV, Makarova KS, Polushin NN, Shcherbinina OV, Shakhova VV, Belova GI, Aravind L, Natale Da, Rogozin IB, et al: **The complete genome of hyperthermophile Methanopyrus kandleri AV19 and monophyly of archaeal methanogens.** *Proc Natl Acad Sci U S A* 2002, **99:**4644-4649.
78. Galagan JE, Nusbaum C, Roy A, Endrizzi MG, Macdonald P, FitzHugh W, Calvo S, Engels R, Smirnov S, Atnoor D, et al: **The genome of M. acetivorans reveals extensive metabolic and physiological diversity.** *Genome Research* 2002, **12:**532-542.

79. Fricke WF, Seedorf H, Henne A, Kruer M, Liesegang H, Hedderich R, Gottschalk G, Thauer RK: **The genome sequence of Methanosphaera stadtmanae reveals why this human intestinal archaeon is restricted to methanol and H2 for methane formation and ATP synthesis.** *J Bacteriol* 2006, **188:**642-658.
80. Kane SR, Chakicherla AY, Chain PSG, Schmidt R, Shin MW, Legler TC, Scow KM, Larimer FW, Lucas SM, Richardson PM, Hristova KR: **Whole-genome analysis of the methyl tert-butyl ether-degrading beta-proteobacterium Methylibium petroleiphilum PM1.** *J Bacteriol* 2007, **189:**1931-1945.
81. Chen Y, Crombie A, Rahman MT, Dedysh SN, Liesack W, Stott MB, Alam M, Theisen AR, Murrell JC, Dunfield PF: **Complete genome sequence of the aerobic facultative methanotroph Methylocella silvestris BL2.** *J Bacteriol* 2010, **192:**3840-3841.
82. Campbell BJ, Smith JL, Hanson TE, Klotz MG, Stein LY, Lee CK, Wu D, Robinson JM, Khouri HM, Eisen JA, Cary SC: **Adaptations to submarine hydrothermal environments exemplified by the genome of Nautilia profundicola.** *PLoS genetics* 2009, **5:**e1000362.
83. Starkenburg SR, Larimer FW, Stein LY, Klotz MG, Chain PSG, Sayavedra-Soto La, Poret-Peterson AT, Gentry ME, Arp DJ, Ward B, Bottomley PJ: **Complete genome sequence of Nitrobacter hamburgensis X14 and comparative genomic analysis of species within the genus Nitrobacter.** *Applied and environmental microbiology* 2008, **74:**2852-2863.
84. Klotz MG, Arp DJ, Chain PS, El-Sheikh AF, Hauser LJ, Hommes NG, Larimer FW, Malfatti SA, Norton JM, Poret-Peterson AT, et al: **Complete genome sequence of the marine, chemolithoautotrophic, ammonia-oxidizing bacterium Nitrosococcus oceani ATCC 19707.** *Applied and environmental microbiology* 2006, **72:**6299-6315.
85. Chain P, Lamerdin J, Larimer F, Regala W, Lao V, Land M, Hauser L, Hooper A, Klotz M, Norton J, et al: **Complete genome sequence of the ammonia-oxidizing bacterium and obligate chemolithoautotroph Nitrosomonas europaea.** *J Bacteriol* 2003, **185:**2759-2773.
86. Walker CB, de la Torre JR, Klotz MG, Urakawa H, Pinel N, Arp DJ, Brochier-Armanet C, Chain PSG, Chan PP, Gollabgir a, et al: **Nitrosopumilus maritimus genome reveals unique mechanisms for nitrification and autotrophy in globally distributed marine crenarchaea.** *Proc Natl Acad Sci U S A* 2010, **107:**8818-8823.
87. Norton JM, Klotz MG, Stein LY, Arp DJ, Bottomley PJ, Chain PSG, Hauser LJ, Land ML, Larimer FW, Shin MW, Starkenburg SR: **Complete genome sequence of Nitrosospira multiformis, an ammonia-oxidizing bacterium from the soil environment.** *Applied and environmental microbiology* 2008, **74:**3559-3572.
88. Meeks JC, Elhai J, Thiel T, Potts M, Larimer F, Lamerdin J, Predki P, Atlas R: **An overview of the genome of Nostoc punctiforme, a multicellular, symbiotic cyanobacterium.** *Photosynthesis Research* 2001**:**85-106.
89. Siddavattam D, Karegoudar TB, Mudde SK, Kumar N, Baddam R, Avasthi TS, Ahmed N: **Genome of a novel isolate of Paracoccus denitrificans capable of degrading N,N-dimethylformamide.** *J Bacteriol* 2011, **193:**5598-5599.
90. Schleheck D, Knepper TP, Eichhorn P, Cook AM: **Parvibaculum lavamentivorans DS-1T degrades centrally substituted congeners of commercial linear alkylbenzenesulfonate to sulfophenyl carboxylates and sulfophenyl dicarboxylates.** *Applied and environmental microbiology* 2007, **73:**4725-4732.
91. Kosaka T, Kato S, Shimoyama T, Ishii S, Abe T, Watanabe K: **The genome of**

Pelotomaculum thermopropionicum reveals niche-associated evolution in anaerobic microbiota. *Genome Res* 2008, **18:**442-448.
92. Reysenbach A-L, Hamamura N, Podar M, Griffiths E, Ferreira S, Hochstein R, Heidelberg J, Johnson J, Mead D, Pohorille a, et al: **Complete and draft genome sequences of six members of the Aquificales.** *J Bacteriol* 2009, **191:**1992-1993.
93. Yagi JM, Sims D, Brettin T, Bruce D, Madsen EL: **The genome of Polaromonas naphthalenivorans strain CJ2, isolated from coal tar-contaminated sediment, reveals physiological and metabolic versatility and evolution through extensive horizontal gene transfer.** *Environmental Microbiology* 2009, **11:**2253-2270.
94. Guo W, Wang Y, Song C, Yang C, Li Q, Li B, Su W, Sun X, Song D, Yang X, Wang S: **Complete genome of Pseudomonas mendocina NK-01, which synthesizes medium-chain-length polyhydroxyalkanoates and alginate oligosaccharides.** *J Bacteriol* 2011, **193:**3413-3414.
95. Fitz-Gibbon ST, Ladner H, Kim U-J, Stetter KO, Simon MI, Miller JH: **Genome sequence of the hyperthermophilic crenarchaeon Pyrobaculum aerophilum.** *Proc Natl Acad Sci U S A* 2002, **99:**984-989.
96. Cohen GN, Barbe Ve, Flament D, Galperin M, Heilig R, Lecompte O, Poch O, Prieur D, Qu\'erellou Je, Ripp R, et al: **An integrated analysis of the genome of the hyperthermophilic archaeon Pyrococcus abyssi.** *Molecular microbiology* 2003, **47:**1495-1512.
97. Fauvart M, Sánchez-Rodríguez A, Beullens S, Marchal K, Michiels J: **Genome Sequence of Rhizobium etli CNPAF512, a Nitrogen-Fixing Symbiont Isolated from Bean Root Nodules in Brazil.** *J Bacteriol* 2011, **193:**3158-3159.
98. Porter SL, Wilkinson Da, Byles ED, Wadhams GH, Taylor S, Saunders NJ, Armitage JP: **Genome sequence of Rhodobacter sphaeroides Strain WS8N.** *J Bacteriol* 2011, **193:**4027-4028.
99. Takeda H, Shimodaira J, Yukawa K, Hara N, Kasai D, Miyauchi K, Masai E, Fukuda M: **Dual two-component regulatory systems are involved in aromatic compound degradation in a polychlorinated-biphenyl degrader, Rhodococcus jostii RHA1.** *J Bacteriol* 2010, **192:**4741-4751.
100. Risso C, Sun J, Zhuang K, Mahadevan R, DeBoy R, Ismail W, Shrivastava S, Huot H, Kothari S, Daugherty S, et al: **Genome-scale comparison and constraint-based metabolic reconstruction of the facultative anaerobic Fe(III)-reducer Rhodoferax ferrireducens.** *BMC genomics* 2009, **10:**447.
101. Munk aC, Copeland A, Lucas S, Lapidus A, Del Rio TG, Barry K, Detter JC, Hammon N, Israni S, Pitluck S, et al: **Complete genome sequence of Rhodospirillum rubrum type strain (S1).** *Stand Genomic Sci* 2011, **4:**293-302.
102. Reeve W, Chain P, Ardley J, Nandesena K, Tiwari R, Malfatti S, Kiss H, Lapidus A, Co-A, Nolan M, et al: **Complete genome sequence of the Medicago microsymbiont Ensifer (Sinorhizobium) medicae strain WSM419.** *Stand Genomic Sci* 2010**:**77-86.
103. Pukall R, Lapidus A, Nolan M, Copeland A, Glavina Del Rio T, Lucas S, Chen F, Tice H, Cheng J-F, Chertkov O, et al: **Complete genome sequence of Slackia heliotrinireducens type strain (RHS 1).** *Stand Genomic Sci* 2009, **1:**234-241.
104. Sun Z, Chen X, Wang J, Zhao W, Shao Y, Wu L, Zhou Z, Sun T, Wang L, Meng H, et al: **Complete genome sequence of Streptococcus thermophilus strain ND03.** *J Bacteriol* 2011, **193:**793-794.

105. Chen L, Brugger K, Skovgaard M, Redder P, She Q, Torarinsson E, Greve B, Awayez M, Zibat A, Klenk HP, Garrett RA: **The genome of Sulfolobus acidocaldarius, a model organism of the Crenarchaeota.** *J Bacteriol* 2005, **187:**4992-4999.
106. Sikorski J, Lapidus A, Copeland A, Glavina T, Rio D, Nolan M, Lucas S, Chen F, Tice H, Cheng J-f, et al: **Complete genome sequence of Sulfurospirillum deleyianum type strain (5175T).** *Stand Genomic Sci* 2010**:**149-157.
107. Holtman CK, Chen Y, Sandoval P, Gonzales A, Nalty MS, Thomas TL, Youderian P, Golden SS: **High-Throughput Functional Analysis of the Synechococcus elongatus PCC 7942 Genome.** *DNA research* 2005, **12:**103-115.
108. Jenkins BD, Zehr JP, Gibson A, Campbell L: **Cyanobacterial assimilatory nitrate reductase gene diversity in coastal and oligotrophic marine environments.** *Environmental Microbiology* 2006, **8:**2083-2095.
109. Sieber JR, Sims DR, Han C, Kim E, Lykidis A, Lapidus AL, McDonnald E, Rohlin L, Culley DE, Gunsalus R, McInerney MJ: **The genome of Syntrophomonas wolfei: new insights into syntrophic metabolism and biohydrogen production.** *Environmental Microbiology* 2010, **12:**2289-2301.
110. McInerney MJ, Rohlin L, Mouttaki H, Kim U, Krupp RS, Rios-Hernandez L, Sieber J, Struchtemeyer CG, Bhattacharyya A, Campbell JW, Gunsalus RP: **The genome of Syntrophus aciditrophicus: life at the thermodynamic limit of microbial growth.** *Proc Natl Acad Sci U S A* 2007, **104:**7600-7605.
111. Zhaxybayeva O, Swithers KS, Lapierre P, Fournier GP, Bickhart DM, DeBoy RT, Nelson KE, Nesbo CL, Doolittle WF, Gogarten JP, Noll KM: **On the chimeric nature, thermophilic origin, and phylogenetic placement of the Thermotogales.** *Proc Natl Acad Sci U S A* 2009, **106:**5865-5870.
112. Muyzer G, Sorokin DY, Mavromatis K, Lapidus A, Clum A, Ivanova N, Pati A, D'Haeseleer P, Woyke T, Kyrpides NC: **Complete genome sequence of "Thioalkalivibrio sulfidophilus" HL-EbGr7.** *Stand Genomic Sci* 2011, **4:**23-35.
113. Beller HR, Chain PSG, Letain TE, Chakicherla A, Larimer FW, Richardson PM, Coleman MA, Wood AP, Kelly DP: **The Genome Sequence of the Obligately Chemolithoautotrophic, Facultatively Anaerobic Bacterium Thiobacillus denitrificans.** *J Bacteriol* 2006, **188:**1473-1488.
114. Scott KM, Sievert SM, Abril FN, Ball La, Barrett CJ, Blake Ra, Boller AJ, Chain PSG, Clark Ja, Davis CR, et al: **The genome of deep-sea vent chemolithoautotroph Thiomicrospira crunogena XCL-2.** *PLoS biology* 2006, **4:**e383.
115. Sievert SM, Scott KM, Klotz MG, Chain PSG, Hauser LJ, Hemp J, H\"ugler M, Land M, Lapidus A, Larimer FW, et al: **Genome of the epsilonproteobacterial chemolithoautotroph Sulfurimonas denitrificans.** *Applied and environmental microbiology* 2008, **74:**1145-1156.
116. Pappas KM, Kouvelis VN, Saunders E, Brettin TS, Bruce D, Detter C, Balakireva M, Han CS, Savvakis G, Kyrpides NC, Typas Ma: **Genome sequence of the ethanol-producing Zymomonas mobilis subsp. mobilis lectotype strain ATCC 10988.** *J Bacteriol* 2011, **193:**5051-5052.
